# Pterostilbene Sensitizes Cisplatin-Resistant Human Bladder Cancer Cells with Oncogenic *HRAS*

**DOI:** 10.3390/cancers12102869

**Published:** 2020-10-06

**Authors:** Yi-Ting Chen, Zi-Yi Huang, Han-Hsuan Tang, Wan-Ting Kuo, Shan-Ying Wu, Sheng-Hui Lan, Kai-Hsun Chang, Pin-Lun Lin, Ming-Fen Lee, Hung-Chi Cheng, Hsiao-Sheng Liu, Chi-Ying F. Huang, Guan-Cheng Huang, Chun-Li Su

**Affiliations:** 1 Department of Human Development and Family Studies, National Taiwan Normal University, Taipei 106, Taiwan; A5042@tpech.gov.tw (Y.-T.C.); e0208ric@ntnu.edu.tw (K.-H.C.); sp940921@gate.sinica.edu.tw (P.-L.L.); 2 Institute of Biopharmaceutical Sciences, National Yang-Ming University, Taipei 112, Taiwan; laduree120@ym.edu.tw (Z.-Y.H.); cyhuang5@ym.edu.tw (C.-Y.F.H.); 3 Graduate Program of Nutrition Science, School of Life Science, National Taiwan Normal University, Taipei 106, Taiwan; d09b48005@ntu.edu.tw; 4 Department of Microbiology and Immunology, College of Medicine, National Cheng Kung University, Tainan 701, Taiwan; s58041014@ncku.edu.tw (W.-T.K.); shanyingwu@tmu.edu.tw (S.-Y.W.); shlan@ym.edu.tw (S.-H.L.); hsliu713@kmu.edu.tw (H.-S.L.); 5 Department of Microbiology and Immunology, School of Medicine, College of Medicine, Taipei Medical University, Taipei 110, Taiwan; 6 Department of Life Sciences and Institute of Genome Sciences, National Yang-Ming University, Taipei 112, Taiwan; 7 Department of Nutrition, China Medical University, Taichung 404, Taiwan; leemf@cmu.edu.tw; 8 Department of Nutrition and Health Sciences, Chang Jung Christian University, Tainan 711, Taiwan; 9 Department of Biochemistry and Molecular Biology, College of Medicine, National Cheng Kung University, Tainan 701, Taiwan; hungchi@mail.ncku.edu.tw; 10 Center for Cancer Research, College of Medicine, Kaohsiung Medical University, Kaohsiung 807, Taiwan; 11 Department of Biochemistry, College of Medicine, Kaohsiung Medical University, Kaohsiung 807, Taiwan; 12 Division of Hemato-Oncology, Department of Internal Medicine, Yuan’s General Hospital, Kaohsiung 802, Taiwan

**Keywords:** pterostilbene, cisplatin resistance, *HRAS*, autophagy, senescence, gene database

## Abstract

**Simple Summary:**

RAS oncoproteins are considered undruggable cancer targets. Nearly 15% of cases of bladder cancer have a mutation of *HRAS*. The active HRAS contributes to the tumor progression and the risk of recurrence. Using our novel gene expression screening platform, pterostilbene was identified to sensitize cisplatin-resistant bladder cancer cells with *HRAS* alterations via RAS-related autophagy and cell senescence pathways, suggesting a potentially chemotherapeutic role of pterostilbene for cisplatin treatment of human bladder cancer with oncogenic *HRAS*. Pterostilbene is a safe and readily available food ingredient in edible plants worldwide. Exploiting the principle of combination therapy on pterostilbene-enhanced biosensitivity to identify undruggable molecular targets for cancer therapy may have a great possibility to overcome the cisplatin resistance of bladder cancer. Our data make *HRAS* a good candidate for modulation by pterostilbene for targeted cancer therapy in combination with conventional chemotherapeutic agents cisplatin plus gemcitabine.

**Abstract:**

Analysis of various public databases revealed that *HRAS* gene mutation frequency and mRNA expression are higher in bladder urothelial carcinoma. Further analysis revealed the roles of oncogenic HRAS, autophagy, and cell senescence signaling in bladder cancer cells sensitized to the anticancer drug cisplatin using the phytochemical pterostilbene. A T24 cell line with the oncogenic *HRAS* was chosen for further experiments. Indeed, coadministration of pterostilbene increased stronger cytotoxicity on T24 cells compared to *HRAS* wild-type E7 cells, which was paralleled by neither elevated apoptosis nor induced cell cycle arrest, but rather a marked elevation of autophagy and cell senescence in T24 cells. Pterostilbene-induced autophagy in T24 cells was paralleled by inhibition of class I PI3K/mTOR/p70S6K as well as activation of MEK/ERK (a RAS target) and class III PI3K pathways. Pterostilbene-induced cell senescence on T24 cells was paralleled by increased pan-RAS and decreased phospho-RB expression. Coadministration of PI3K class III inhibitor 3-methyladenine or MEK inhibitor U0126 suppressed pterostilbene-induced autophagy and reversed pterostilbene-enhanced cytotoxicity, but did not affect pterostilbene-elevated cell senescence in T24 cells. Animal study data confirmed that pterostilbene enhanced cytotoxicity of cisplatin plus gemcitabine. These results suggest a therapeutic application of pterostilbene in cisplatin-resistant bladder cancer with oncogenic *HRAS*.

## 1. Introduction

Bladder cancer is the ninth most common cancer worldwide [1], and is possibly caused by environmental factors including cigarette smoking and long-term nicotine exposure [2]. A combination of cisplatin and gemcitabine is the standard first-line treatment for patients with locally advanced and metastatic bladder cancer [3], whereas nephrotoxicity is a major dose-limiting side effect of cisplatin [4]. Among many developed platinum analogs, carboplatin exhibits significantly less nephrotoxicity while retaining tumoritoxicity [5]. Therefore, a combination of carboplatin and gemcitabine has been used as an alternative therapy for patients considered unfit to receive cisplatin [6]. However, when tumors from noninvasive fat and papillary urothelial neoplasia invade muscle or metastasize, recurrence is frequent and the clinical outcome is poor, with a median overall survival of approximately 14 months [3]. Therefore, there is a clinically unmet need to overcome resistance to treatment.

There are three main isoforms of RAS proteins: KRAS, HRAS, and NRAS. The mutation of RAS impairs the intrinsic GTPase activity of RAS and prevents GTP hydrolysis to GDP, causing the constitutively active GTP-bound RAS proteins to trigger downstream signaling, which leads to uncontrolled cell proliferation and formation of tumor [7]. Nearly 15% of cases of bladder cancer have a mutation of *HRAS*, and the active HRAS protein contributes to the tumor progression and is associated with the risk of recurrence [8]. Based on data in The Cancer Genome Atlas (TCGA), *HRAS* expression was significantly higher in clinical bladder cancer samples compared to healthy samples and samples from patients without the *HRAS* mutation [9]. Since the tumor-suppressive effect of RAS inhibitors can only be achieved at high concentrations, RAS inhibitors have not been used for clinical applications [9], and thus RAS oncoproteins are considered undruggable cancer targets, which implies that the proteins cannot be targeted pharmacologically [10].

Phytochemicals and other naturally occurring products have been extensively used for drug development. Pterostilbene, originally isolated from the heartwood of red sandalwood (*Pterocarpus santalinus*), is a polyphenol phytoalexin, a class of compounds naturally synthesized by plants during ultraviolet radiation and pathogen infection. Pterostilbene can be found in grapes and blueberries [11], and is a dimethylether analog of resveratrol. Resveratrol, a nonflavonoid polyphenolic compound present in peanuts, grapes, mulberries, cranberries, blueberries, and red wine, has been reported to possess anti-inflammatory properties [12]. Resveratrol was found to be well tolerated in rats fed 300 mg/kg/d for four weeks [13] and in patients with colorectal cancer and hepatic metastases who received 5 g daily for 14 days [14]. Intake of 250 mg/d pterostilbene for 6–8 weeks [15] or 450 mg extract containing pterostilbene (about 22%) for 14 days [16] was also considered to be safe in humans. It is noteworthy that the bioavailability of pterostilbene is 80–95%, which is significantly higher than that of other stilbene derivatives such as resveratrol (about 20%) [17,18].

The Connectivity Map (C-Map) is an effective, powerful, and widely popular analytical tool for drug discovery and determining mechanisms of action. It is based on differential expression gene signatures linking abundant small molecular compounds, genetic perturbations, and diversity diseases [19]. In 2017, the National Institutes of Health (Bethesda, MD, USA) and the Broad Institute (Cambridge, MA, USA) launched the C-Map and Library of Integrated Network-Based Cellular Signatures (LINCS) Unified Environment (CLUE), which expanded the database to tens of thousands of compounds and more than 5000 genetic perturbations. In addition, a lower cost and high-throughput transcriptome screening plate, named L1000, was introduced [20]. The experimental treatment responses of cells, tissues, or organisms can be queried to identify the corresponding transcriptome levels, which can describe a specific point of biologic states. Therefore, each perturbation has its own profile, which constitutes a unique pattern, and the computational algorithm can compare the degree of similarity among these patterns. C-Map employs Kolmogorov-Smirnov enrichment statistical (ES) [21] analysis, which yields a connectivity score representing the strength of enrichment between the query signature and each reference profile. A positive score shows a similar effect, whereas a negative score indicates an opposing mechanism. To obtain more detailed pathway information, target genes from the CLUE analysis can be connected to ConsensusPathDB (CPDB), an intercellular biology signaling metaplatform, which combines 30 public resources for mining of highly potential pathways and provides a network module [22,23]. In our study, pterostilbene gene expression signatures were retrieved from CLUE to obtain positively correlated target genes and then these target genes were used to query CPDB to predict the potential mechanism as well as to obtain a more comprehensive understanding of its fundamental mode of action.

In order to gain a deep understanding of a drug’s effects on different cancer cells, Profiling Relative Inhibition Simultaneously in Mixtures (PRISM) [24,25] provides a powerful analytical method involving pooled screening of mixtures cell lines, 24-nucleotide barcodes, with ID detection of individual cells, which serves to accelerate the experimental investigation of a drug’s inhibition ability. In this study, cell viability was measured by Luminex to determine the median fluorescence intensity (MFI). Then the log fold change values were compared with the dimethyl sulfoxide (DMSO)-treated negative controls to determine the ratio value. Finally, a log fold change value of <0.3 was found to indicate sensitivity to drug treatment in a given cell line. The Broad Institute merged their metadata, such as the Cancer Cell Line Encyclopedia (CCLE), Cancer Therapeutics Response Portal (CTRP), PRISM, and Deep RNAi Interrogation of Viability Effects (DRIVE), in the Dependency Map (DepMap) platform, which can be used to match genetic sequencing data and drug response information. Therefore, the correlations between compounds and genetic characteristics in a specific group can be obtained to discover a potential target or biomarker for cancer drug development.

In the present study, we utilized the unique profiling platform to identify a naturally occurring bioactive ingredient with the potential to overcome cisplatin resistance and also to identify the underlying molecular mechanism of action.

## 2. Results

### 2.1. Pterostilbene Was Predicted to Sensitize Cisplatin-Resistant Bladder Cancer Cells with HRAS Gene Alterations via RAS-Related Autophagy and Cell Senescence Pathways

**Figure 1:**
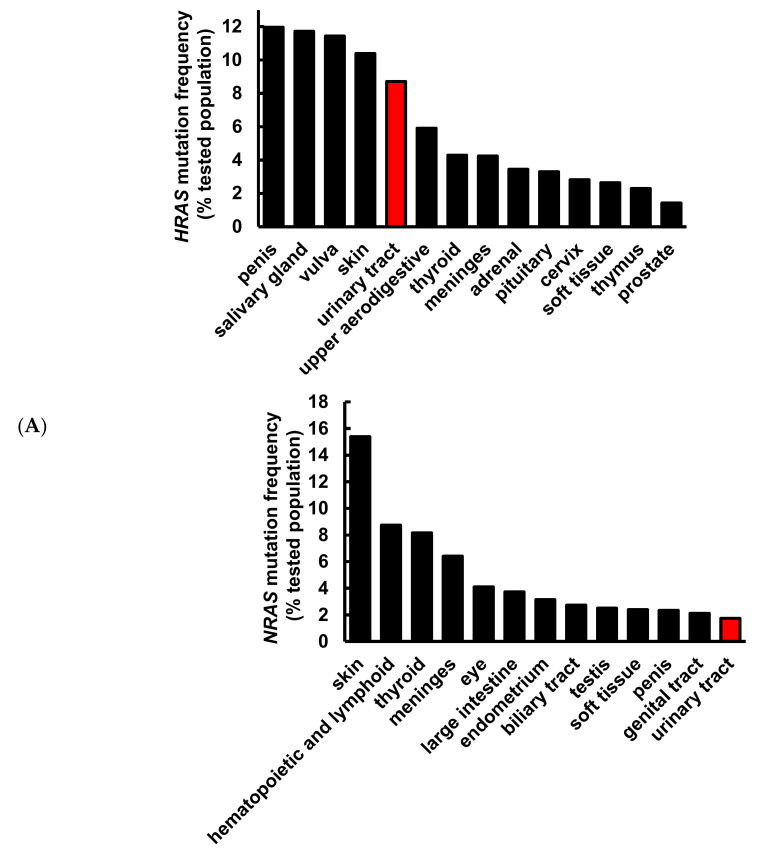

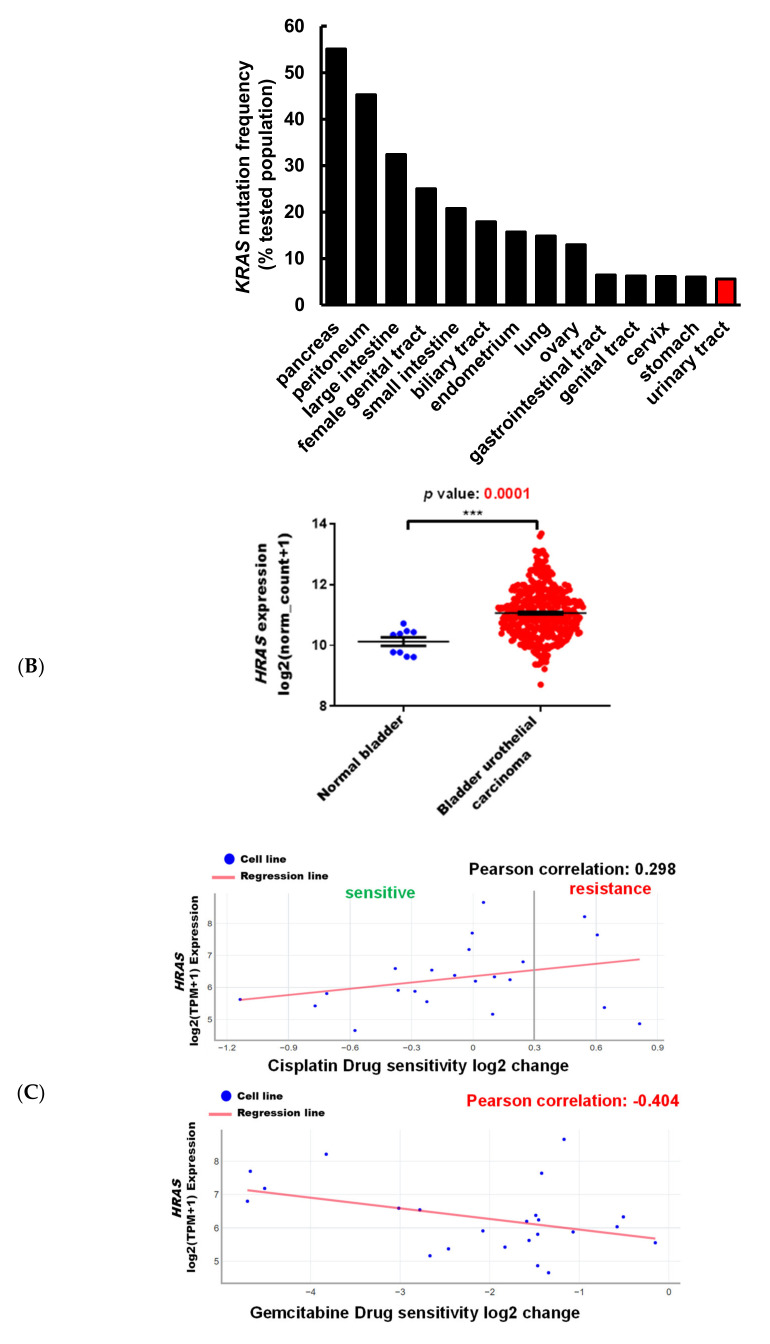

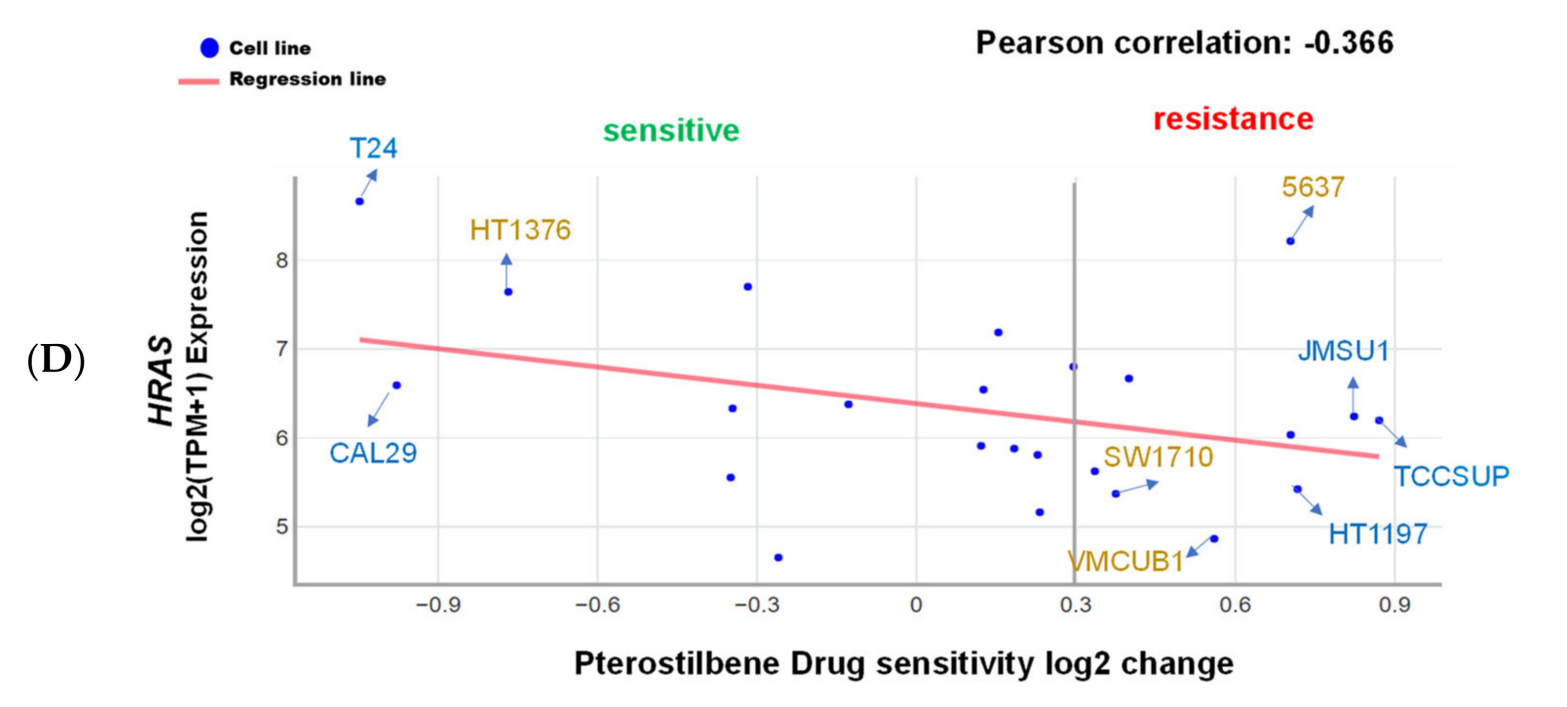
Pterostilbene was predicted to sensitize bladder cancer cells with oncogenic *HRAS* via bioinformatics analysis. (**A**) Mutation frequency of *HRAS*, *NRAS*, and *KRAS* among various primary tissue types. The data were retrieved from the COSMIC database version 91 (https://cancer.sanger.ac.uk/cosmic). (**B**) *HRAS* gene expression in normal bladder and bladder urothelial carcinoma tissues. The data were retrieved from the UCSC Xena (http://xena.ucsc.edu/). *** *p* = 0.0001 (**C**) *HRAS* expression increased sensitivity to gemcitabine in bladder cancer. The Profiling Relative Inhibition Simultaneously in Mixtures (PRISM) database obtained 4518 drug responses across 578 human cancer cell lines by using the barcode ID and the pooled screening of mixtures cell lines. The scatter plots from the PRISM show that gemcitabine treatment was negatively correlated with *HRAS* expression in bladder cancer. The higher the expression in a cell line, the better the sensitivity to gemcitabine, but not to cisplatin. The *x*-axis is the drug sensitivity score represented by log2 fold change. The lower the value is, the higher the sensitivity is. The cutoff is a score of <0.3. The *y*-axis is *HRAS* log2 expression data. (**D**) There was a slightly positive correlation between the sensitivity to pterostilbene and *HRAS* expression in bladder cancer cell lines. The scatter plot shows that pterostilbene may sensitize the cell lines with a higher expression of *HRAS*, suggesting the importance of cell line selection for better outcome of pterostilbene treatment. The golden color denotes cisplatin resistance and blue color denotes cisplatin sensitivity.

It has been well demonstrated that alterations in the *RAS* gene family convert the genes into active oncogenes, involving either point mutations or amplification of the wild-type gene [26]. Gene amplification results in an increase in protein expression. The data obtained from the Catalogue of Somatic Mutations in Cancer (COSMIC) and Xena indicate that *HRAS*, but not *NRAS* or *KRAS*, gene mutation frequency was high in urinary tract cancer as compared to other origins (Figure 1A), and *HRAS* gene expression was significantly greater in bladder urothelial carcinoma as compared to normal bladder samples (Figure 1B), suggesting the importance of *HRAS* oncogene in tumor progression of urinary tract cancer [8]. Results retrieved from the PRISM further revealed that although an elevated expression of *HRAS* significantly increased (Pearson correlation: <−0.4) the sensitivity (the lower the log2 change is, the higher the sensitivity is) of bladder cancer cell lines to gemcitabine, it had no beneficial effect against cisplatin (Figure 1C), implying that the basis of poor clinical outcome in patients receiving the current standard first-line combination therapy of cisplatin and gemcitabine [3] may be due to cisplatin-insensitivity of cells overexpressing the *HRAS* gene. To “drug” a previously “undruggable” *HRAS* cancer target, repurposing of the primary screen was performed using PRISM. A slightly positive correlation (Pearson correlation: −0.366) between the sensitivity to a naturally occurring compound pterostilbene and *HRAS* expression was discovered in bladder carcinoma cell lines (Figure 1D and Figure S1), suggesting that bladder cancer cells with a higher expression level of *HRAS* might be sensitive to pterostilbene.

**Figure 2:**
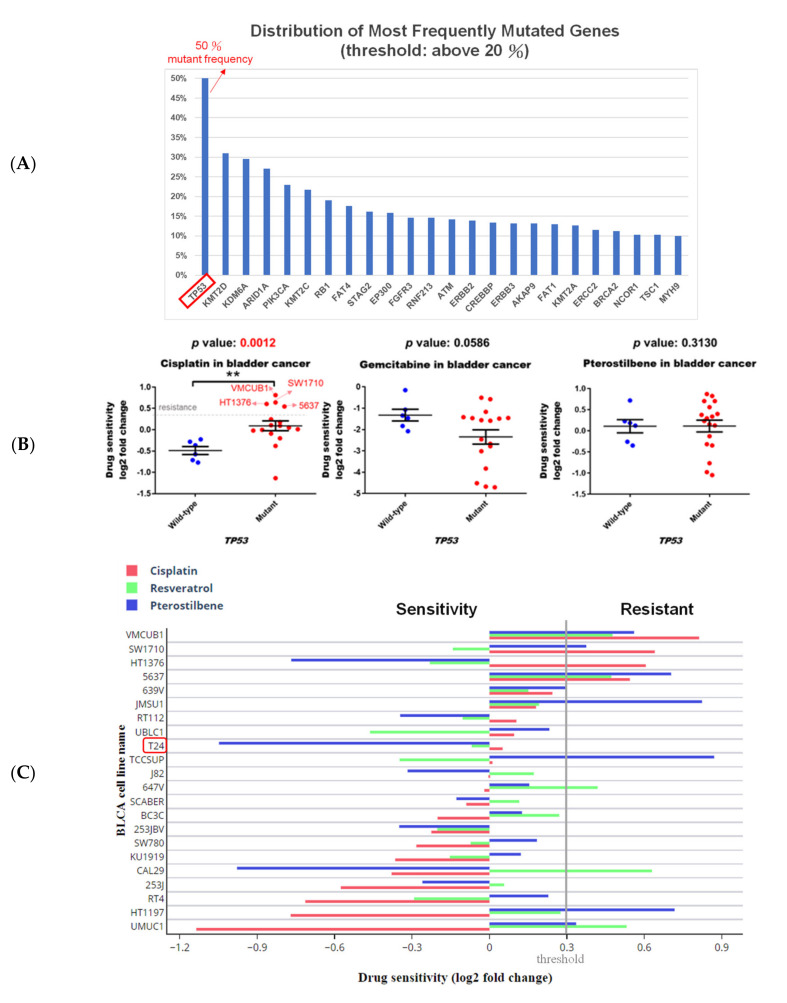

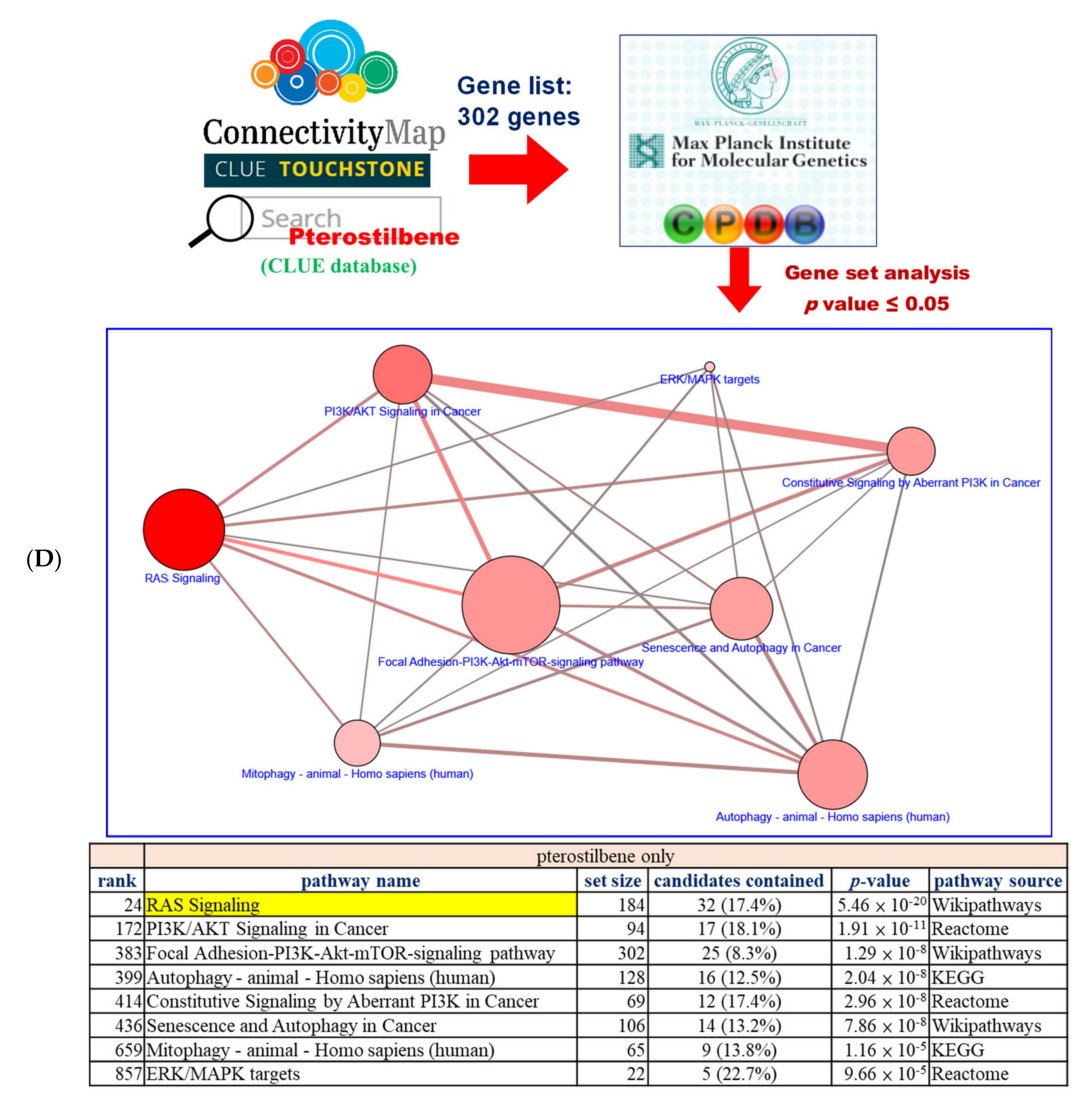
Pterostilbene was predicted to sensitize cisplatin-resistant bladder cancer cells by altering RAS-related autophagy and cell senescence pathways. (**A**) *TP53* is the most common mutated gene found in patients with bladder cancer. GDC-TCGA (https://portal.gdc.cancer.gov/) is a cancer patient database containing information related to drug treatment, survival, and biospecimen sequencing that can be used to obtain data on clinical cases and to assist basic research. The data on gene mutation frequency among bladder cancer patients showed that the most prevalent gene was *TP53* and almost 50% of patients with bladder cancer were characterized by the mutation. (**B**) The scatter plots show that the *TP53* wild-type was more sensitive to cisplatin, suggesting that *TP53* mutation may be one of the reasons for the resistance. Mutation of *TP53* had no significant effect on the sensitivity to gemcitabine or pterostilbene. The relationship between *TP53* and the drug response was analyzed. The *x*-axis features *TP53*. Each dot indicates a bladder cancer cell line. The blue dot denotes wild-type and the red dot denotes mutant. The *y*-axis is the cisplatin drug sensitivity score and the cutoff is <0.3 as indicated by the dashed gray line. Sensitivity to gemcitabine and pterostilbene was not significantly associated with *TP53* characteristics (Mann–Whitney test, one-tailed). ** *p* < 0.01. (**C**) Pterostilbene overcame cisplatin resistance in bladder cancer. The bar chart illustrates the responses of bladder cancer cells to three separate drugs. T24 was the most sensitive cell line to pterostilbene but not resveratrol. HT1376 was cisplatin-resistant but sensitive to pterostilbene, suggesting that pterostilbene overcame the resistance. The cutoff is <0.3. (**D**) Potential mechanism of pterostilbene predicted using the C-Map and Library of Integrated Network-Based Cellular Signatures (LINCS) Unified Environment (CLUE) and ConsensusPathDB (CPDB) databases. The network shows the correlation among PI3K/AKT signaling, ERK/MAPK pathway, RAS signaling, and autophagy. The size of each dot denotes the entity number of genes in the pathway. The line between two dots was calculated by the function of these two pathways to indicate the number of genes overlapping said pathways. The breadth of the line denotes the strength of the correlation between two dots. The table lists detailed information on the pathway analysis from CPDB. The “set size” denotes the total members in the pathway and the “candidates contained” is the overlapping number between our input gene list and the pathway CPDB calculated.

Besides the *HRAS* gene, *TP53* gene mutations have been reported to be the first genetic alterations in invasive bladder cancers [7]. Our search results from the Genomic Data Commons (GDC)-TCGA data portal (https://portal.gdc.cancer.gov/) showed that *TP53* is the most common mutated gene found in patients with bladder cancer (Figure 2A). Gene analysis using DepMap further revealed that although the *TP53* mutation confers resistance of bladder cancer cells to cisplatin, it has no significant effect on the sensitivity to gemcitabine or pterostilbene (Figure 2B), suggesting that pterostilbene may have an effect on bladder cancer cells despite the presence of the *TP53* genotype.

It is noteworthy that pterostilbene has the potential to sensitize cisplatin-resistant bladder cancer cells, possibly in an *HRAS*-related manner (Figure 2C). There are only four cisplatin-resistant cell lines (VMCUB1, SW1710, HT1376, and 5637; listed in decreasing order of resistance in Figure 2C) stored in the database, and they are all *TP53* mutants (Figure 2B). Two of the cell lines (VMCUB1 and SW1710) most resistant to cisplatin express a low level of *HRAS*, and the other two cell lines (HT1376 and 5637) with less resistance to cisplatin express a high level of *HRAS* (Figure 1D and Figure 2C), suggesting that the *TP53* mutant bladder cancer cells with a higher expression level of *HRAS* were less resistant to cisplatin, although the elevated *HRAS* expression had no beneficial effect in all of the cisplatin-treated bladder cancer cell lines stored in the database despite the *TP53* genotype (Figure 1C). It is noteworthy that two cisplatin-resistant cell lines (VMCUB1 and SW1710) with a lower expression level of *HRAS* were resistant to pterostilbene, and the cisplatin-resistant cell line expressing the highest level of *HRAS* (HT1376) was sensitive to pterostilbene (Figure 1D and Figure 2C), suggesting the role of *HRAS* gene expression in bladder cancer cells sensitized to cisplatin by pterostilbene.

To elucidate the underlying mechanism of pterostilbene, we retrieved target genes with similar gene expression signatures to pterostilbene from CLUE. Briefly, target genes exhibited >90 positive connectivity scores of PCLs, and the member genes belonging to these PCLs were used to query the CPDB database to reveal pterostilbene-induced pathways (Figure 2D). In the network diagram of the pathway, HRAS-related pathways, such as “RAS Signaling”, “PI3K/AKT Signaling in Cancer”, “Autophagy”, “ERK/MAPK targets”, and “Mitophagy”, were predicted to be involved (Figure 2D). It is noteworthy that the “RAS signaling” exhibited a greater involvement of pterostilbene-mediated action, implying that the response of pterostilbene treatment may be associated with *HRAS* expression level.

### 2.2. Pterostilbene-Enhanced Cytotoxic Response to Food and Drug Administration (FDA)-Approved Anticancer Drugs Was Indeed Associated with an Induction of Autophagy

**Figure 3:**
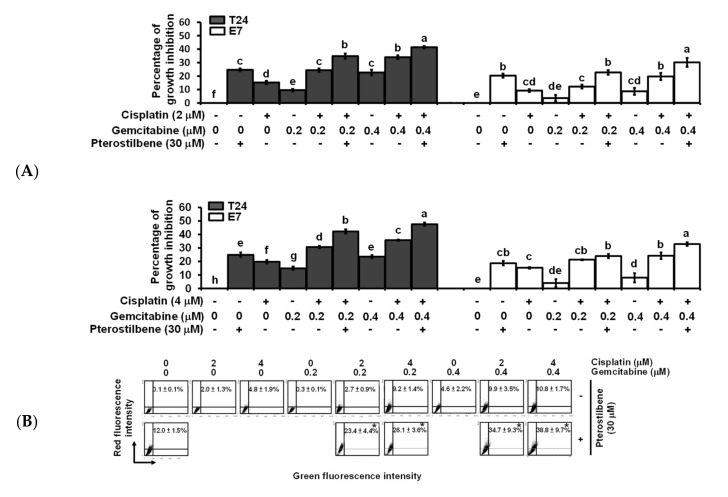

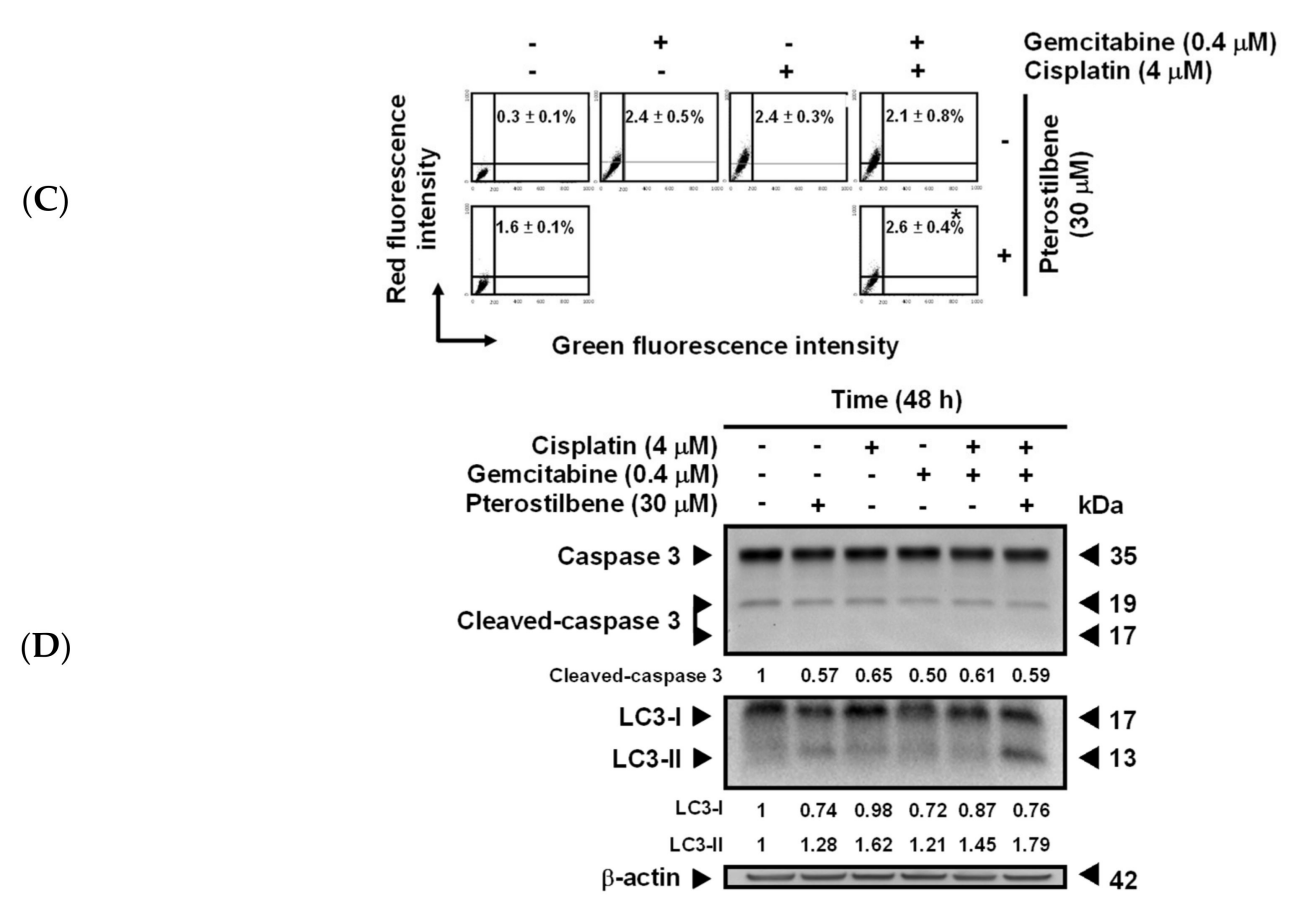
Effects of pterostilbene on biosensitivity, apoptosis, and autophagy of T24 cells treated with cisplatin and gemcitabine. (**A**) Pterostilbene enhanced the biosensitivity of T24 cells to a combination of cisplatin and gemcitabine. T24 and E7 cells were treated with the indicated concentration of compounds for 48 h. Cytotoxicity of the cells was determined using 3-[4,5-Dimethylthiazol-2-yl]-2,5-diphenyltetrazolium bromide (MTT) assay. Data of at least three independent experiments were quantified and are presented as means ± standard errors of the means (SEMs). Means in each plot and each cell line with different superscript letters are significantly different, *p* < 0.05. (**B**) Pterostilbene enhanced the autophagy of T24 cells treated with anticancer drugs. (**C**) Pterostilbene did not induce autophagy of E7 cells. After treatment for 48 h, the T24 and E7 cells were stained with acridine orange before flow cytometry. The percentages in the figure indicate the proportion of cells (upper two quadrants) with AVOs staining of at least three independent experiments. Data are presented as means ± SEMs. * denotes a significant difference compared to the group in the absence of pterostilbene, *p* < 0.05. (**D**) Protein expression of cleaved caspase 3 and LC3-II. After treatment for 48 h, the total protein of T24 cells was subjected to Western blot analysis. Anticaspase 3 and anti-LC3 antibodies served as probes. β-actin served as a loading control. The intensity of each protein expression band was quantified through densitometry normalization to that of β-actin, with the control level arbitrarily set to 1. Results are representative of three independent experiments.

To study the role of the *HRAS* cancer target and cisplatin resistance in bladder cancer, a T24 cell line with mutant HRAS (G12V, ATCC), expressing the highest level of *HRAS* (Figure 1D), and displaying the best sensitivity to pterostilbene was chosen for further experiments. To validate the results predicted using our novel gene expression platform (Figure 2C), the effect of pterostilbene on the biosensitivity of T24 cells to cisplatin/or carboplatin plus gemcitabine was investigated. The data indicate that pterostilbene, cisplatin, carboplatin, and gemcitabine separately displayed a time- and dosage-related increase in the cytotoxic response of T24 cells (Figure S2). The concentrations of each compound resulting in about 20–30% of the total growth inhibition were chosen for the drug combination assay [27]. E7 cells were used as a control cell line, which is immortalized by the human papillomavirus 16 E7 gene product that binds and alters RB and other proteins, in which the *HRAS* remains wild-type [28]. For most of the tested combinations, coadministration of pterostilbene significantly elevated (*p* < 0.05) the cytotoxic response of both T24 and E7 cell lines to cisplatin plus gemcitabine (Figure 3A) or carboplatin plus gemcitabine (Figure S3). It is noteworthy that the addition of pterostilbene increased the cytotoxic effect on T24 cells to a greater extent compared to that of E7 cells. These data confirm our prediction results (Figure 1D and Figure 2C) which suggest that pterostilbene may overcome cisplatin resistance in the human bladder cell line with *HRAS* alternations.

Flow cytometric analysis of T24 cells revealed that the addition of pterostilbene did not significantly (*p* > 0.05) increase the percentage of cells at the sub-G1 phase (representing apoptosis, programmed cell death type I), nor did it significantly change proportions of cells at each phase (G0/G1, S, and G2/M) of the cell cycle (Figure S4A,B), although S phase arrest was detected when T24 cells were treated with a lower concentration of cisplatin (2 μM) plus gemcitabine (0.2 or 0.4 μM). It is noteworthy that the addition of pterostilbene greatly increased (*p* < 0.05) the formation of acidic vesicular organelles (AVOs) in T24 cells under all of the tested combinations (Figure 3B and Figure S5A), and only a trivial elevation in AVOs was detected in E7 cells (Figure 3C and Figure S5B). The elevation of AVOs represents the formation of acidified autolysosomes at the later stage of the autophagy process [29] and is an indicator of autophagic degradation activity (autophagic flux). The pterostilbene-enhanced autophagy (Figure 3B and Figure S5A) but not apoptosis (Figure S4) in anticancer drug-treated T24 cells was confirmed by an increase in the expression of LC3-II but not change in the expression of cleaved caspase 3 using Western blot (Figure 3D and Figure S5C), respectively. These data confirm the prediction results (Figure 2C,D) and suggest that the pterostilbene-induced greater cytotoxic response of T24 cells to cisplatin/or carboplatin plus gemcitabine was paralleled by marked pterostilbene-induced autophagy in T24 cells, and the weaker pterostilbene-induced cytotoxic effect in E7 cells was concurrent with almost no pterostilbene-induced autophagy in E7 cells.

### 2.3. Pterostilbene-Induced Autophagy in T24 Cells Was Paralleled by an Activation of MEK/ERK and Class III PI3K, as Well as an Inhibition of Class I PI3K/mTOR/p70S6K

**Figure 4:**
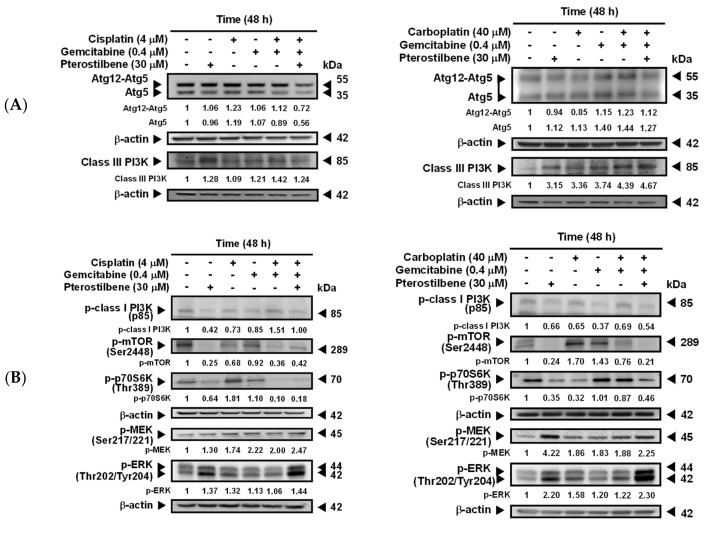

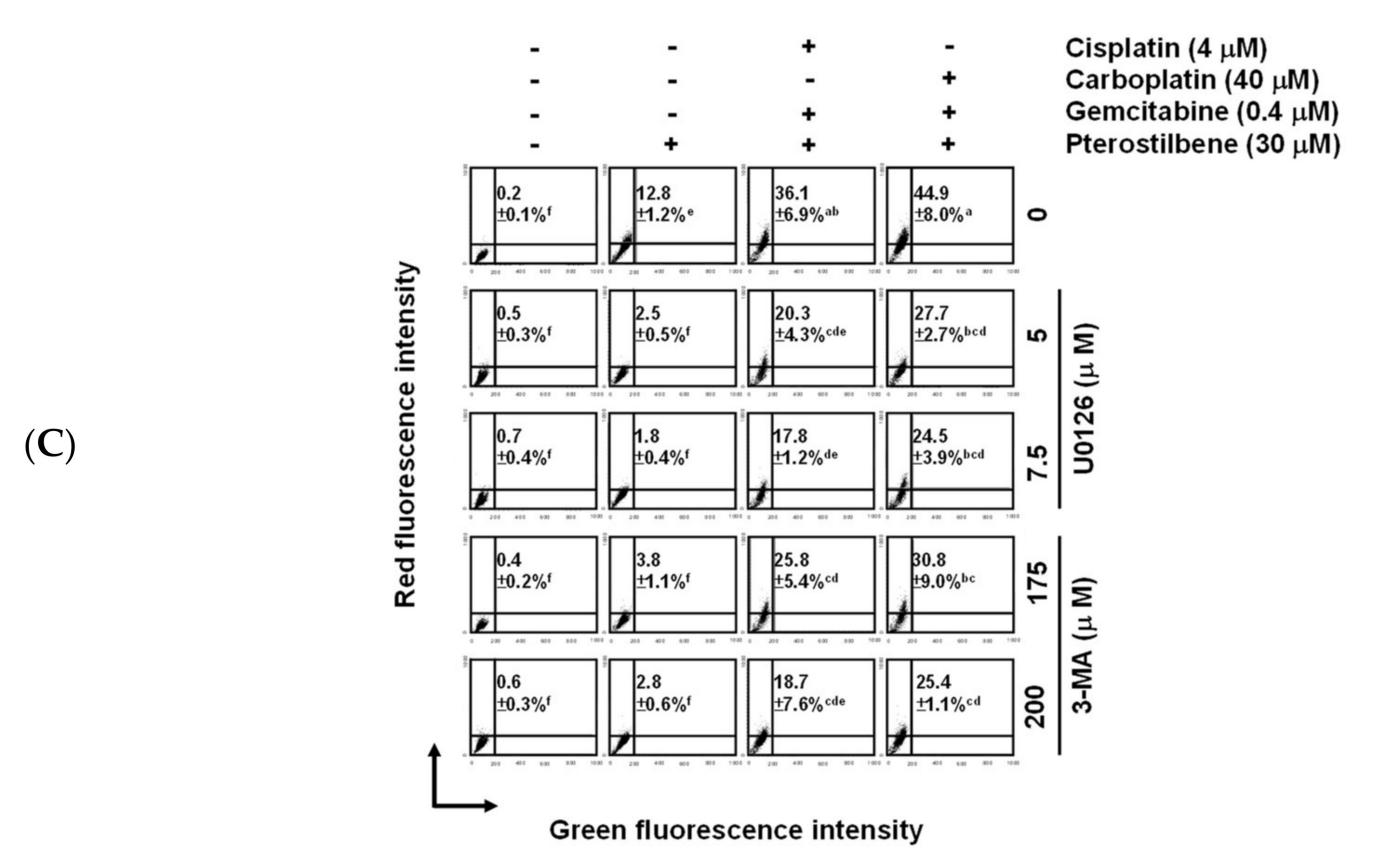
Pterostilbene changed the expression of autophagy-related proteins. (**A**) Expression of Atg5, Atg12-Atg5, and class III PI3K. (**B**) Expression of p-class I PI3K, p-mTOR, p-70S6K, p-MEK, and p-ERK. After treatment for 48 h, the total protein of T24 cells was subjected to Western blot analysis. Anti-Atg5, anti-class III PI3K, anti-p-class I PI3K, anti-p-mTOR, anti-p-70S6K, anti-p-MEK, and anti-p-ERK antibodies served as probes. β-actin served as a loading control. The intensity of each protein expression band was quantified through densitometry normalization to that of β-actin, with the control level arbitrarily set to 1. Results are representative of three independent experiments. (**C**) Effect of autophagy inhibitors on pterostilbene-induced autophagy. T24 cells were either pretreated with U0126 for 2 h prior to the addition of the indicated concentration of compounds for 48 h, or cotreated with 3-MA and the indicated concentration of compounds for 48 h. After treatment, the cells were stained with acridine orange before flow cytometry. The percentages in the figure indicate the proportion of cells (upper two quadrants) with AVOs staining of at least three independent experiments. Data are presented as means ± SEMs. Means with different superscript letters are significantly different, *p* < 0.05.

To validate the predicted signaling pathways (Figure 2C,D) involved in pterostilbene-induced autophagy in T24 cells, the expression of several autophagy-related molecules was determined. Beclin 1 is essential for the recruitment of Atg12 and Atg5 [30,31], and the covalent linkage of Atg12 to Atg5 is involved in the initiation of phagophore formation [32]. Beclin 1 also enhances autophagy by binding to class III PI3K to form Beclin 1 interactome [33]. As shown in Figure 4A, coadministration of pterostilbene did not further increase the expression of Atg12-Atg5 or Atg5, but elevated class III PI3K in T24 cells treated with carboplatin plus gemcitabine. These data suggest that pterostilbene-enhanced autophagy in the drug-treated T24 cells was not via activation of the Atg12-Atg5 pathway, but may involve class III PI3K. Phosphorylation of class I PI3K and its downstream molecules mTOR and p70S6K has been reported to inhibit autophagy [34,35], whereas phosphorylation of MEK and ERK induces autophagy [34]. The results shown in Figure 4B suggest that the enhancement of autophagy by the addition of pterostilbene is associated with a decrease of p-class I PI3K/p-mTOR/p-p70S6K expression and an enormous increase of p-MEK/p-ERK. Inhibition of pterostilbene-induced AVOs formation (Figure 4C) in the presence of MEK inhibitor (U0126) or PI3K class III inhibitor (3-methyladenine, 3-MA) confirmed the involvement of MEK/ERK and PI3K class III in the pterostilbene-induced autophagy of T24 cells. MEK/ERK is activated by RAS. Therefore, the constitutively active HRAS protein due to the *HRAS* mutation in T24 cells, as compared to the *HRAS* wild-type in E7 cells (Figure 3C), may strengthen autophagy induction (Figure 3B). These results confirm the role of mutant *HRAS* in pterostilbene-induced autophagy for sensitizing the cells to cisplatin (Figure 1D and Figure 2C,D).

### 2.4. Pterostilbene-Induced Senescence and Cytotoxic Autophagy in T24 Cells

**Figure 5:**
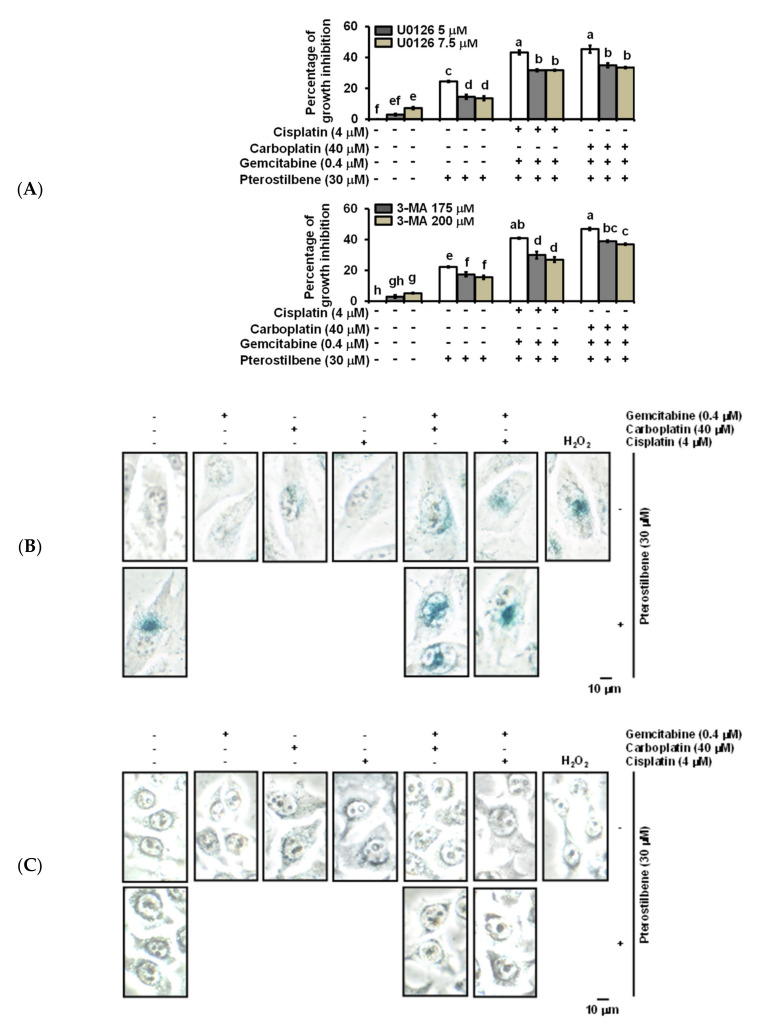

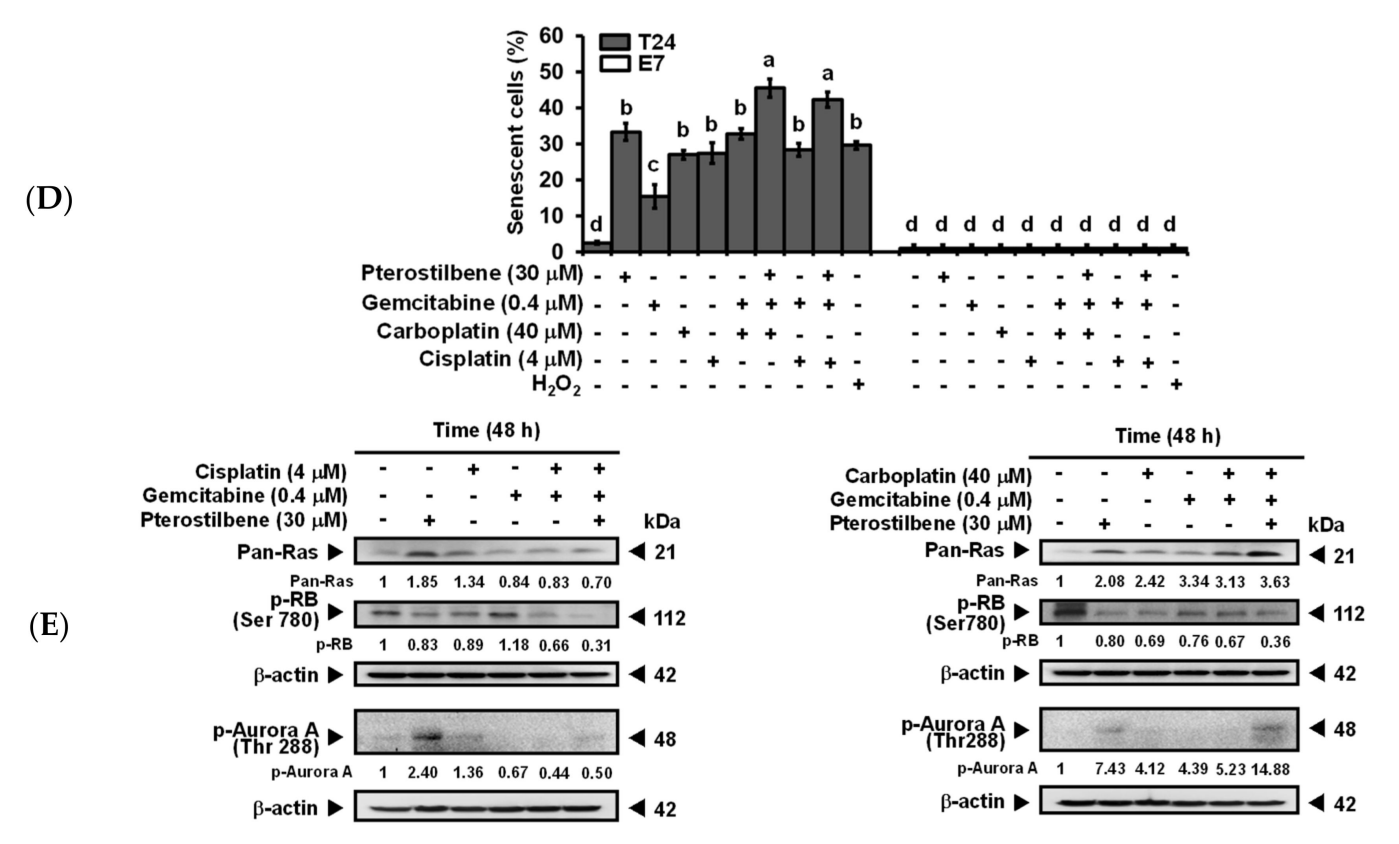
Pterostilbene induced the cytotoxic form of autophagy and enhanced senescence on T24 cells. (**A**) Effect of autophagy inhibitors on the population growth of T24 cells. T24 cells were either pretreated with U0126 for 2 h prior to the addition of the indicated concentration of compounds for 48 h, or cotreated with 3-MA and the indicated concentration of compounds for 48 h. After treatment, MTT was added to evaluate the cytotoxicity of the cells. Data of at least three independent experiments were quantified. (**B**) Induction of senescence on T24 cells. (**C**) No induction of senescence on E7 cells. (**D**) Quantification of senescence on both T24 and E7 cells. After treatment for 48 h, the activity of senescence-associated β-galactosidase was determined by the hydrolysis of X-Gal to yield a blue-colored product. The cells treated with H_2_O_2_ (stress-induced senescence) [44] were used as a positive control. The number of blue-colored cells of at least three independent experiments was quantified. At least 100 cells were counted for each experiment. Data are presented as means ± SEMs. Means in each plot with different superscript letters are significantly different, *p* < 0.05. (**E**) Expression of Pan-RAS, p-RB, and p-Aurora-A. After treatment for 48 h, the total protein of T24 cells was subjected to Western blot analysis. Anti-pan-RAS, anti-p-RB, and anti-p-Aurora-A antibodies served as probes. β-actin served as a loading control. The intensity of each protein expression band was quantified through densitometry normalization to that of β-actin, with the control level arbitrarily set to 1. Results are representative of three independent experiments.

Four faces of autophagy have been characterized [36], namely, cytoprotective (therapy resistance), cytotoxic (cell death promotion), cytostatic (senescence association), and nonprotective (no influence on sensitivity to therapy). To determine whether the pterostilbene-induced autophagy of T24 cells was cytotoxic, autophagy inhibitors U0126 and 3-MA (Figure 4C) were administered. As shown in Figure 5A, the addition of U0126 and 3-MA separately significantly decreased (*p* < 0.05) the cytotoxic response of T24 to the treatment with cisplatin/gemcitabine/pterostilbene or carboplatin/gemcitabine/pterostilbene, suggesting that pterostilbene-induced autophagy was cytotoxic. In order to establish whether induction of senescence (cytostatic autophagy; Figure 2C,D) was involved in the pterostilbene-enhanced biosensitivity of T24 cells to cisplatin/or carboplatin plus gemcitabine (Figure 3A and Figure S3), the activity of senescence-associated β-galactosidase [37] was determined. As shown in Figure 5B–D, pterostilbene, cisplatin, carboplatin, gemcitabine, and H_2_O_2_ (a positive control) separately induced senescence in T24 cells, but not E7 cells. The addition of pterostilbene further promoted the degree of the induced senescence in the T24 cells treated with cisplatin/or carboplatin plus gemcitabine (Figure 5D). The lack of induction of senescence in E7 cells was paralleled by a minute induction of autophagy (Figure 3C and Figure S5B). Previous reports indicate that two main signaling pathways initiate and maintain senescence: p53/p21/retinoblastoma protein (RB) and p16/RB, in which RB hypophosphorylation leads to a blockade of S phase entry and an arrest of the cell cycle [38]. A high level of RAS signaling also induces senescence by causing the accumulation of p16 [39]. Figure 5E indicates that the decrease in the RB phosphorylation in response to pterostilbene may have been due to the higher expression of pan-RAS in cells treated with carboplatin plus gemcitabine, since T24 is a p53 mutated cell line [40]. Nevertheless, in p53-deficient mice, overexpression of Aurora-A may contribute to senescence induction [41]. Aurora-A is an oncoprotein. Phosphorylation is required for Aurora-A kinase activity [42] and Aurora-A is autophosphorylated in its activation loop on Thr288 [43]. Figure 5E shows that pterostilbene elevated the expression of phospho-Thr288-Aurora-A in T24 cells treated with carboplatin plus gemcitabine. These data suggest that the pterostilbene-enhanced senescence in T24 cells treated with cisplatin/or carboplatin plus gemcitabine may proceed, at least in part, via hypophosphorylation of RB, overexpression of RAS, and/or activation of Aurora-A. The constitutively active mutant HRAS signaling in *HRAS* mutated T24 cells may benefit the enhancement of senescence in response to pterostilbene compared to *HRAS* wild-type E7 cells.

### 2.5. Pterostilbene Strengthened the Cisplatin/Gemcitabine-Induced Cytotoxicity and Reduced Tumorigenesis in T24 Xenograft-Bearing Mice without Exhibiting Physiologically Significant Side Effects

**Figure 6:**
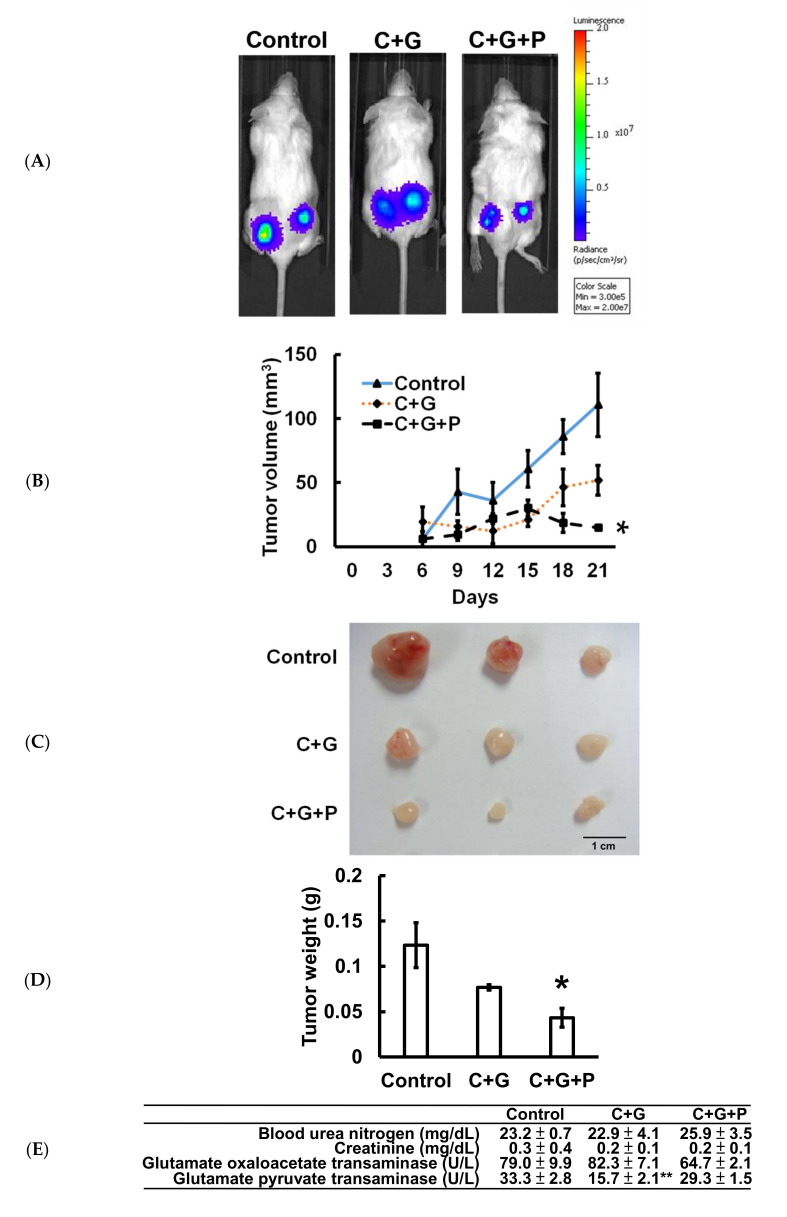

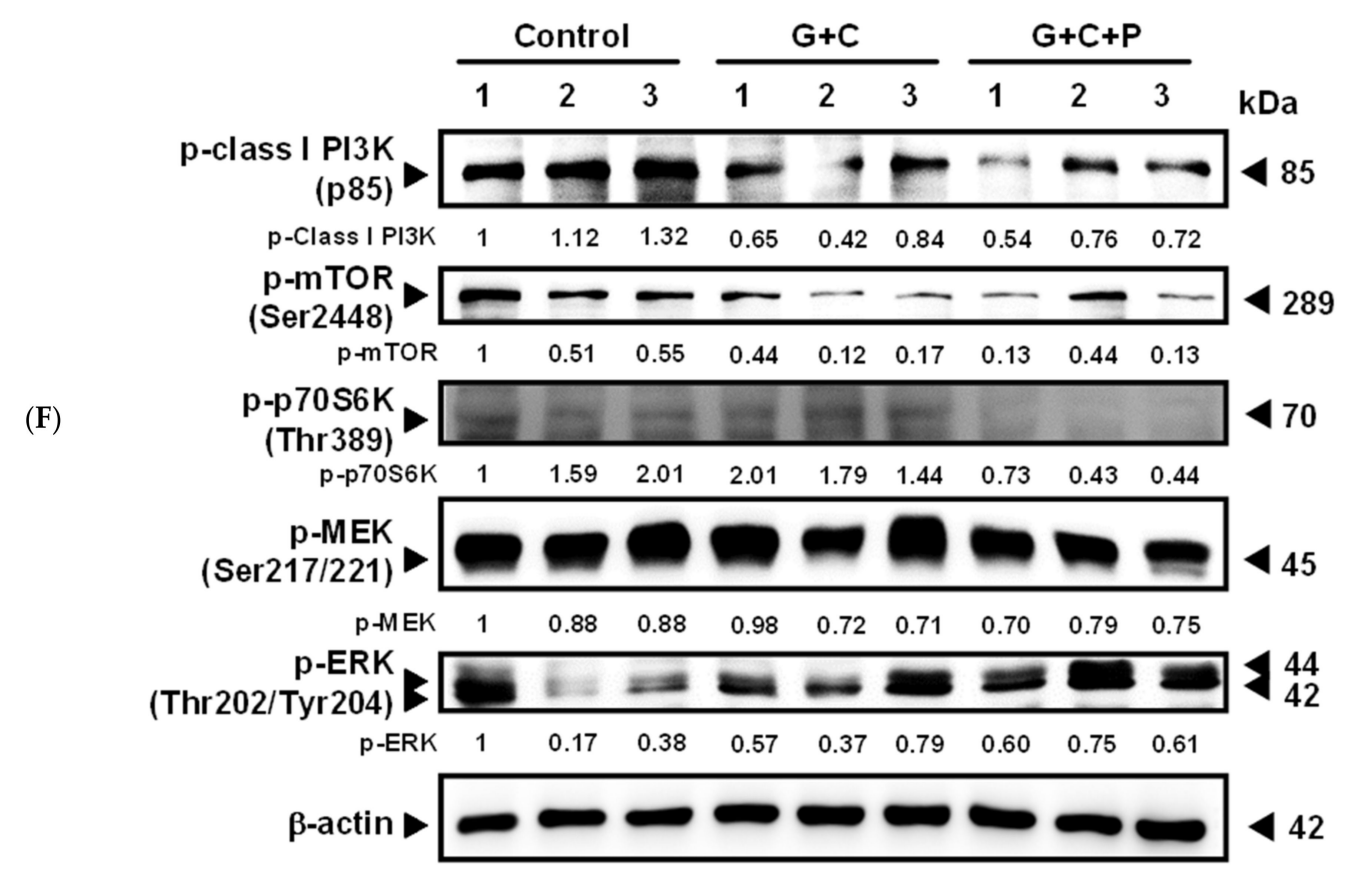
Cotreatment of pterostilbene significantly reduced tumorigenesis of T24 cells in mice. (**A**) Representative bioluminescence images of T24-Luc tumor-bearing mice on day 21 indicated the growth of tumor in the control group, cisplatin plus gemcitabine (C+G) group, and combination of cisplatin, gemcitabine, and pterostilbene (C+G+P) group by the IVIS imaging system. (**B**) Tumor volume was measured at the indicated time points. (**C**) Three representative bladder tumors on Day 21 are shown in the photographs. (**D**) The weights of the three representative bladder tumors were measured on Day 21. (**E**) The plasma biochemical parameters of mice at the end of the treatment. All samples were compared to control to evaluate the *p*-value. Data are presented as means ± SEMs. (*n* = 4 each group). * denotes *p* < 0.05, ** denotes *p* < 0.01. (**F**) Protein expression in tumors. The intensity of each protein expression band was quantified through densitometry normalization to that of β-actin, with the control level arbitrarily set to 1. C denotes cisplatin; G denotes gemcitabine; P denotes pterostilbene.

**Figure 7:**
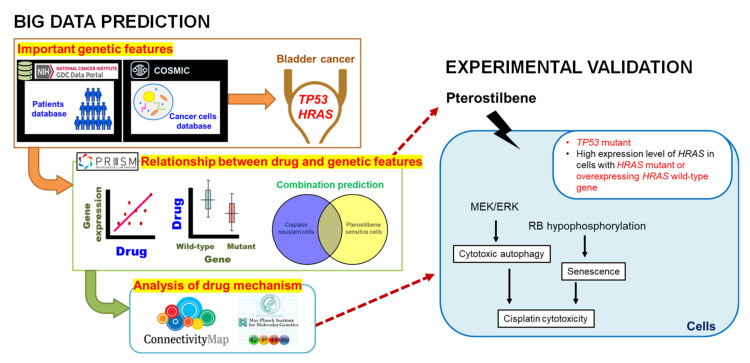
The effect of pterostilbene on cisplatin-resistant human bladder cancers with oncogenic *HRAS*.

T24-Luc cells were subcutaneously implanted into NOD-SCID mice to confirm the antitumor effect of pterostilbene in vivo. The data indicate that treatment of cisplatin/gemcitabine not only slightly suppressed the growth of T24-Luc cells (Figure 6A), but also reduced tumor volume (Figure 6B), tumor size (Figure 6C), and tumor weight (Figure 6D) on Day 21 compared to the control. A significant reduction (*p* < 0.05) was observed when pterostilbene was combined with cisplatin/gemcitabine. Moreover, plasma biochemical parameters of mice were examined to determine if pterostilbene caused nephrotoxicity or hepatotoxicity in mice. As shown in Figure 6E, blood urea nitrogen (BUN), creatinine, glutamate oxaloacetate transaminase (GOT), and glutamate pyruvate transaminase (GTP) were nonsignificantly different between the mice that received cisplatin/gemcitabine/pterostilbene and the control mice. These results demonstrate that the cotreatment of pterostilbene increased the chemosensitivity of grade III human bladder cancer T24 cells to cisplatin/gemcitabine without causing considerable physiological toxicity in mice, suggesting the application of pterostilbene in human bladder cancer. The decrease in tumor expression of p-class I PI3K/p-mTOR/p-p70S6K and increase of p-ERK (Figure 6F) validated the results using bioinformatics analysis and confirmed the observation in vitro (Figure 7), further highlighting the role of mutant *HRAS* in cisplatin sensitivity of T24 xenografts in response to pterostilbene.

## 3. Discussion

By using gene databases, pterostilbene was predicted to sensitize cisplatin-resistant bladder cancers with oncogenic *HRAS*. Among the bladder cancer cell lines stored in the PRISM database, the most effective cell line against pterostilbene is T24 (Figure 2C), a grade III human bladder cancer cell with mutant HRAS protein (G12V, ATCC). The second and third most sensitive cell lines (CAL29 and HT1376, respectively; Figure 2C) to pterostilbene overexpressed *HRAS* (Figure 1D and Supplementary Figure S1), and six (TCCSUP, JMSU1, HT1197, VMCUB1, SW1710, and UMUC1; listed in decreasing order of resistance in Figure 2C) out of the seven pterostilbene-resistant cell lines stored in the database expressed low levels of *HRAS* (Figure 1D and Figure S1). The average of the *HRAS* mRNA expression level (6.31) was set as a cutoff value to divide cell lines according to the higher or lower expression of *HRAS* (Figure S1). These results demonstrate that overexpression of the *HRAS* gene correlated well with the sensitivity of pterostilbene in bladder cancer cells, and mutation of the *HRAS* gene was associated with the highest sensitivity to pterostilbene. Since oncogenic *HRAS* contributes to tumor progression and is associated with the risk of recurrence [8], and RAS oncoproteins have been recognized as an undruggable cancer target [10], our results warrant further investigation as they suggest the potential of pterostilbene to overcome the resistance to cisplatin in bladder cancer with oncogenic *HRAS* and may fulfill an unmet clinical need.

The PRISM database was done by high-throughput screening across 4000 drugs and 600 cancer cell lines, which defined the IC_50_ and AUC (area under the curve) as the cell viability and drug response, respectively. Based on these numerous data, we could realize the sensitivity of cells on specific drug treatments at a glance. The variability in pterostilbene’s effect in the various cell lines (Figure 1D and Figure 2C) may be, at least in part, due to the genotype of *TP53* and expression level of *HRAS* (Figure 7). The involvement of p53 on the induction of autophagy and senescence has been reported via activation of DRAM to promote the formation of autophagosomes/autolysosomes [45] and stimulation of p53/p21/RB causing RB hypophosphorylation to block S phase entry [38], respectively. The *TP53* mutation (lost-of-function mutations) leads to less activation of autophagy and/or senescence, conferring resistance of bladder cancer cells to cisplatin. In a p53 mutated cell line with a high expression level of *HRAS*, either due to *HRAS* mutation (gain-of-function mutations) or overexpressing the wild-type gene [26], pterostilbene utilizes the high expression level of HRAS protein to elevate the RAS signaling for induction of autophagy and senescence by causing the activation of MEK/ERK and accumulation of p16 [39], respectively.

Malnutrition is linked to poorer clinical outcomes in cancer patients [46]. Around 30% of cancer deaths are due to diet and five behavioral risk factors according to a WHO report. The putative nutraceutical benefits of certain compounds have led to the supernutritional intake of certain foods. A number of naturally occurring compounds derived from plant sources have been well characterized and have demonstrated various remarkable antitumor properties [47]. Several representative diet-related bioactive food constituents, such as curcumin, resveratrol, paclitaxel, and quercetin, may lead to cancer cell death by regulating some of the core pathways of programmed nonapoptotic cell death, such as autophagy [47]. Potential strategies for treating cancer have been suggested to manipulate the autophagy process in order to improve the treatment of cancers and overcome chemoresistance [34].

Recently, the induction of autophagy has been reported in pterostilbene-treated human lung [48,49], breast [50,51], and bladder [52] cancer cells. Inhibition of autophagy enhanced the death of these cancer cells, suggesting that pterostilbene-induced autophagy is cytoprotective (resistant to therapy). However, our gene analysis results reveal that pterostilbene-induced autophagy has the potential to sensitize *HRAS* mutant bladder cancer cells to cisplatin (Figure 2C), suggesting the pterostilbene-induced autophagy is cytotoxic. Administration of autophagy inhibitor 3-MA and U0126 separately indeed reduced the cytotoxic response of T24 cells to treatment with cisplatin/gemcitabine/pterostilbene or carboplatin/gemcitabine/pterostilbene (Figure 5A), further validating that pterostilbene induced a cytotoxic form of autophagy. The use of a higher concentration of 3-MA (200 μM) increased the percentages of apoptotic cells treated with pterostilbene or carboplatin/gemcitabine/pterostilbene (Figure S6A,B), suggesting that further suppression of autophagy may cause induction of apoptosis.

Cellular senescence (or merely senescence) is a permanent state of cell cycle arrest that serves to protect against cancer in mammals by preventing neoplastic transformation [53]. Unlike the process of apoptosis which removes cells, senescence arrests these cells in a functional but nondividing state, which may provide a persistent signal to facilitate cancer cell immune surveillance. Therefore, senescence may confer a benefit by reducing the incidence of cancer [54]. In the present study, pterostilbene increased the degree of senescence in the T24 cells treated with cisplatin/or carboplatin plus gemcitabine (Figure 5D). However, the use of 3-MA or U0126 did not change the activity of senescence-associated β-galactosidase activity (Figure S7), implying that pterostilbene-enhanced senescence may not be associated with cytostatic autophagy (a function of autophagy associated with senescence). The E7 cells are from an RB knockdown cell line [28], leading to RB inactivation and cellular senescence blockage [55]. Therefore, the absence of RB expression observed in E7 cells (Supplementary Figure S8) may also explain why no induction of senescence occurred in the cells (Figure 5C,D).

Currently, there are two major types of senescence, namely, replicative senescence (telomere-dependent senescence) and stress-induced premature senescence (telomere-independent senescence), which links to the changes in telomere length and environmental insults, respectively. Oncogene-induced senescence is another kind that is associated with the activation of certain oncogenes, in which families of *RAS* and *RAF* have been described most extensively. Activation of oncogenes has been reported to drive cells to develop both replicative senescence and stress-induced premature senescence [56]. Although senescence plays a role in tumor-suppression, senescent cells in tumor stroma have been observed to promote cancer progression mediated by the effects of the senescence-associated secretory phenotype. In this regard, selective elimination of the tumorigenic senescent cells may further benefit cancer treatment [53].

Both senescence and autophagy are in essence cytoprotective responses for maintaining cellular homeostasis in response to several sources of stress. Recent studies have found that senescence [57] and autophagy [58] inhibit cancer progression under certain circumstances, which makes them attractive targets for cancer treatment. However, the relationship between autophagy and senescence and whether autophagy positively regulates senescence are context-dependent and inconclusive [59,60,61]. Goehe and colleagues [61] reported that senescence and autophagy happened collaterally, and inhibition of the latter delayed but not abrogated senescence. Studies also provided evidence that pterostilbene induced the senescence of cancer cells in a p53-dependent fashion [62,63]. Though T24 cells carry mutated *TP53*, the mutation of which results in the in-frame deletion of tyrosine at the residue 126 of p53, preventing it from binding with SV40 large T antigen [64] but may still possess some functions of the wild-type counterparts [65]. The effect of the in-frame deletion of p53 on senescence awaits further investigation.

Oncoprotein Aurora-A regulates centrosomal and microtubule activity, and controls chromosome segregation [66]. Our previous data revealed that the phytochemical curcumin significantly inhibited Aurora-A gene expression and subsequent kinase activity, which caused or contributed to the failure of various mitotic events and G2/M mitotic arrest in human bladder cancer T24 cells [67], chemoresistant nonmetastatic human breast cancer MCF-7 cells, and highly metastatic human breast cancer MDA-MB-231 cells [68]. However, in the present study, pterostilbene elevated the expression of phospho-Thr288-Aurora-A in T24 cells treated with carboplatin plus gemcitabine (Figure 5E), suggesting that pterostilbene increased Aurora-A activity in T24 cells. It is noteworthy that T24 is a p53 mutated cell line [40]. Overexpression of Aurora-A has been reported to induce senescence in p53-deficient mice [41]. In contrast, Aurora-A inhibition has also been reported to induce senescence of glioblastoma neurosphere [69] and many types of human cells in vitro and in vivo [70]. Since both up- and downregulation of Aurora-A are linked to the generation of senescence, the changes in Aurora-A expression by pterostilbene may only play a minor role in pterostilbene-induced senescence.

## 4. Materials and Methods

### 4.1. Cell Culture

The chemicals used in this study were obtained from Sigma (St. Louis, MO, USA) unless otherwise indicated. Pterostilbene (>98% purity) was obtained from Cayman Chemical (Ann Arbor, MI, USA). Cisplatin (Abiplatin^®^, 0.5 g/mL) was obtained from Abic Ltd. (Netanya, Israel). Carboplatin (Paraplatin^®^, 10 mg/mL) was obtained from Corden Pharma Latina S. P. A. (Sermoneta, LT, Italy). Gemcitabine (Gemzar^®^) was obtained from Lilly USA, LLC (Indianapolis, IN, USA). Pterostilbene was dissolved in DMSO. Gemcitabine was dissolved in water. Grade III human bladder cancer T24 cells (ATCC, Rockville, MD, USA) and immortalized human uroepithelial E7 cells (a gift from Dr. Nan-Haw Chow; National Cheng Kung University Hospital, Tainan, Taiwan) [71] were cultured in complete Dulbecco’s modified Eagle medium (DMEM; GIBCO BRL, Gaithersburg, MD, USA) in an incubator at 37 °C with a humidified atmosphere of 5% CO_2_. All of the compounds were diluted to the final desired concentrations with complete DMEM. Control cells were treated with an equal amount of DMSO (0.1%) without pterostilbene, cisplatin, carboplatin, or gemcitabine.

### 4.2. Analysis of Mutation Frequency and Expression of the Genes

The mutation frequency of *HRAS*, *KRAS*, and *NRAS*, among various primary tissue types, was retrieved from the COSMIC database (version 91; https://cancer.sanger.ac.uk/cosmic). The gene expression data of *HRAS* in normal and malignant bladder tissues were obtained from the genotype-tissue expression project and TCGA, respectively, and data were also retrieved from the Xena platform (http://xena.ucsc.edu/) developed by the University of California, Santa Cruz (CA, USA).

### 4.3. Regression Test

To observe the relationships between drug response and genetic characteristics across various cancer cell lines, the DepMap website (https://depmap.org/portal/) was used to query the drugs as well as the *TP53* and *HRAS* genes. The “Online Graph Maker” was used to plot the scatter diagram and to calculate the Pearson correlation (https://chart-studio.plotly.com/create/#/). Based on the report in PRISM [24], the threshold of the sensitivity score was set at <0.3. Each dot represents a bladder cancer cell line and the red line indicates the regression state.

### 4.4. Cytotoxicity Assay

Cytotoxicity of the phytochemicals and/or anticancer drugs on T24 and E7 cells was determined using a modified colorimetric MTT assay [68]. After treatment, the medium containing the tested compounds was removed to prevent color interference during the MTT assay. The absorbance was measured at 590 nm using an ELISA Reader (Synergy HT, BioTek, Highland Park, Winooski, VT, USA). The cytotoxicity was calculated according to previous descriptions [72].

### 4.5. Flow Cytometric Analysis

To determine the induction of apoptosis and cell cycle arrest, cells were stained with propidium iodide (40 μg/mL) for 30 min before flow cytometry [73]. To determine the induction of autophagy, cells were stained with acridine orange (1.5 μg/mL) for 15 min [74]. The formation of AVOs was determined by green and red fluorescence using flow cytometry. Accumulation of AVOs results in increased red light emission. The cells were sorted by FACS Calibur (Becton Dickinson, Lexington, KY, USA). Data were analyzed using ModFit LT™ (Verity Software House, Topsham, ME, USA) and WinMDI 2.8 (Windows Multiple Document Interface Flow Cytometry Application; The Scripps Institute, Flow Cytometry Core Facility, La Jolla, CA, USA).

### 4.6. Western Blot Analysis

Protein contents in whole-cell lysates were resolved using 8–12% SDS-PAGE and subsequently transferred to polyvinylidene fluoride membranes (Perkin Elmer, Santa Clara, CA, USA). Western blotting was performed [67] using antibodies obtained from the following sources: rabbit monoclonal anti-class III PI3K, anti-phospho-Aurora-A (Thr288), anti-phospho-MEK1/2 (Ser221)(166F8), anti-phospho-RB (Ser780), rabbit polyclonal anticaspase 3, anti-phospho-mTOR (Ser2448), anti-class I phospho-PI3K p85 (Tyr458)/p55 (Tyr199), mouse monoclonal anti-phospho-p44/42 MAPK (ERK 1/2) (Thr202/Tyr204), and anti-phospho-p70S6K (Thr389) were obtained from Cell Signaling Technology, Inc. (Danvers, MA, USA); rabbit polyclonal anti-LC3B and rabbit monoclonal anti-APG5L/Atg5 were obtained from Abcam (Cambridge Science Park, UK); mouse monoclonal anti-pan-RAS was obtained from Calbiochem (San Diego, CA, USA); and mouse monoclonal anti-β-actin was obtained from Sigma; goat antirabbit and antimouse conjugated horseradish peroxidase secondary antibodies were obtained from Millipore Corp. (Billerica, MA, USA). The whole western blots (uncropped blots) could see Figure S9.

### 4.7. Senescence Assay

Cell senescence was determined by the activity of senescence-associated β-galactosidase using a senescence detection kit (BioVision Inc., Milpitas, CA, USA), according to the manufacturer’s instructions. The images were examined using a light microscope (CK-2; Olympus, Tokyo, Japan) with ISCapture (WS-500; Tucsen, China).

### 4.8. Animal Study

As previously described [72], female Nod/LTSZ Prkdc (SCID) mice at 4–6 weeks of age obtained from the Laboratory Animal Center of National Cheng Kung University (Tainan, Taiwan) were housed at the center in a pathogen-free, temperature-controlled and air-conditioned environment with a 10/14 h light/dark cycle. Food and water were provided ad libitum. T24-Luc cells were established by transfecting pEGFP-Luc into T24 cells using Lipofectamine 2000™ (Thermo Fisher Scientific, Waltham, MA, USA), and the clones were then selected by using DMEM supplemented with G418 (600 μg/mL; Merck, Darmstadt, Germany). The T24-Luc cells (1 × 10^7^ cells/mice) were injected subcutaneously to the flank of the mice on Day 0, and the mice were then randomly divided into three groups on Day 3. The mice either intraperitoneally received vehicle (control group), cisplatin (1 mg/kg) plus gemcitabine (20 mg/kg), or combination of cisplatin (1 mg/kg), gemcitabine (20 mg/kg), and pterostilbene (30 mg/kg) every other day from Day 3 to Day 18. Mice were monitored for gross anatomical changes. Tumor growth was measured with a caliper and an IVIS imaging system (Caliper Life Sciences, Hopkinton, MA, USA). Tumor volume was calculated by using the formula: *V* = 0.52 × *a^2^* × *b* (*a* indicating the smallest superficial diameter; *b* indicating the largest superficial diameter) [75]. Plasma biochemical parameters, including BUN, creatinine, GOT, and GPT were examined according to the protocol of FUJI DRI-CHEM 4000i (FUJIFILM Corporation, Minato-ku, Tokyo, Japan). The animal experimental protocol adhered to the regulation of the Animal Protection Act of Taiwan, and all animal experiments were approved by the Laboratory Animal Care and Use Committee of National Cheng Kung University (No. 104166).

### 4.9. Statistical Analysis

The results are expressed as means ± SEMs. The data were analyzed by Student’s *t*-test and one-way ANOVA. Differences among groups were analyzed by Duncan’s new multiple range test (SPSS software, version 14.0). *p* < 0.05 was considered significant. 

## 5. Conclusions

Our novel gene expression screening platform provided a short-cut, which identified a strategy for overcoming drug resistance. Pterostilbene is a safe and readily available food ingredient in edible plants worldwide. Our data indicate that pterostilbene-enhanced biosensitivity of *HRAS* mutant T24 cells against cisplatin/carboplatin plus gemcitabine was associated with induction of the cytotoxic form of autophagy (promoting cell death) and cell senescence. Pterostilbene further decreased tumor growth in mice that received cisplatin plus gemcitabine, suggesting a potentially chemotherapeutic role of pterostilbene for cisplatin treatment of human bladder cancers with oncogenic *HRAS*.

Recently, several combination therapies have been approved in oncology and a number of combinations are in late-phase clinical trials. These regimens have been reported to substantially improve the efficacy of the treatments [76]. Exploiting the principle of combination therapy on pterostilbene-enhanced biosensitivity to identify undruggable molecular targets, such as HRAS, for cancer therapy may have a great possibility to overcome the cisplatin resistance of bladder cancer. Our data make *HRAS* a good candidate for modulation by pterostilbene for targeted cancer therapy in combination with conventional chemotherapeutic agents cisplatin plus gemcitabine [77].

## Supplementary Materials

The following are available online at https://www.mdpi.com/2072-6694/12/10/2869/s1, Figure S1: The information of Figure 1D on *HRAS* expression and cisplatin resistance of bladder, Figure S2: Time- and dosage-related increase in the cytotoxic response of T24 cells to pterostilbene and each of the anticancer drugs, Figure S3: Pterostilbene enhanced biosensitivity of T24 cells to combination of carboplatin and gemcitabine, Figure S4: The effect of pterostilbene on apoptosis of T24 cells treated with anticancer drugs, Figure S5: Effects of pterostilbene on autophagy of T24 and E7 cells treated with carboplatin and gemcitabine, Figure S6: Effect of autophagy inhibitors on apoptosis of T24 cells, Figure S7: Effect of autophagy inhibitors on senescence of T24 cells, Figure S8: The phosphorylation of RB protein in T24 and E7 cells, Figure S8: The phosphorylation of RB protein in T24 and E7 cells, Figure S9: the whole western blots (uncropped blots).

## Abbreviations

AVOs	acidic vesicular organelles
BUN	blood urea nitrogen
CCLE	Cancer Cell Line Encyclopedia
CLUE	C-Map and LINCS Unified Environment
C-Map	Connectivity Map
COSMIC	Catalogue of Somatic Mutations in Cancer
CPDB	ConsensusPathDB
CTRP	Cancer Therapeutics Response Portal
DepMap	Dependency Map
DMEM	Dulbecco’s modified Eagle medium
DMSO	dimethyl sulfoxide
DRIVE	Deep RNAi Interrogation of Viability Effects
ES	enrichment statistic
FDA	Food and Drug Administration
GDC	Genomic Data Commons
GOT	glutamate oxaloacetate transaminase
GPT	glutamate pyruvate transaminase
LINCS	Library of Integrated Network-Based Cellular Signatures
MFI	median fluorescence intensity
MTT	3-[4,5-Dimethylthiazol-2-yl]-2,5-diphenyltetrazolium bromide
PRISM	Profiling Relative Inhibition Simultaneously in Mixtures
SEMs	standard errors of the means
TCGA	The Cancer Genome Atlas
