# Supplementary material for: Pterostilbene Sensitizes Cisplatin-Resistant Human Bladder Cancer Cells with Oncogenic HRAS
Source: Cancers (Basel). 2020 Oct 6;12(10):2869. doi: 10.3390/cancers12102869 (PMC7650649; doi:10.3390/cancers12102869)
Supplement: Supplementary file 1 [file cancers-12-02869-s001.zip › cancers-920015-Figure S1-S8.pdf]

cancers

Supplementary Materials

Pterostilbene Sensitizes Cisplatin-Resistant Human Bladder Cancer Cells with Oncogenic *HRAS*

Yi-Ting Chen, Zi-Yi Huang, Han-Hsuan Tang, Wan-Ting Kuo, Shan-Ying Wu, Sheng-Hui Lan, Kai-Hsun Chang, Pin-Lun Lin, Ming-Fen Lee, Hung-Chi Cheng, Hsiao-Sheng Liu, Chi-Ying F. Huang, Guan-Cheng Huang and Chun-Li Su

Cell line name	*HRAS* expression level		Drug response	
	log2(TPM+1)	annotation	pterostilbene	cisplatin
T24	8.6611	higher	-1.0479	0.0510
5637	8.2142	higher	0.7038	0.5439
J82	7.7012	higher	-0.3173	-0.0041
HT1376	7.6434	higher	-0.7680	0.6057
647V	7.1866	higher	0.1541	-0.0197
639V	6.8014	higher	0.2953	0.2438
KMBC2	6.6680	higher	0.3998	#N/A
CAL29	6.5920	higher	-0.9781	-0.3793
BC3C	6.5426	higher	0.1259	-0.2012
SCABER	6.3775	higher	-0.1280	-0.0897
RT112	6.3315	higher	-0.3461	0.1043
JMSU1	6.2413	lower	0.8235	0.1802
TCCSUP	6.1967	lower	0.8706	0.0117
BFTC905	6.0345	lower	0.7041	#N/A
KU1919	5.9107	lower	0.1215	-0.3653
SW780	5.8795	lower	0.1838	-0.2833
RT4	5.8089	lower	0.2280	-0.7136
UMUC1	5.6241	lower	0.3356	-1.1358
253JBV	5.5543	lower	-0.3496	-0.2252
HT1197	5.4229	lower	0.7172	-0.7703
SW1710	5.3709	lower	0.3751	0.6406
UBLC1	5.1627	lower	0.2320	0.0955
VMCUB1	4.8644	lower	0.5603	0.8123
253J	4.6531	lower	-0.2598	-0.5759
Average (cutoff)	6.3101			

Figure S1. The information of Fig. 1D on *HRAS* expression and cisplatin resistance of bladder cancer cell lines. The green color labeled for resistance cell lines.

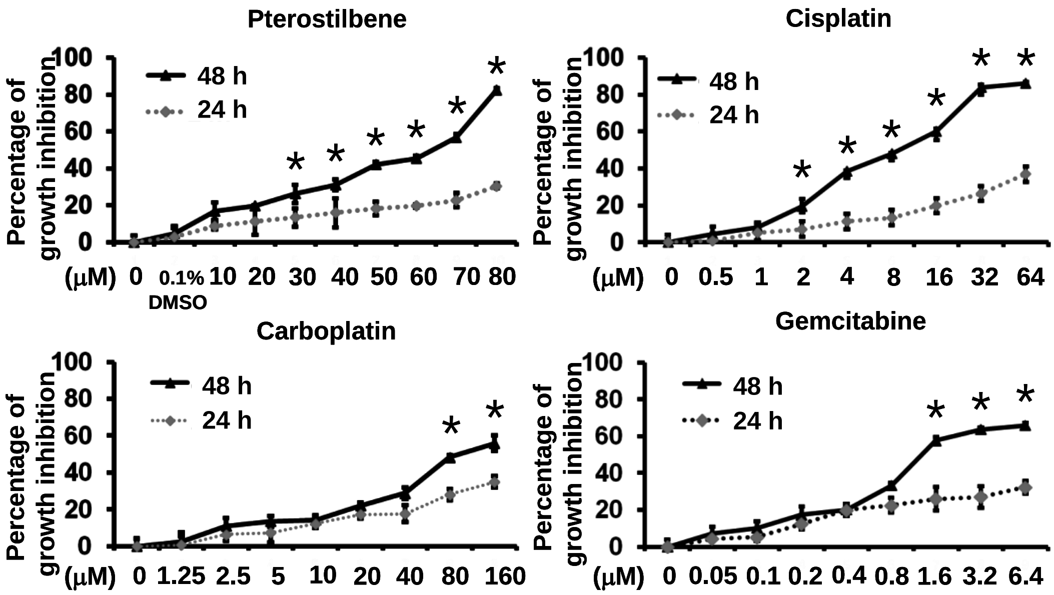

Figure S2. Time- and dosage-related increase in the cytotoxic response of T24 cells to pterostilbene and each of the anti-cancer drugs. Cells were treated with the indicated concentration of pterostilbene, cisplatin, carboplatin, and gemcitabine separately for 24 or 48 h. Cytotoxicity in the cells was determined by MTT assay. Data of at least three independent experiments were quantified and are presented as means ± SEMs. * denotes a significant difference between 24 and 48 h, $P < 0.05$.

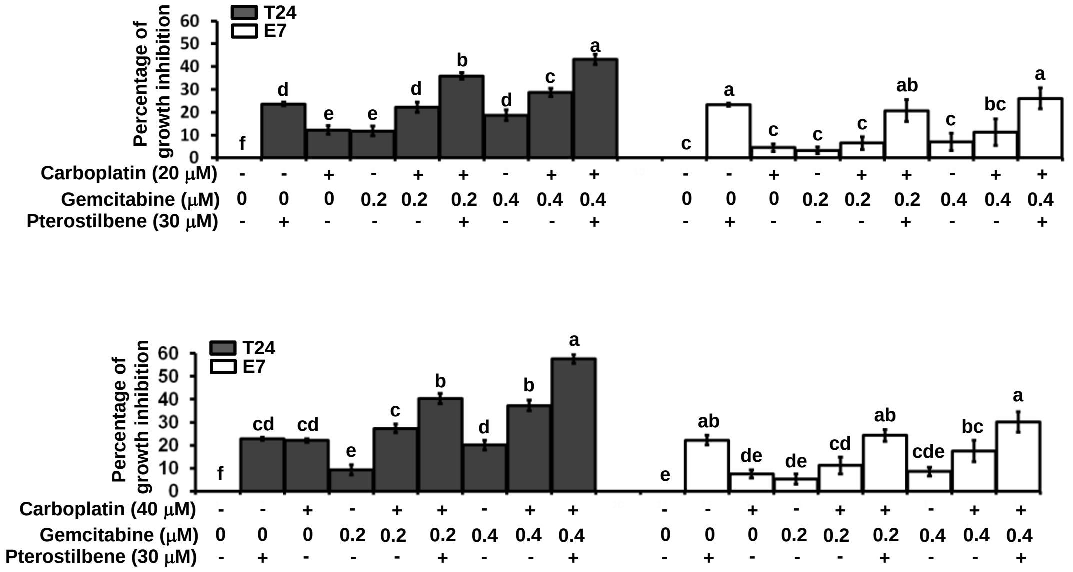

Figure S3. Pterostilbene enhanced biosensitivity of T24 cells to combination of carboplatin and gemcitabine. T24 and E7 cells were treated with the indicated concentration of compounds for 48 h. Cytotoxicity in the cells was determined using MTT assay. Data of at least three independent experiments were quantified and are presented as means ± SEMs. Means in each plot and each cell line with different superscript letters are significantly different, $P < 0.05$.

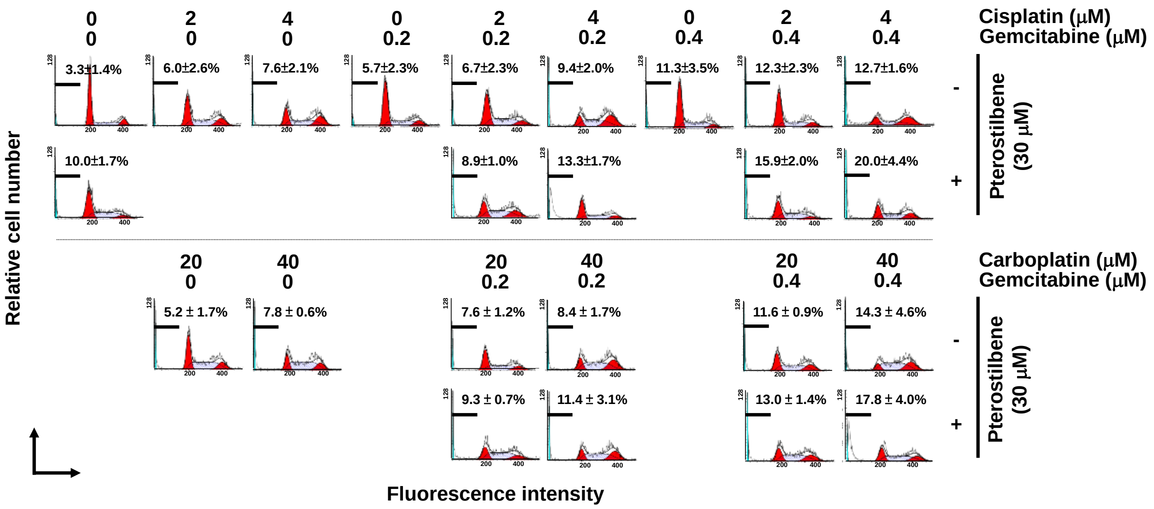

(**A**)

Time (h)	48				
Cisplatin (2 μM)	-	-	-	-	
Gemcitabine (μM)	-	-	0.2	0.4	
Pterostilbene (30 μM)	-	+	-	-	
			%		
Sub-G1	3.3 ± 1.4^{b}	10.0 ± 1.7^{ab}	5.7 ± 2.3^{ab}	11.3 ± 3.5^{a}	
G0/G1	69.8 ± 1.3^{a}	52.6 ± 1.0^{b}	60.8 ± 2.7^{ab}	56.6 ± 2.9^{b}	
S	10.1 ± 0.6^{b}	17.8 ± 1.0^{a}	14.2 ± 0.8^{b}	13.3 ± 0.2^{b}	
G2/M	16.9 ± 0.5^{a}	19.6 ± 1.6^{a}	19.3 ± 1.1^{a}	18.8 ± 0.9^{a}	
Cisplatin (2 μM)	+	+	+	+	+
Gemcitabine (μM)	-	0.2	0.2	0.4	0.4
Pterostilbene (30 μM)	-	-	+	-	+
Sub-G1	6.0 ± 2.6^{b}	6.7 ± 2.3^{b}	8.9 ± 1.0^{ab}	12.3 ± 2.3^{ab}	15.9 ± 2.0^{a}
G0/G1	52.4 ±2.4^{a}	52.9 ± 0.8^{a}	42.2 ± 3.1^{b}	43.8 ± 6.8^{ab}	40.2 ± 2.9^{b}
S	13.2 ±0.5^{c}	13.3 ± 0.6^{c}	20.5 ± 1.2^{ab}	15.7 ± 3.1^{bc}	18.5 ± 1.1^{a}
G2/M	28.4 ± 1.8^{a}	27.1 ± 1.2^{a}	28.5 ± 3.1^{a}	28.3 ± 1.6^{a}	25.4 ± 2.4^{a}
Cisplatin (4 μM)	+	+	+	+	+
Gemcitabine (μM)	-	0.2	0.2	0.4	0.4
Pterostilbene (30 μM)	-	-	+	-	+
Sub-G1	7.6 ± 2.1^{b}	9.4 ± 2.0^{b}	13.3 ± 1.7^{ab}	12.7 ± 1.6^{ab}	20.0 ± 4.4^{a}
G0/G1	38.4 ± 2.6^{a}	31.0 ± 1.0^{a}	37.3 ± 3.4^{a}	33.8 ± 1.4^{a}	33.7 ± 2.7^{a}
S	13.7 ± 0.4^{b}	21.7 ± 2.1^{ab}	24.1 ± 2.3^{a}	21.5 ± 6.4^{ab}	22.6 ± 5.1^{ab}
G2/M	40.4 ± 2.8^{a}	37.9 ± 0.3^{a}	25.2 ± 5.8^{b}	31.9 ± 4.3^{ab}	23.7 ± 4.6^{b}
Carboplatin (20 μM)	+	+	+	+	+
Gemcitabine (μM)	-	0.2	0.2	0.4	0.4
Pterostilbene (30 μM)	-	-	+	-	+
Sub-G1	5.2 ± 1.7^{b}	7.6 ± 1.2^{a}	9.3 ± 0.7^{a}	11.6 ± 0.9^{a}	13.0 ± 1.4^{a}
G0/G1	43.6 ± 4.1^{a}	37.9 ± 5.9^{a}	37.8 ± 0.3^{a}	39.3 ± 1.2^{a}	35.8 ± 3.1^{a}
S	17.3 ± 0.7^{ab}	26.7 ± 6.7^{a}	20.6 ± 0.2^{a}	16.4 ± 0.6^{b}	20.5 ± 1.2^{a}
G2/M	33.9 ± 1.9^{a}	27.8 ± 1.4^{b}	32.3 ± 0.5^{ab}	32.7 ± 1.2^{ab}	30.7 ± 3.4^{ab}
Carboplatin (40 μM)	+	+	+	+	+
Gemcitabine (μM)	-	0.2	0.2	0.4	0.4
Pterostilbene (30 μM)	-	-	+	-	+
Sub-G1	7.8 ± 0.6^{ab}	8.4 ± 1.7^{b}	11.4 ± 3.1^{ab}	14.3 ± 4.6^{a}	17.8 ± 4.0^{a}
G0/G1	36.7 ± 1.6^{a}	34.7 ± 3.5^{a}	33.0 ± 1.9^{a}	30.8 ± 1.0^{a}	34.6 ± 2.6^{a}
S	17.4 ± 0.7^{ab}	25.8 ± 7.4^{a}	23.6 ± 0.9^{a}	20.8 ± 3.0^{ab}	21.3 ± 1.2^{a}
G2/M	38.1 ± 1.7^{a}	31.1 ± 6.0^{a}	32.0 ± 5.5^{a}	34.1 ± 3.0^{a}	26.3 ± 5.3^{a}

(**B**)

Figure S4. The effect of pterostilbene on apoptosis of T24 cells treated with anti-cancer drugs. (A) Induction of apoptosis. The percentages in the figure indicate the proportion of apoptotic cells. (B) Quantification of cell cycle profiles and the percentages of cells at the sub-G1 phase, representing apoptotic cells. After treatment for 48 h, the T24 cells were stained with propidium iodide before flow cytometry. Data of at least three independent experiments were quantified and are presented as

means ± SEMs. Means in each cell cycle and each concentration of cisplatin and carboplatin with different superscript letters are significantly different, $P < 0.05$.

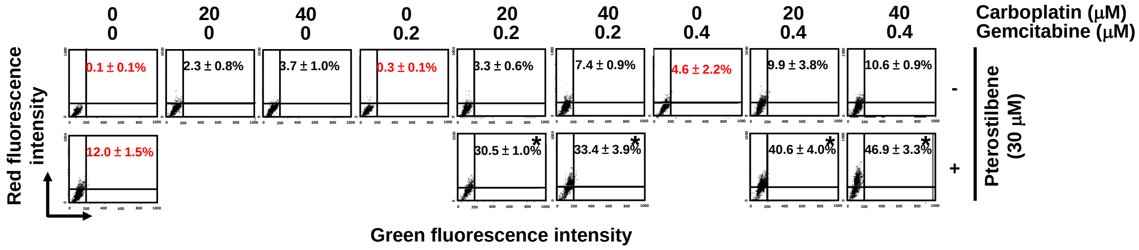

(A)

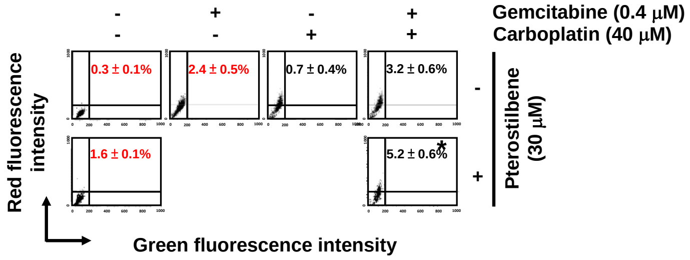

(B)

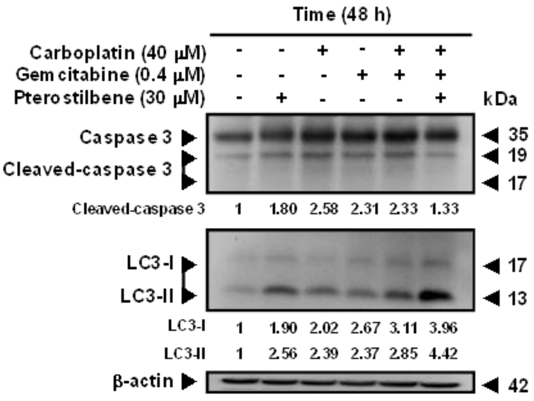

(C)

Figure S5. Effects of pterostilbene on autophagy of T24 and E7 cells treated with carboplatin and gemcitabine. (A) Pterostilbene enhanced autophagy of T24 cells treated with anti-cancer drugs. (B) Pterostilbene did not induce autophagy of E7 cells. After treatment for 48 h, the T24 and E7 cells were stained with acridine orange before flow cytometry. The percentages in the figure indicate the proportion of cells (upper two quadrants) with AVOs staining of at least three independent experiments. Data are presented as means ± SEMs. * denotes a significant difference compared to the group in the absence of pterostilbene, $P < 0.05$. The red color denotes the same controls shown in Fig. 3B and C, as they are the same set of experiments. (C) Protein expression of cleaved caspase 3 and LC3-II. After treatment for 48 h, total protein of T24 cells was subjected to Western blot analysis. Anti-caspase 3 and anti-LC3 antibodies served as probes. β-actin served as a loading control. The intensity of each protein expression band was quantified through densitometry normalization to that of β-actin, with the control level arbitrarily set to 1. Results are representative of three independent experiments.

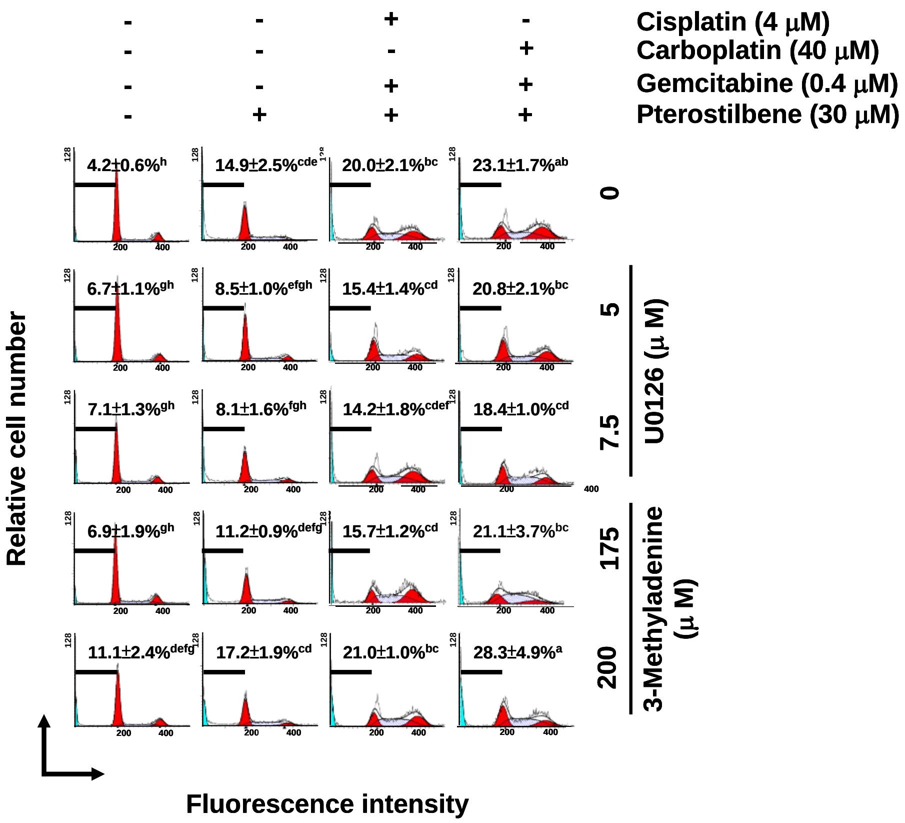

(A)

Time (h)	48			
Cisplatin (4 μM)	-	-	+	-
Carboplatin (40 μM)	-	-	-	+
Gemcitabine (0.4 μM)	-	-	+	+
Pterostilbene (30 μM)	-	+	+	+
	%			
Sub-G1	4.2±0.6^{h}	14.9±2.5^{cde}	20.0±2.1^{bc}	23.2±1.7^{ab}
G0/G1	65.1±1.8^{a}	50.2±1.3^{efgh}	40.6±0.5^{gh}i	34.1±5.2^{i}
S	14.5±0.6^{ab}	17.0±0.8^{a}	19.5±0.9^{a}	17.7±4.8^{a}
G2/M	16.1±1.1^{a}	17.9±0.9^{a}	20.2±1.6^{a}	25.1±0.8^{ab}

Time (h)	48							
U0126 (μM)	5				7.5			
Cisplatin (4 μM)	-	-	+	-	-	-	+	-
Carboplatin (40 μM)	-	-	-	+	-	-	-	+
Gemcitabine (0.4 μM)	-	-	+	+	-	-	+	+
Pterostilbene (30 μM)	-	+	+	+	-	+	+	+
	%							
Sub-G1	6.7±1.1^{gh}	8.5±1.0^{efgh}	15.4±1.4^{cd}	20.8±2.1^{bc}	7.1±1.3^{gh}	8.1±1.6^{fgh}	14.2±1.8^{cdef}	18.4±1.0^{cd}
G0/G1	61.6±5.1^{abc}	53.5±0.6^{def}	42.0±1.3^{fghi}	37.5±2.7^{i}	62.3±3.5^{ab}	53.6±0.7^{cdef}	41.2±4.1^{ghi}	36.5±3.8^{i}
S	16.4±1.0^{a}	20.9±1.8^{b}	22.5±1.7^{a}	20.9±1.7^{a}	15.7±1.3^{a}	20.7±1.7^{a}	23.3±3.6^{a}	22.2±1.9^{a}
G2/M	15.3±5.1^{a}	17.2±1.9^{a}	20.1±0.1^{a}	20.8±5.5^{a}	14.9±1.5^{a}	17.5±1.8^{a}	21.3±1.1^{a}	22.9±4.5^{a}

Time (h)	48							
3-MA (μM)	175				200			
Cisplatin (4 μM)	-	-	+	-	-	-	+	-
Carboplatin (40 μM)	-	-	-	+	-	-	-	+
Gemcitabine (0.4 μM)	-	-	+	+	-	-	+	+
Pterostilbene (30 μM)	-	+	+	+	-	+	+	+
	%							
Sub-G1	6.9±1.9^{gh}	11.2±0.9^{defg}	15.7±1.2^{cd}	21.1±3.7^{bc}	11.1±24^{defg}	17.2±1.9^{cd}	21.0±1.0^{bcf}	28.3±4.9^{a}
G0/G1	57.0±3.5^{bcd}	52.0±1.5^{defg}	38.8±3.6^{hi}	38.6±1.0^{hi}	53.9±6.5^{de}	45.7±1.5^{fghi}	43.0±1.3^{ghi}	34.4±8.3^{i}
S	20.0±5.2^{a}	19.7±2.8^{a}	21.6±1.7^{a}	17.6±1.6^{a}	18.7±4.0^{a}	22.9±4.1^{a}	19.2±0.3^{a}	18.1±3.1^{a}
G2/M	16.1±2.8^{a}	17.1±4.0^{a}	23.9±3.8^{a}	22.7±1.4^{a}	16.0±0.9^{a}	14.2±3.4^{a}	16.8±2.5^{a}	19.2±4.3^{a}

(B)

Figure S6. Effect of autophagy inhibitors on apoptosis of T24 cells. (A) Induction of apoptosis. The percentages in the figure indicate the proportion of apoptotic cells. (B) Quantification of cell cycle profiles and the percentages of cells at the sub-G1 phase, representing apoptotic cells. T24 cells were either pre-treated with U0126 for 2 h prior to the addition of the indicated concentration of compounds for 48 h, or co-treated with 3-MA and the indicated concentration of compounds for 48

h. After treatment, the cells were stained with propidium iodide before flow cytometry. Data of at least three independent experiments were quantified and are presented as means ± SEMs. Means in each cell cycle with different superscript letters are significantly different, $P < 0.05$..

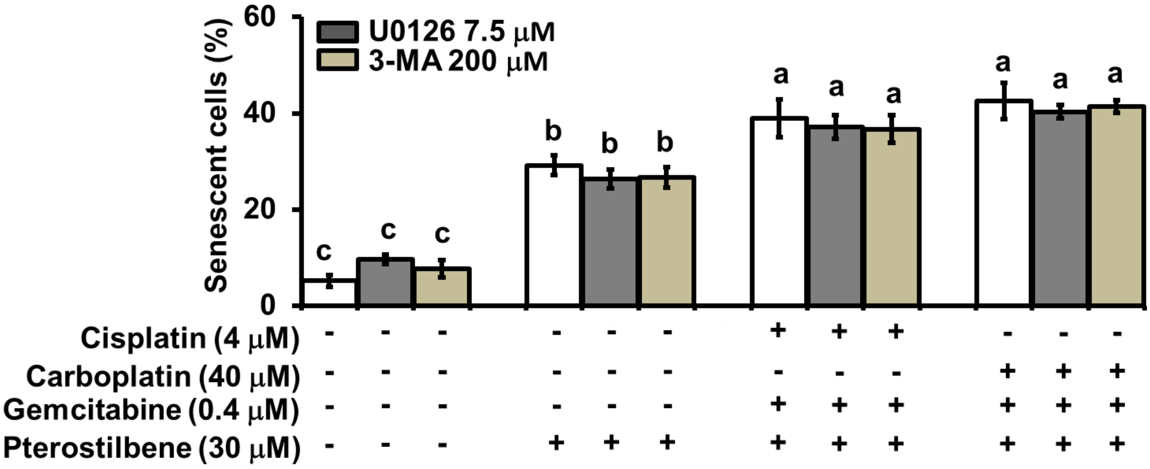

Figure S7. Effect of autophagy inhibitors on senescence of T24 cells. T24 cells were either pre-treated with U0126 for 2 h prior to the addition of the indicated concentration of compounds for 48 h, or co-treated with 3-MA and the indicated concentration of compounds for 48 h. After treatment, the activity of senescence-associated β-galactosidase was determined by the hydrolysis of X-Gal to yield a blue-colored product. The number of blue-colored cells of at least three independent experiments was quantified. At least 100 cells were counted for each experiment. Data of at least three independent experiments were quantified and are presented as means ± SEMs. Means with different superscript letters are significantly different, $P < 0.05$.

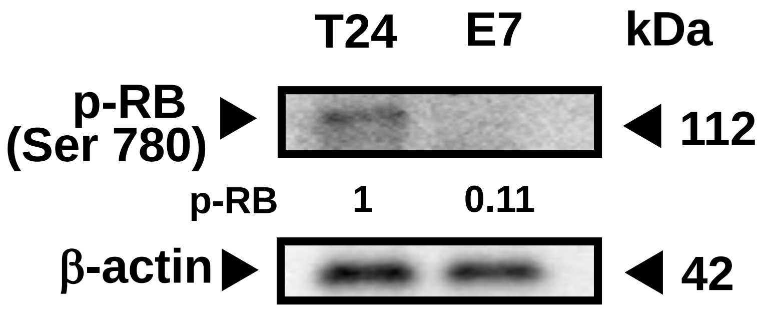

Figure S8. The phosphorylation of RB protein in T24 and E7 cells. Total protein in the cells was subjected to Western blot analysis. Anti-p-RB antibody served as a probe. β-actin served as a loading control. Results are representative of three independent experiments.

© 2020 by the authors. Licensee MDPI, Basel, Switzerland. This article is an open access article distributed under the terms and conditions of the Creative Commons Attribution (CC BY) license (http://creativecommons.org/licenses/by/4.0/).
