# Supplementary material for: Pterostilbene Sensitizes Cisplatin-Resistant Human Bladder Cancer Cells with Oncogenic HRAS
Source: Cancers (Basel). 2020 Oct 6;12(10):2869. doi: 10.3390/cancers12102869 (PMC7650649; doi:10.3390/cancers12102869)

Fig. 3D Cis+Gem+PS_Caspase3, cleaved-caspase3

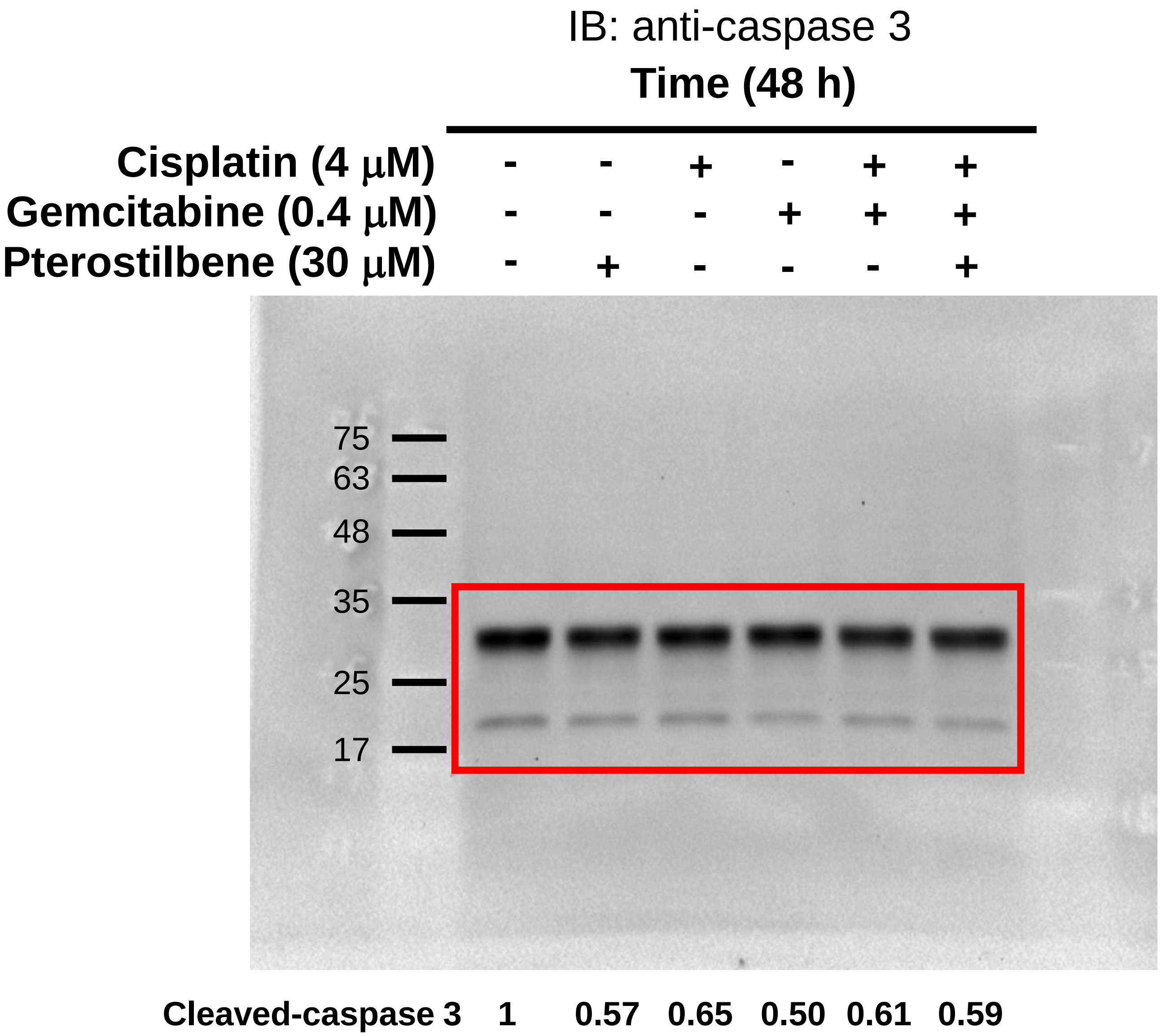

Fig. 3D Cis+Gem+PS_LC3-I, LC3-II

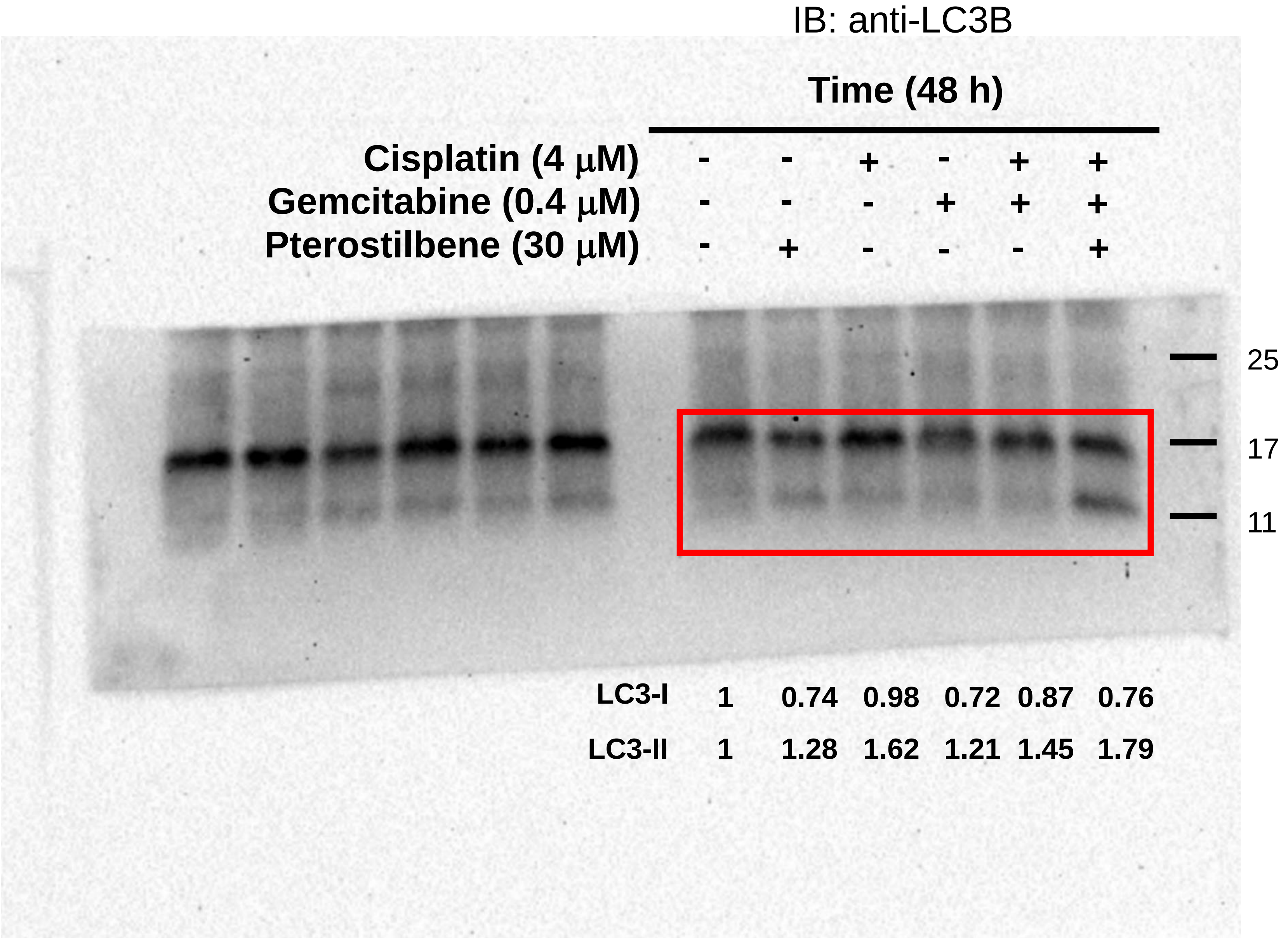

Fig. 3D Cis+Gem+PS_beta actin

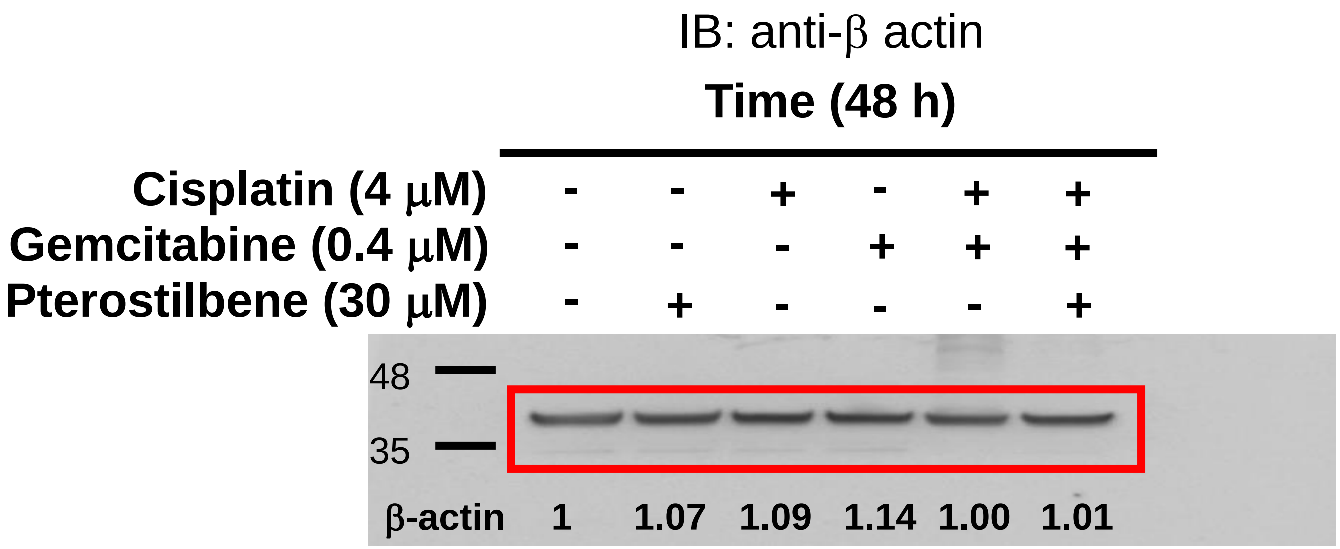

Fig. S5C Car+Gem+PS_Caspase3, cleaved-caspase3

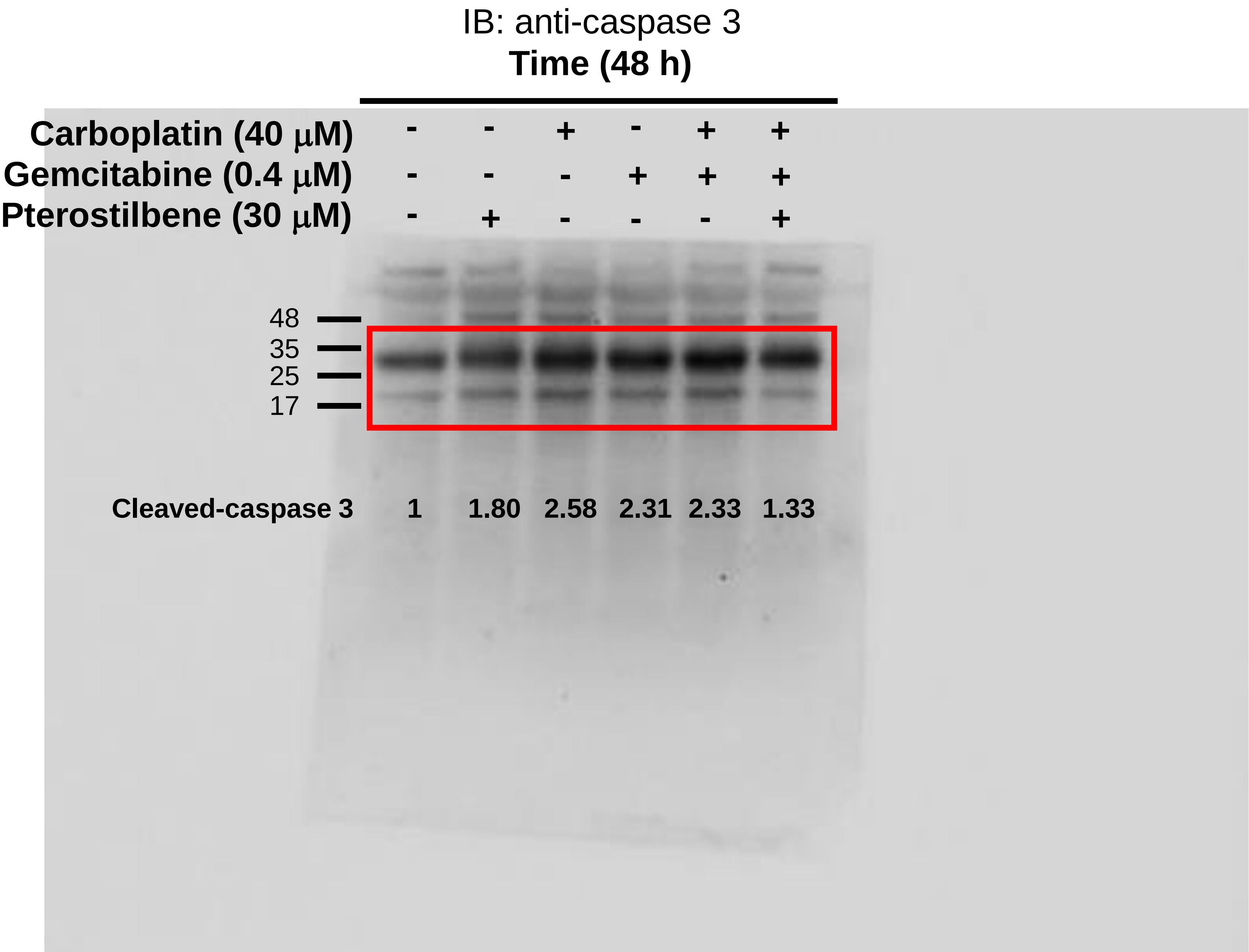

Fig. S5C Car+Gem+PS_LC3-I, LC3-II

IB: anti-LC3B

Time (48 h)

**Carboplatin (40 μM)**	-	-	+	-	+	+
**Gemcitabine (0.4 μM)**	-	-	-	+	+	+
**Pterostilbene (30 μM)**	-	+	-	-	-	+

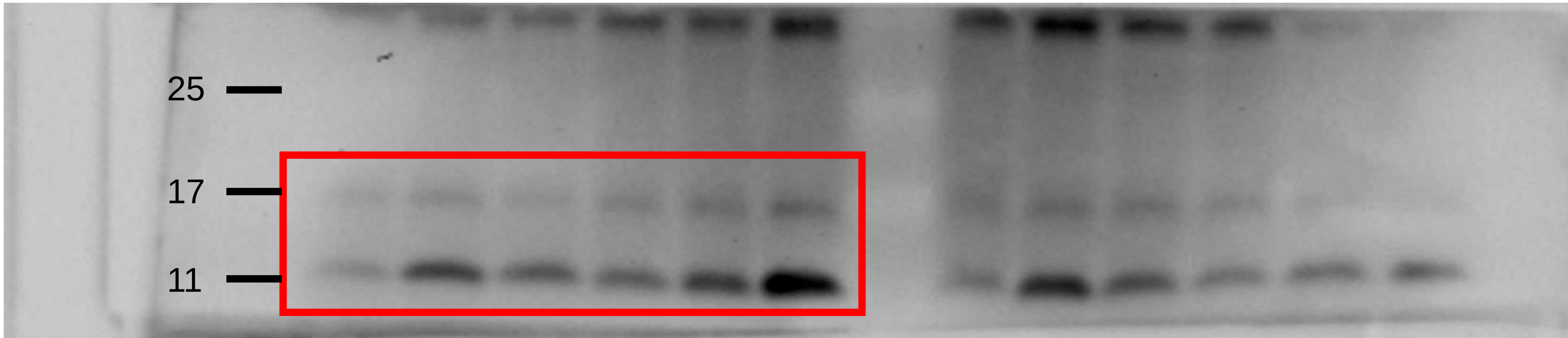

**LC3-I**	1	1.90	2.02	2.67	3.11	3.96
**LC3-II**	1	2.56	2.39	2.37	2.85	4.42

Fig. S5C Car+Gem+PS_beta actin

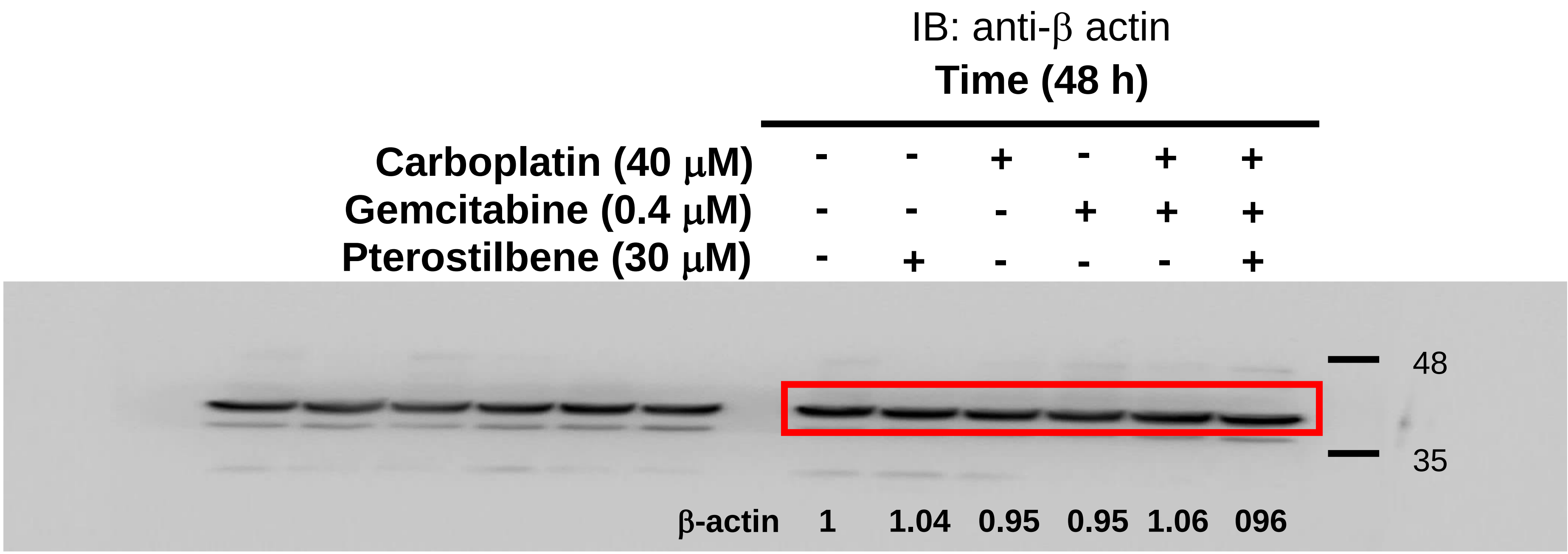

Fig. 4A Cis+Gem+PS_Atg12-Atg5, Atg5

IB: anti-Atg5

Time (48 h)

**Cisplatin (4 μM)**	-	-	+	-	+	+
**Gemcitabine (0.4 μM)**	-	-	-	+	+	+
**Pterostilbene (30 μM)**	-	+	-	-	-	+

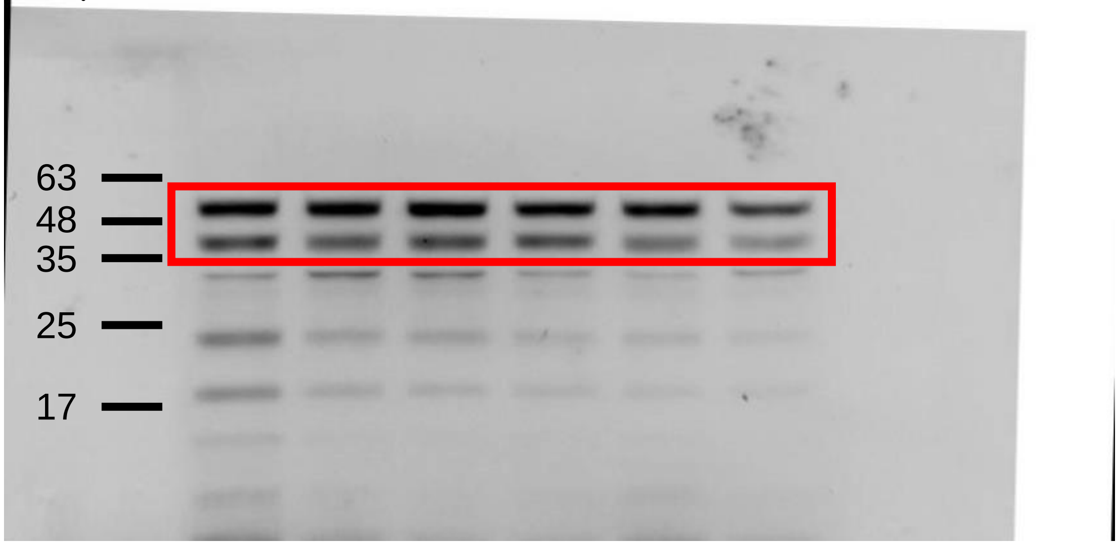

**Atg12-Atg5**	**1**	**1.06**	**1.23**	**1.06**	**1.12**	**0.72**
**Atg5**	**1**	**0.96**	**1.19**	**1.07**	**0.89**	**0.56**

Fig. 4A Cis+Gem+PS_beta actin (Atg12-Atg5, Atg5)

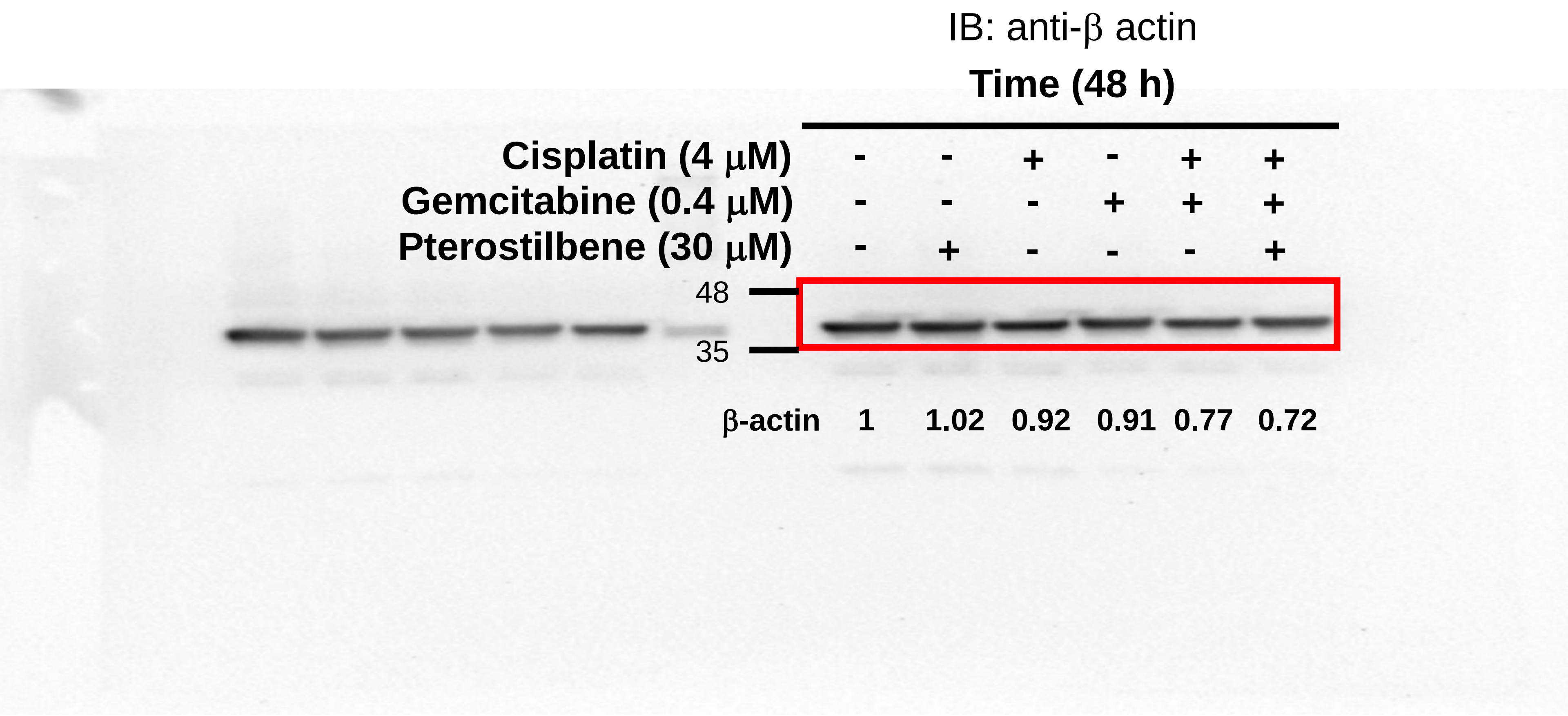

Fig. 4A Cis+Gem+PS_Class III PI3K

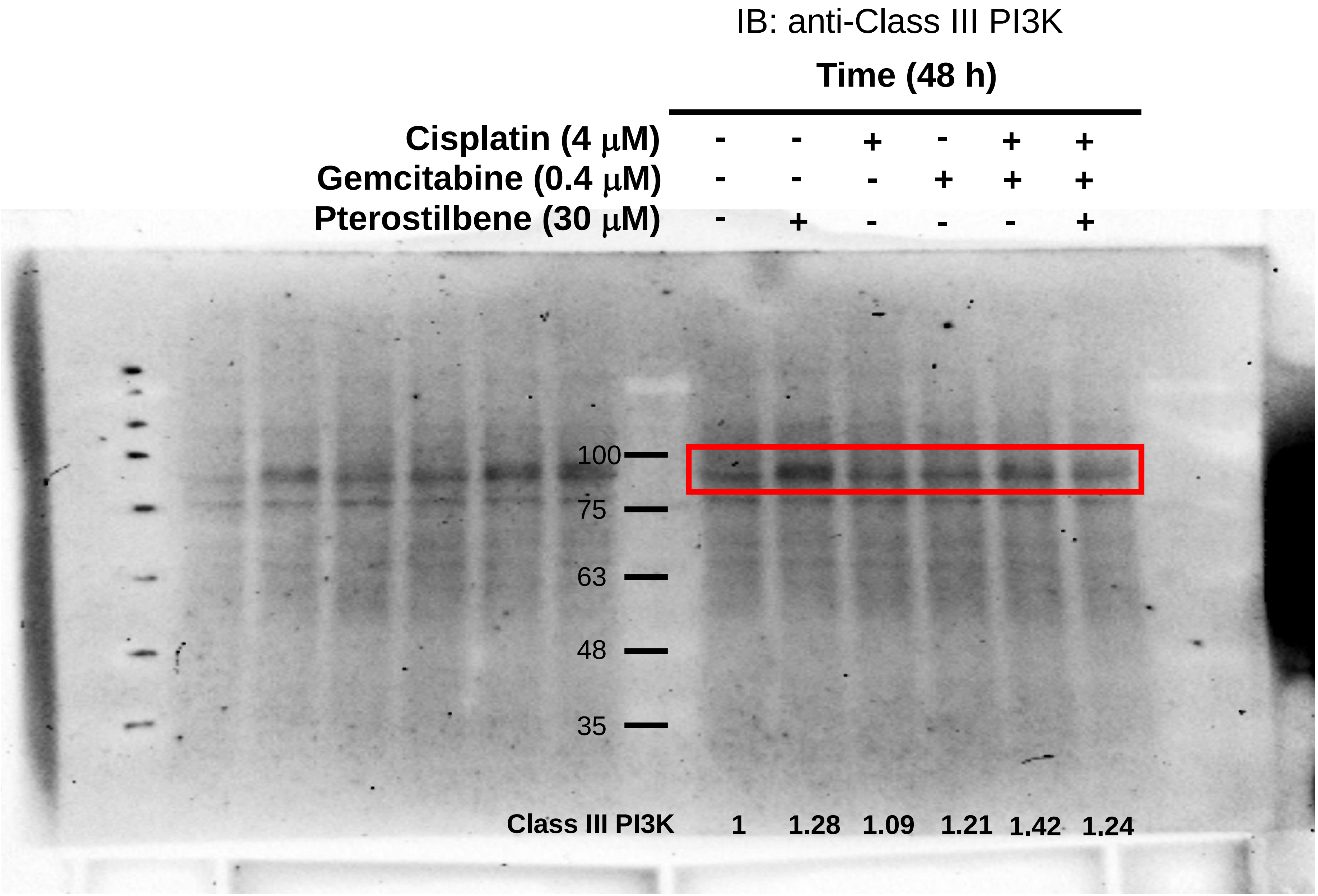

Fig. 4A Cis+Gem+PS_beta actin (Class III PI3K)

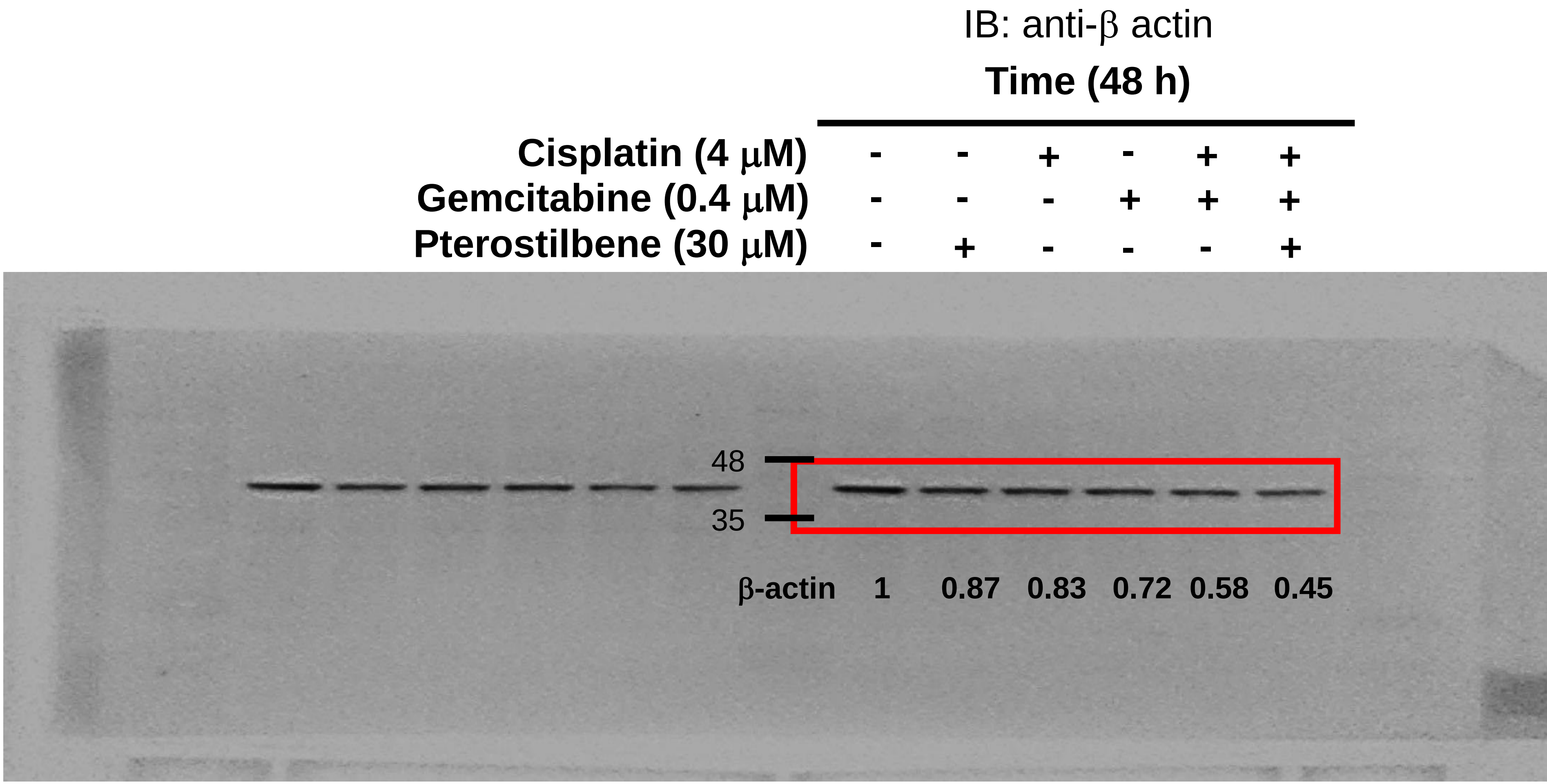

Fig. 4A Car+Gem+PS_Atg12-Atg5, Atg5

IB: anti-Atg5

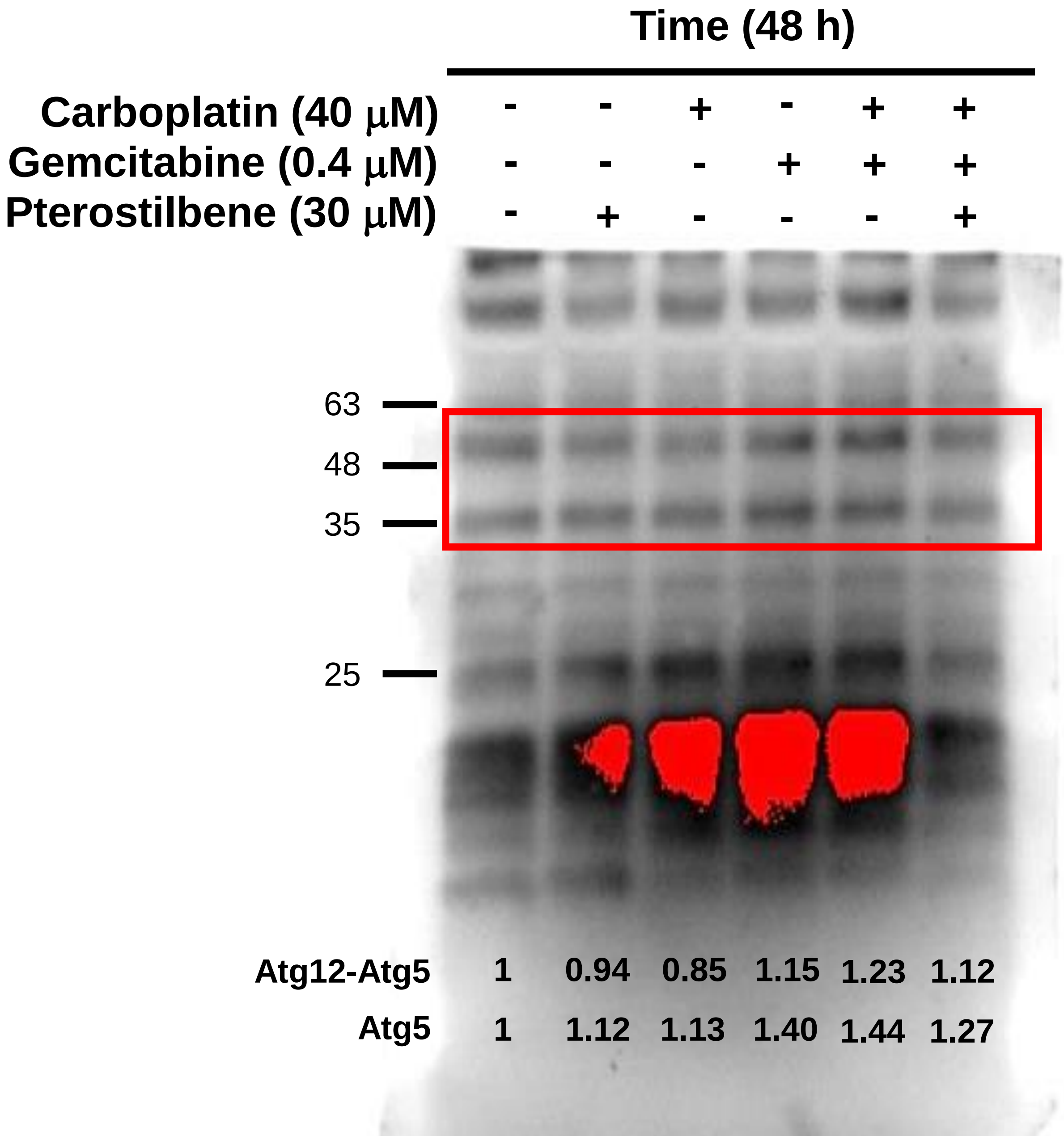

Fig. 4A Car+Gem+PS_beta actin (Atg12-Atg5, Atg5)

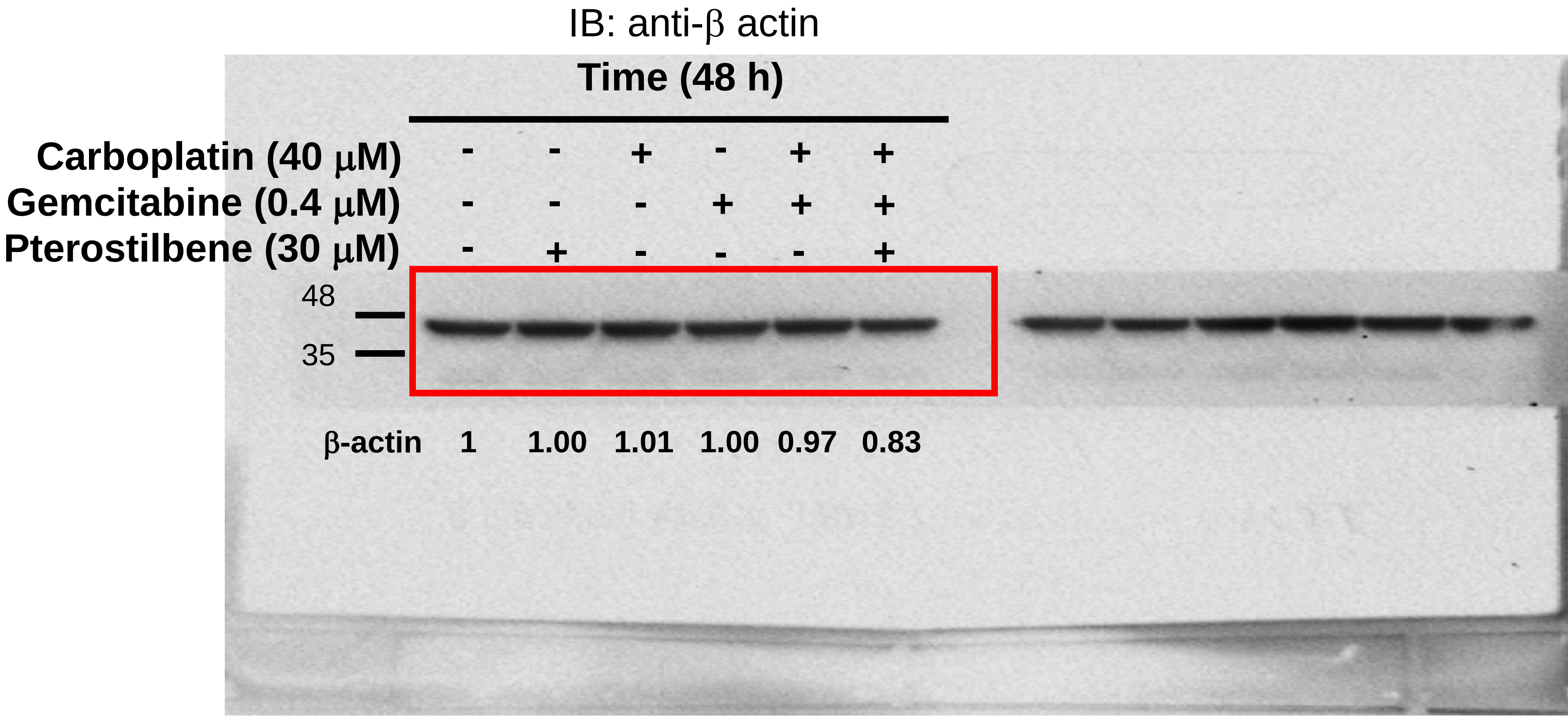

Fig. 4A Car+Gem+PS_Class III PI3K

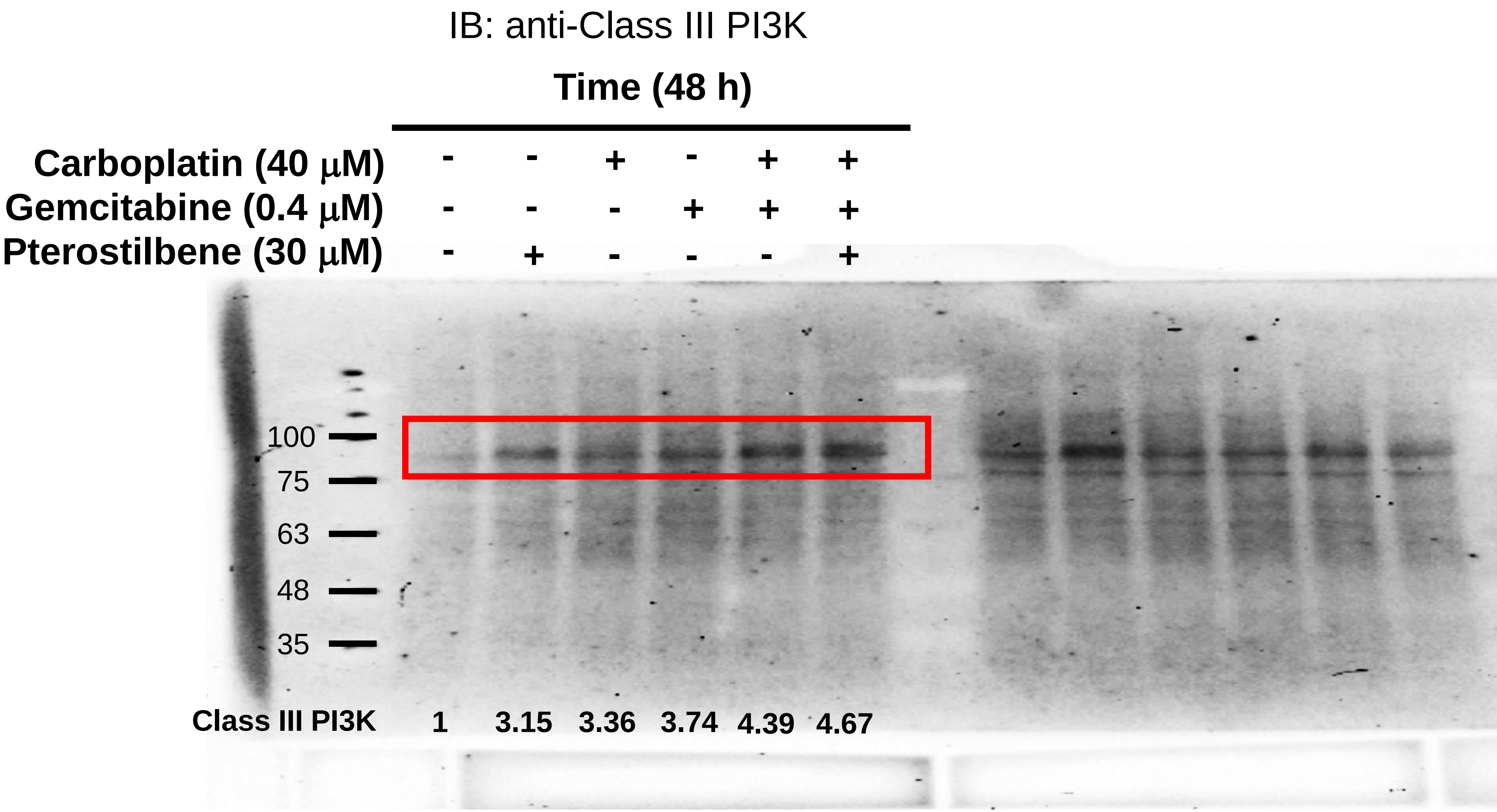

Fig. 4A Car+Gem+PS_beta actin (Class III PI3K)

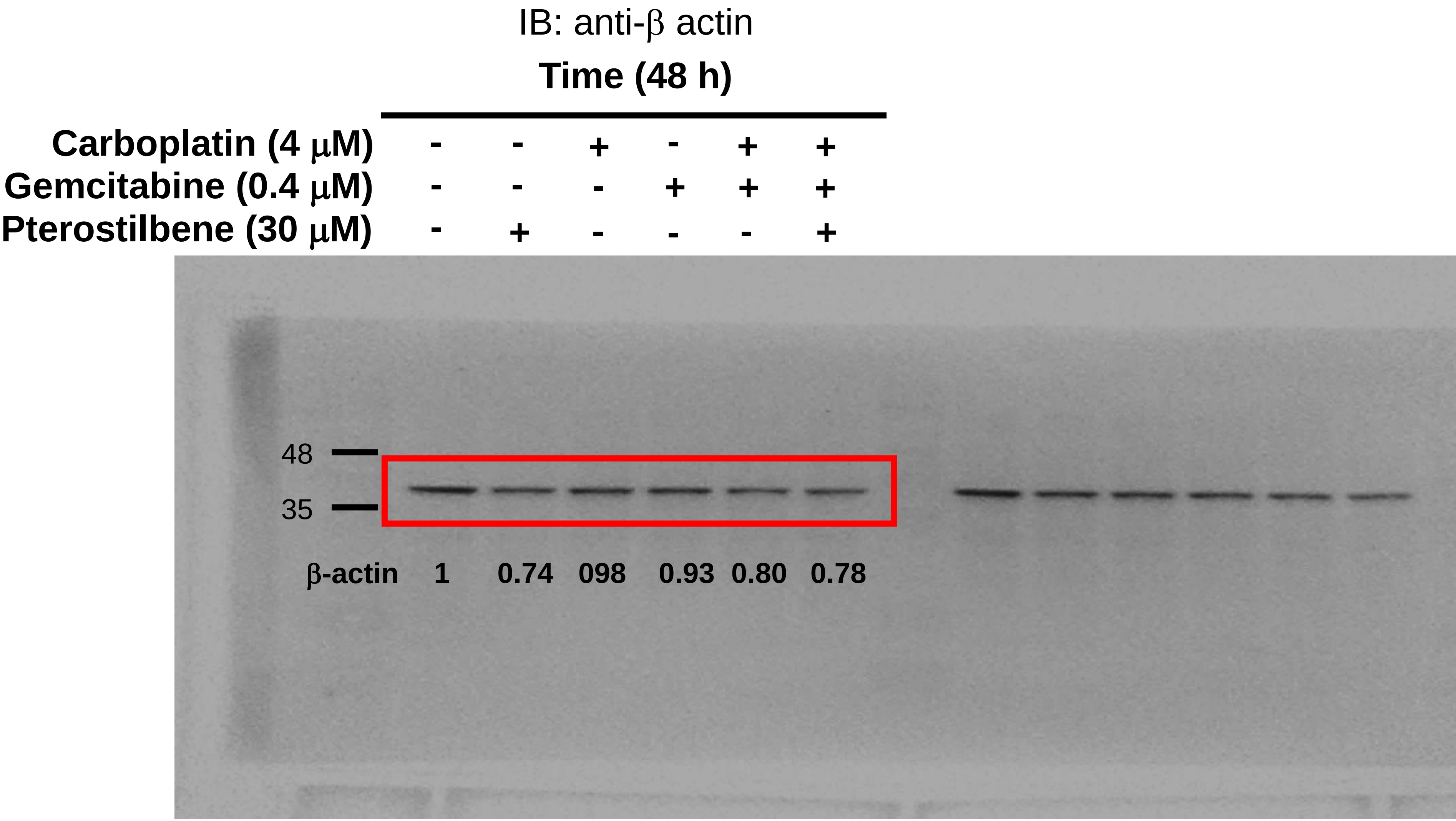

Fig. 4B Cis+Gem+PS_p-Class I PI3K

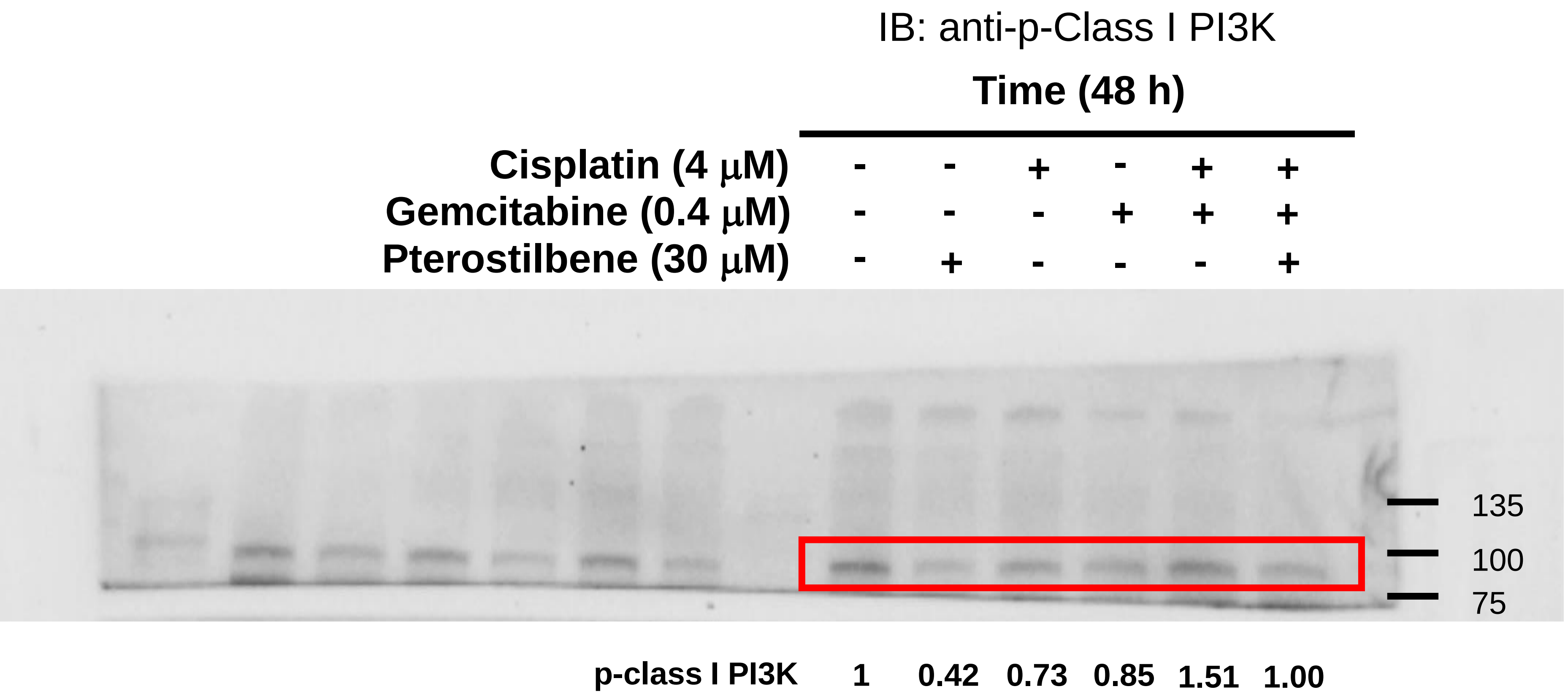

Fig. 4B Cis+Gem+PS_p-mTOR

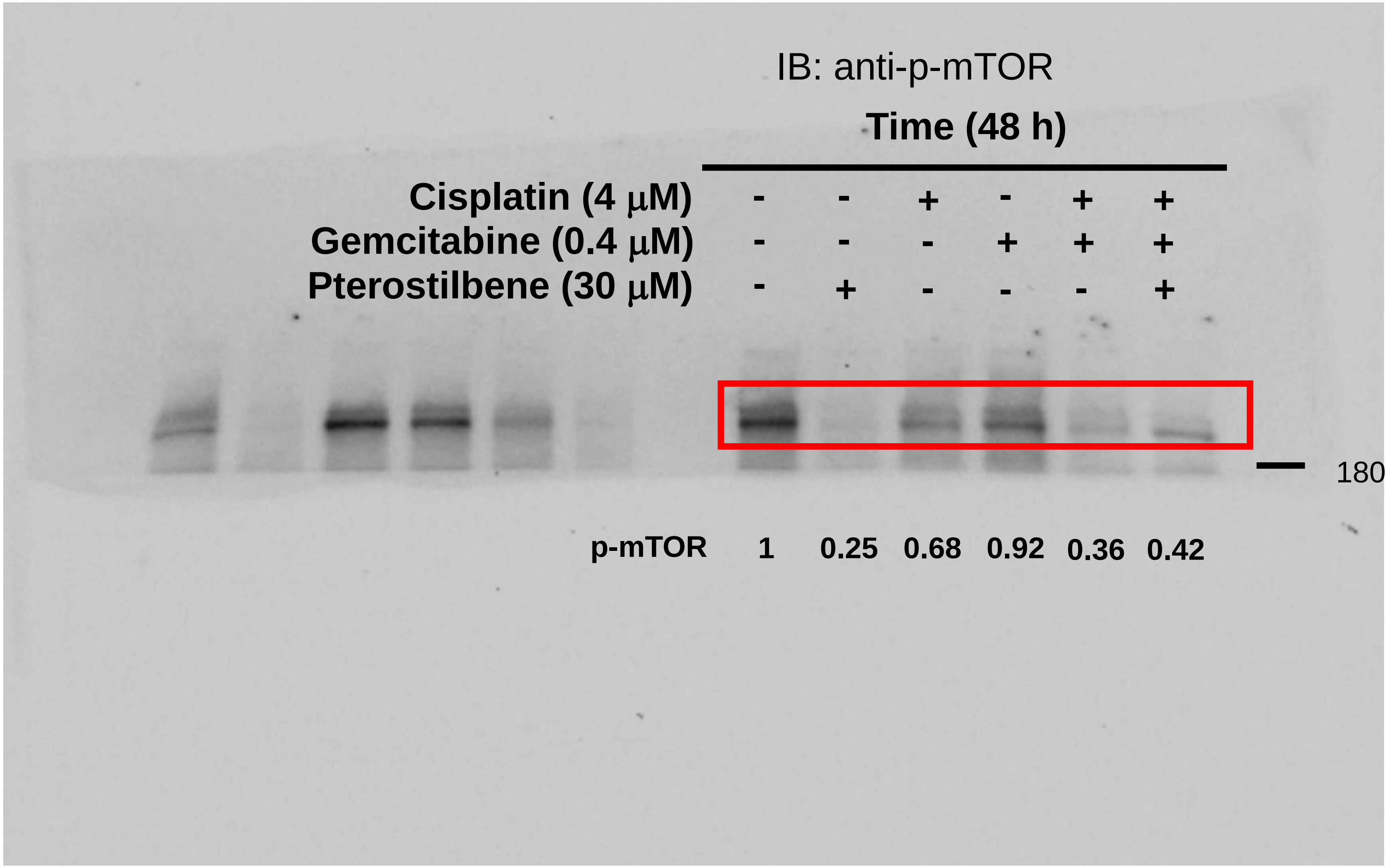

Fig. 4B Cis+Gem+PS_p-p70S6K

IB: anti-p-p70S6K

Time (48 h)

**Cisplatin (4 μM)**	-	-	+	-	+	+
**Gemcitabine (0.4 μM)**	-	-	-	+	+	+
**Pterostilbene (30 μM)**	-	+	-	-	-	+

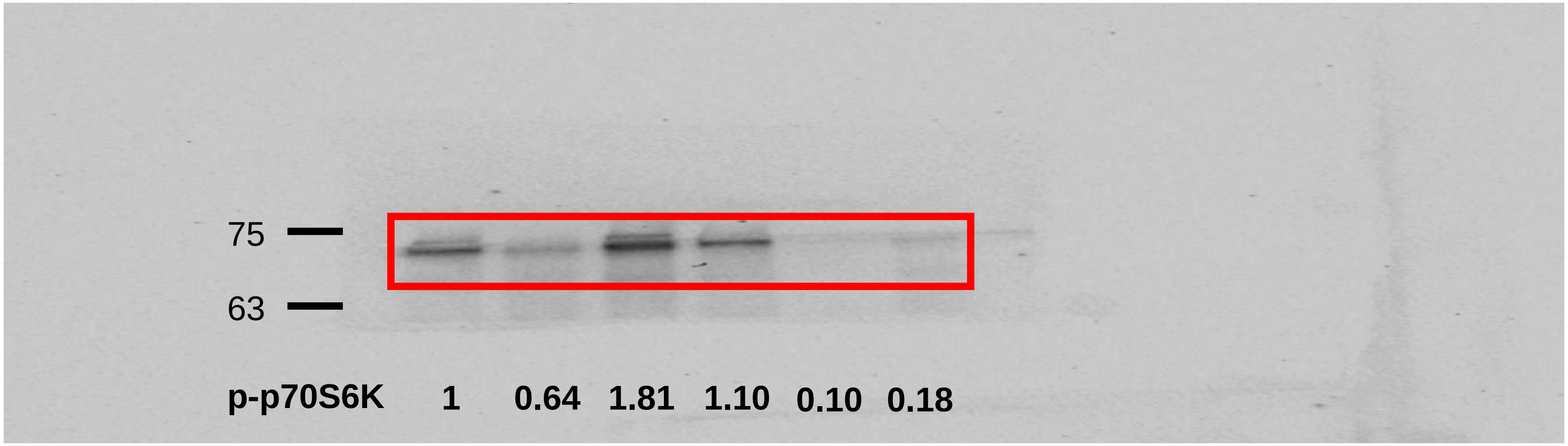

Fig. 4B Cis+Gem+PS_beta actin (p-Class I PI3K, p-mTOR, p-70S6K)

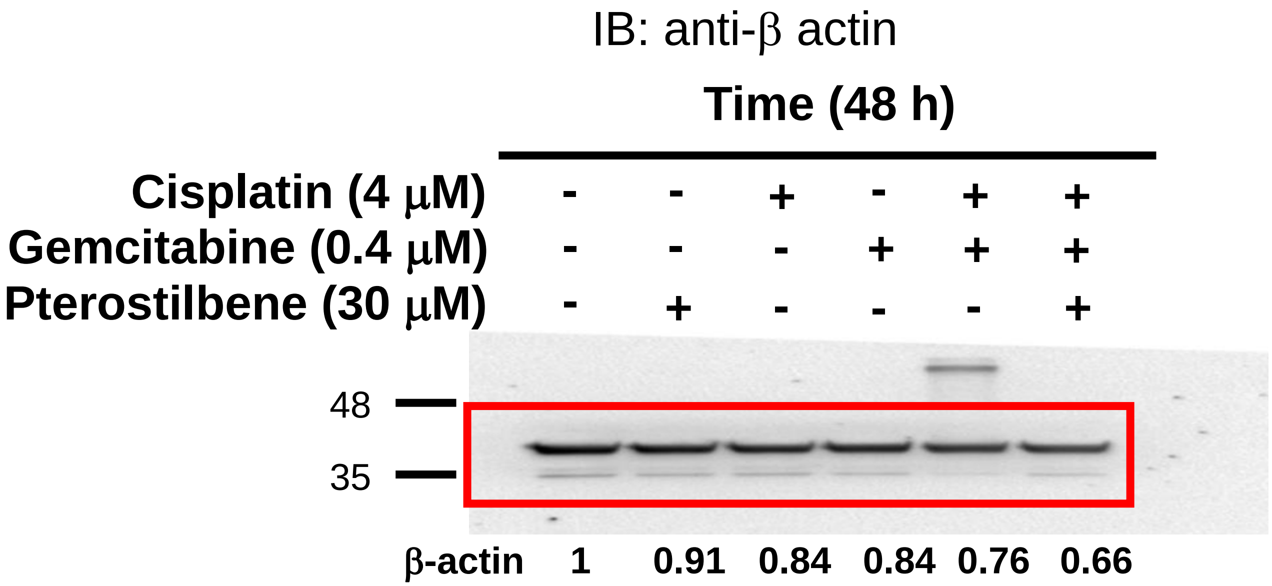

Fig. 4B Cis+Gem+PS_p-MEK

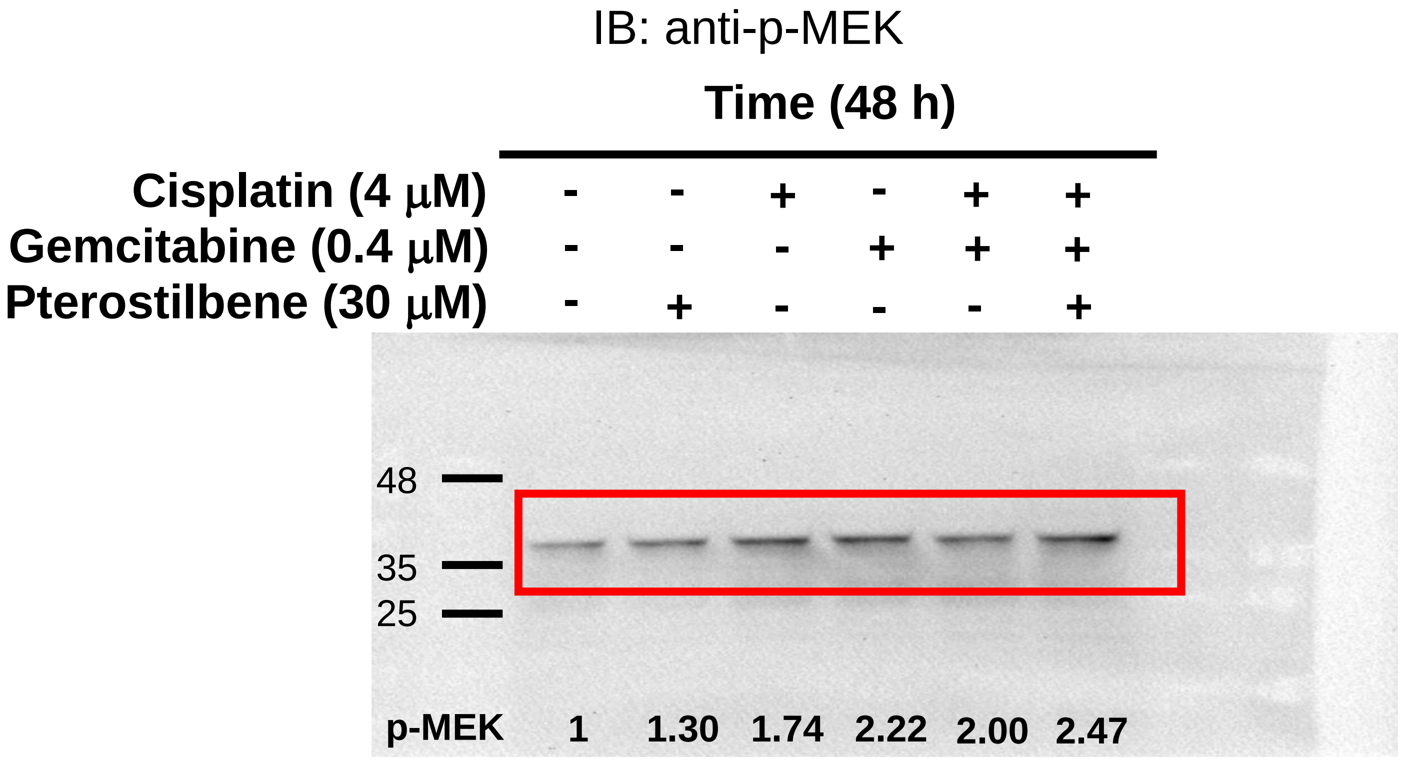

Fig. 4B Cis+Gem+PS_p-ERK

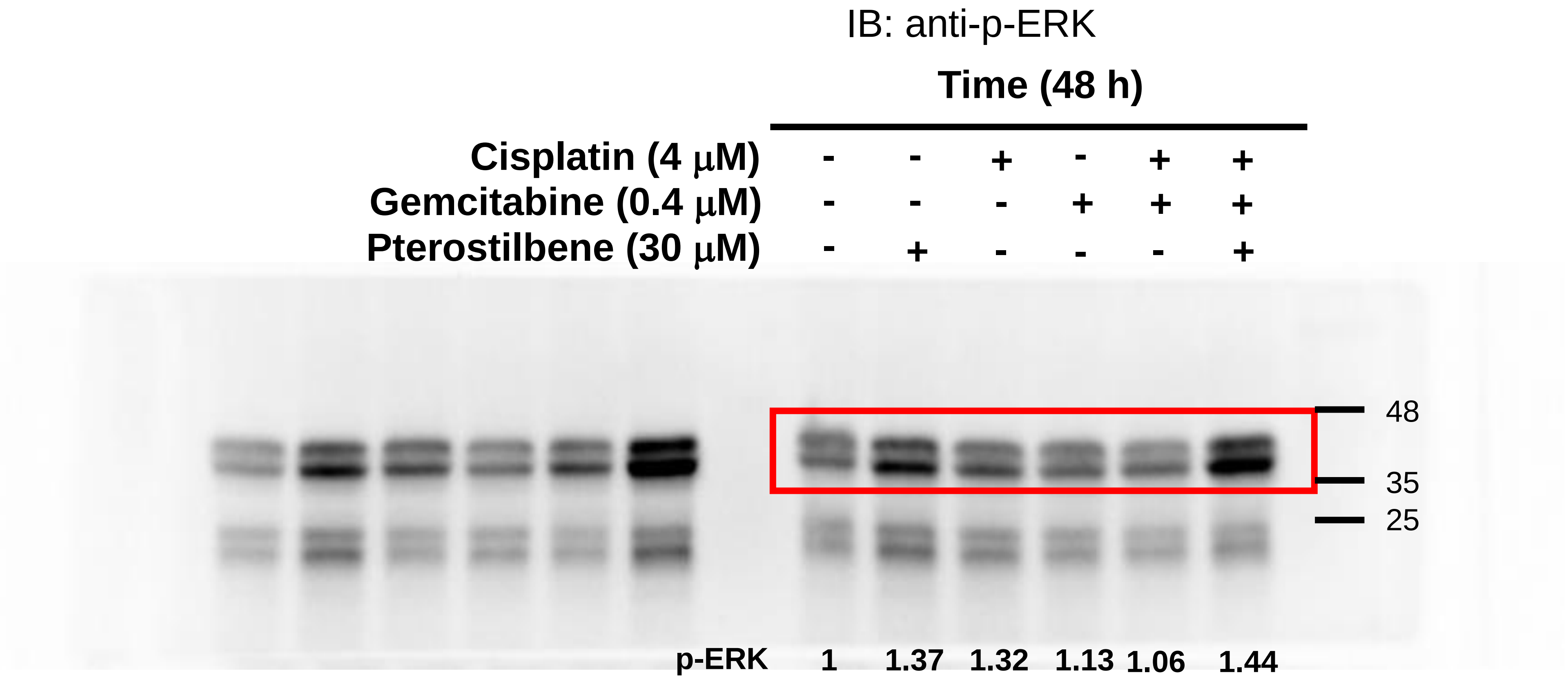

Fig. 4B Cis+Gem+PS_beta actin (p-MEK, p-ERK)

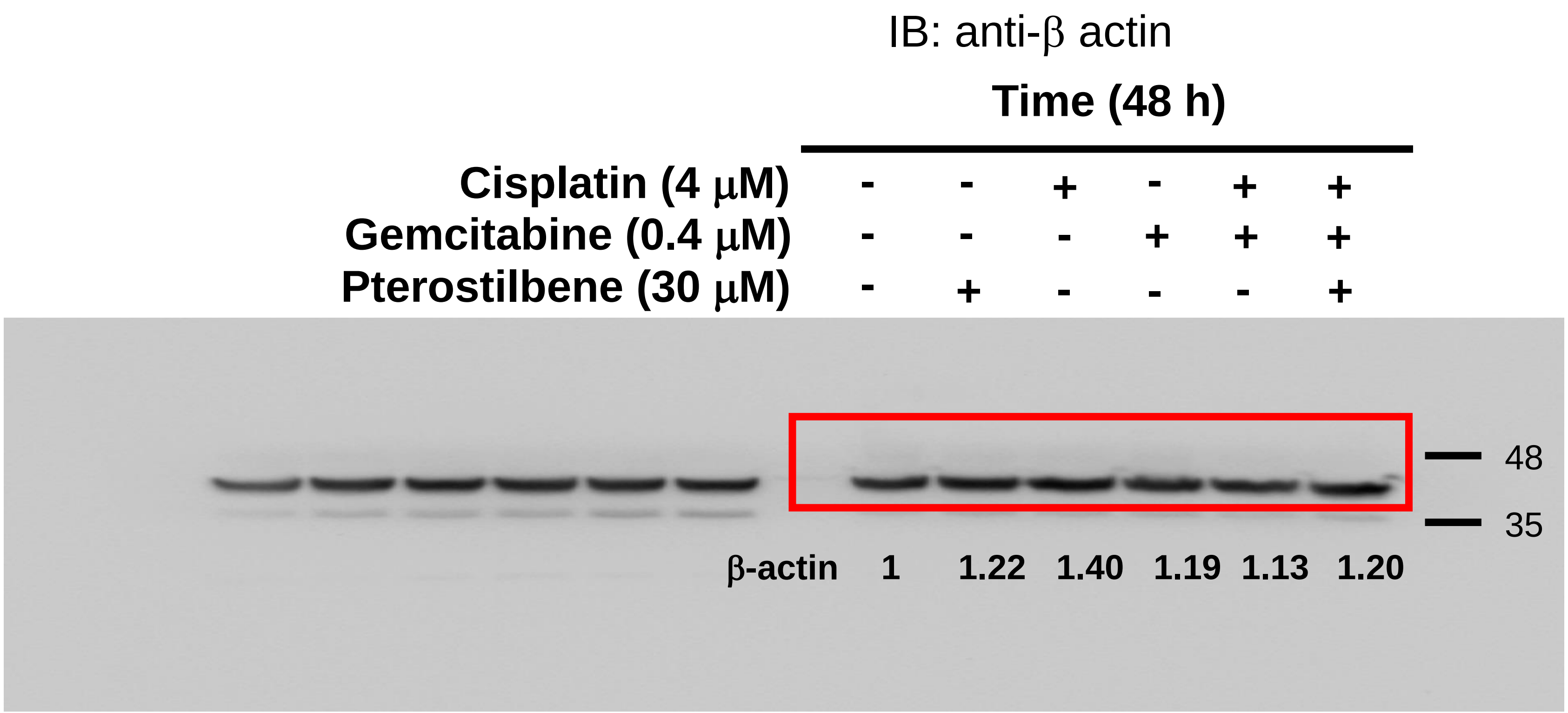

Fig. 4B Car+Gem+PS_p-Class I PI3K

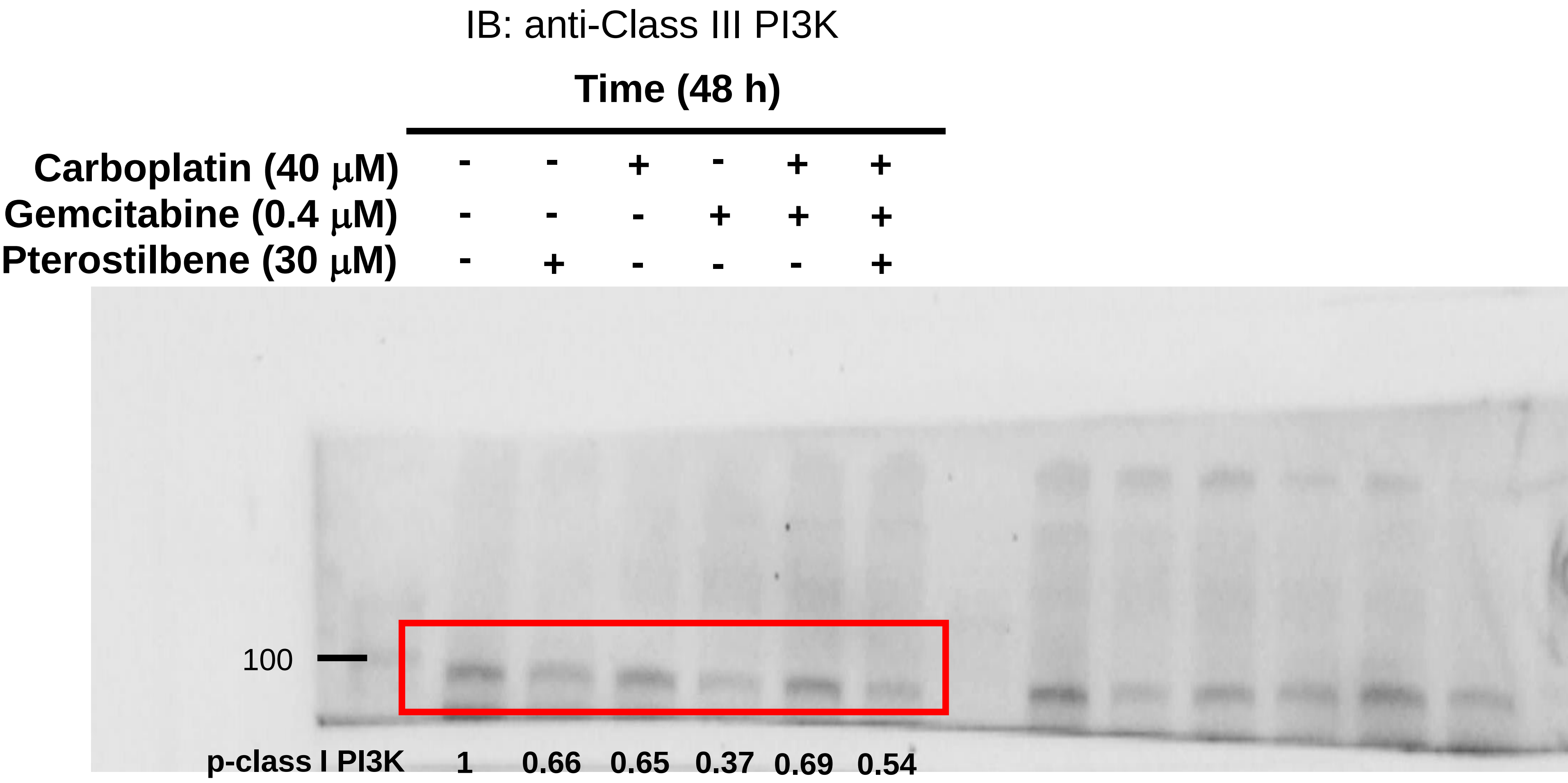

Fig. 4B Car+Gem+PS_p-mTOR

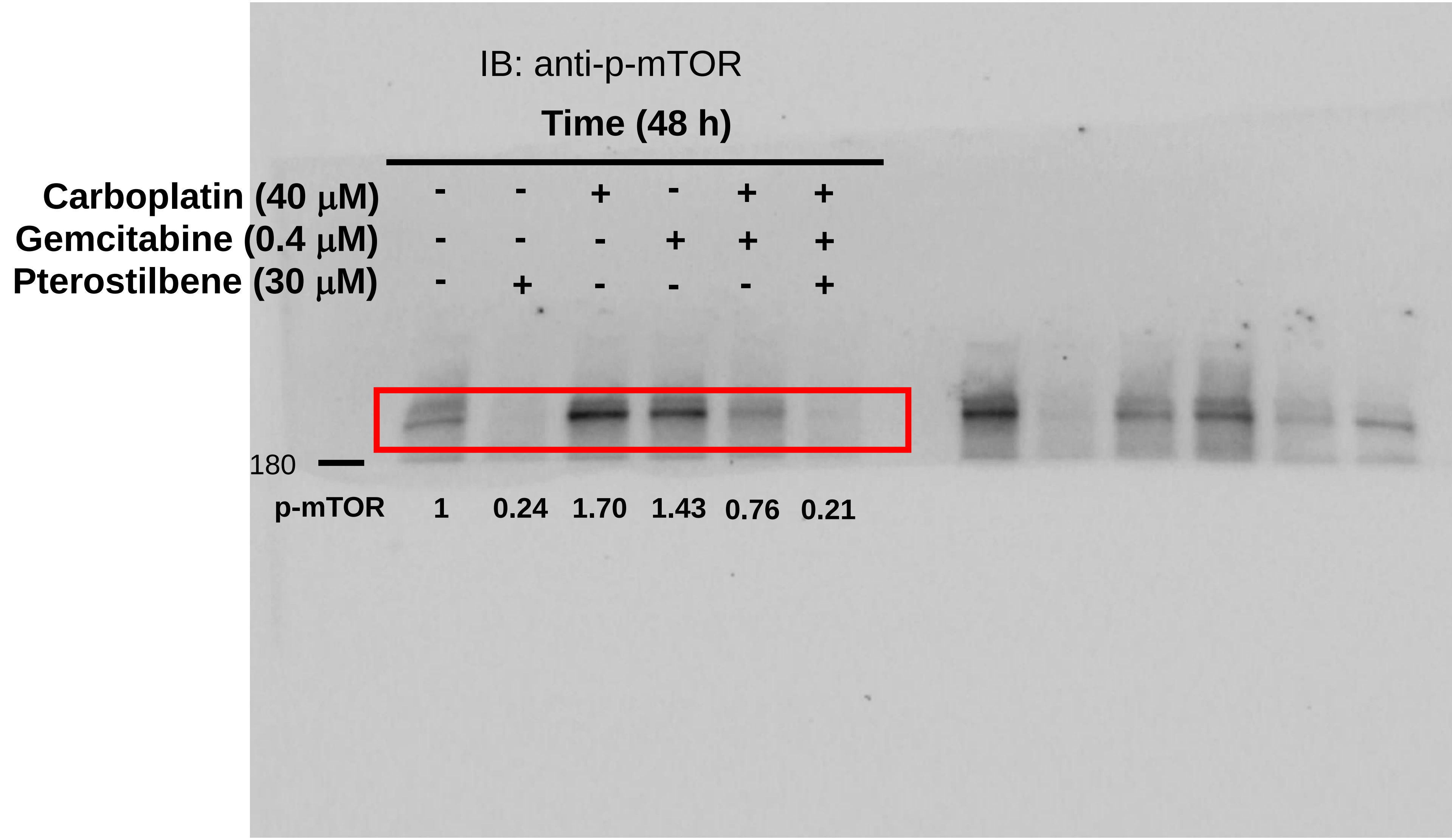

Fig. 4B Car+Gem+PS_p-p70S6K

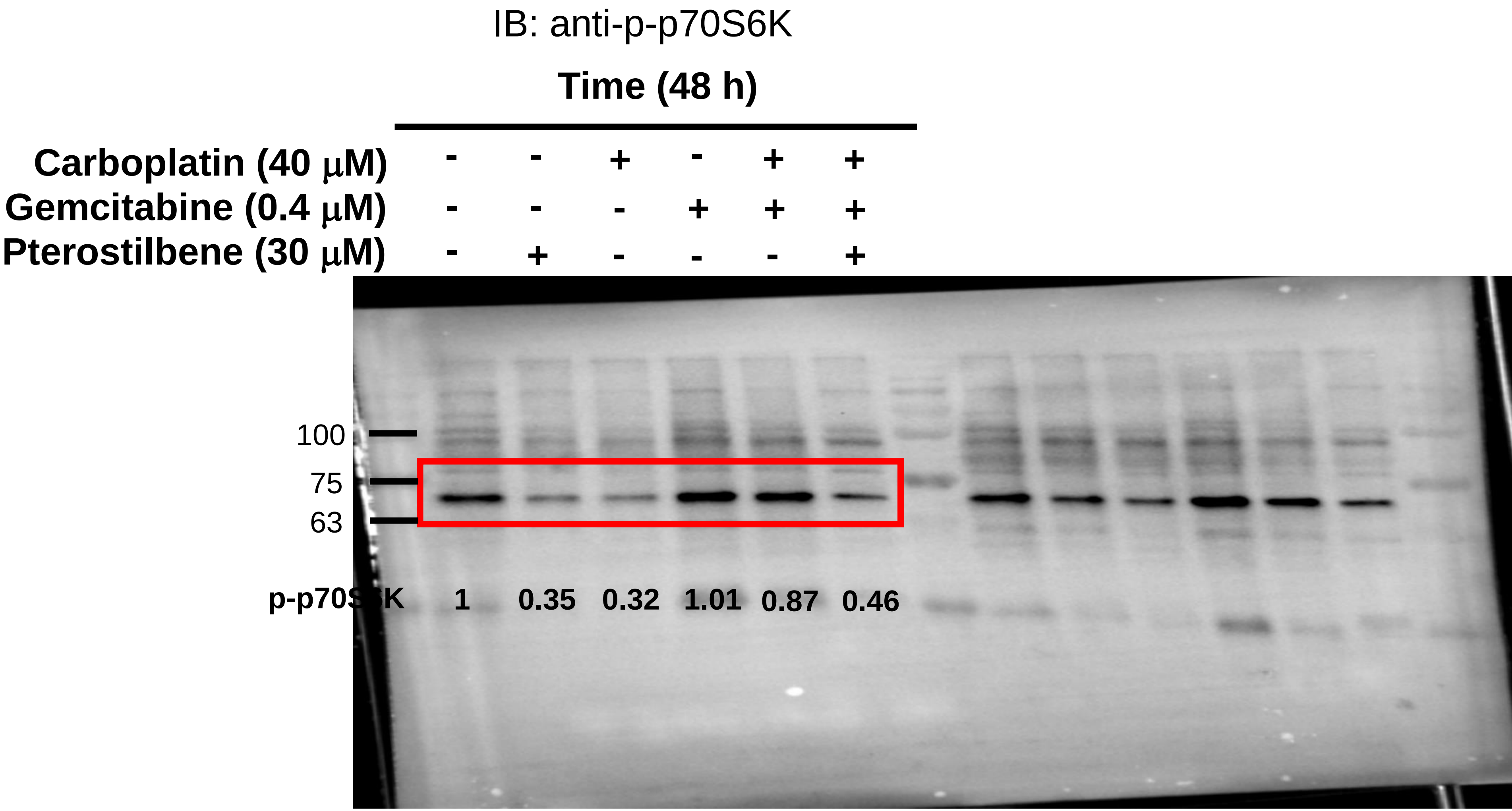

Fig. 4B Car+Gem+PS_beta actin (p-Class I PI3K, p-mTOR, p-70S6K)

IB: anti-β actin

Time (48 h)

**Carboplatin (40 μM)**	-	-	+	-	+	+
**Gemcitabine (0.4 μM)**	-	-	-	+	+	+
**Pterostilbene (30 μM)**	-	+	-	-	-	+

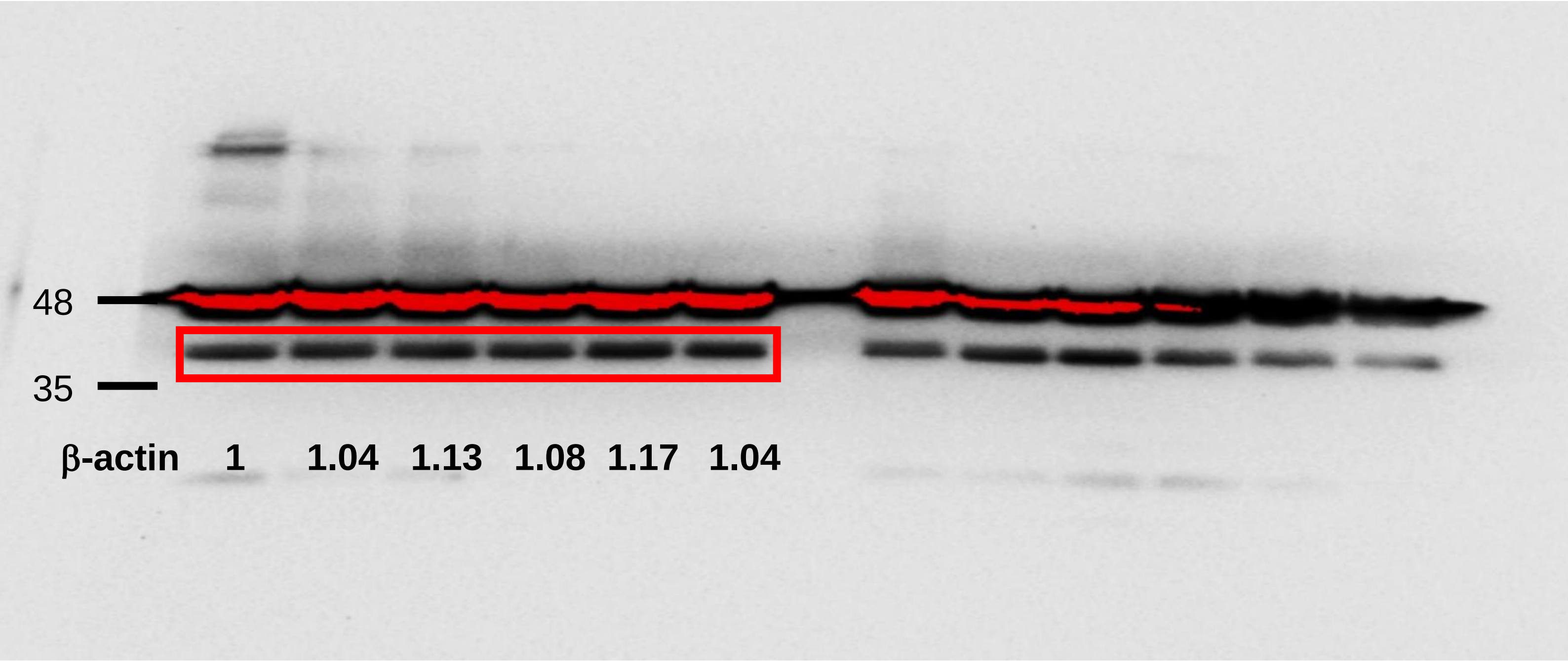

Fig. 4B Car+Gem+PS_p-MEK

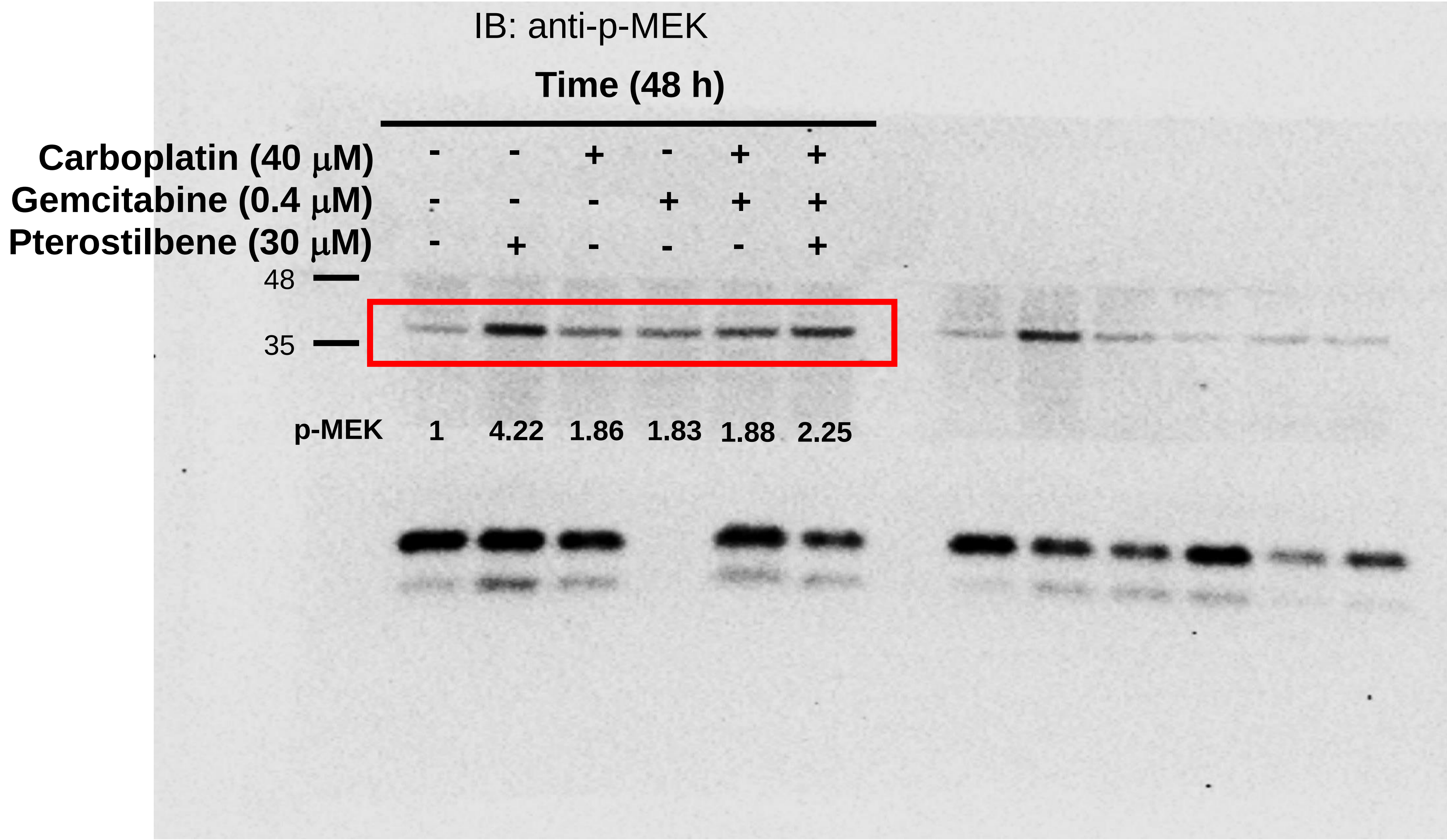

Fig. 4B Car+Gem+PS_p-ERK

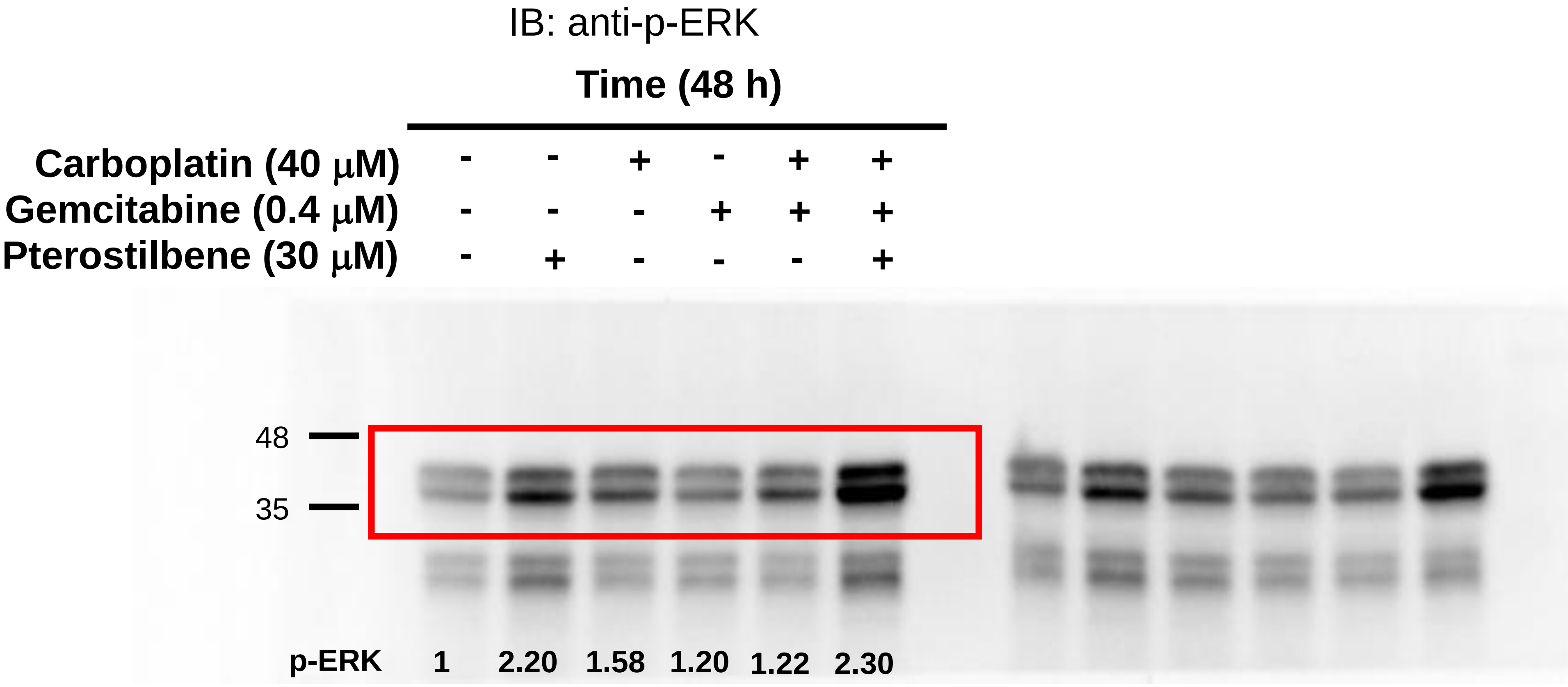

Fig. 4B Car+Gem+PS_beta actin (p-MEK, p-ERK)

IB: anti-β actin

Time (48 h)

**Carboplatin (40 μM)**	-	-	+	-	+	+
**Gemcitabine (0.4 μM)**	-	-	-	+	+	+
**Pterostilbene (30 μM)**	-	+	-	-	-	+

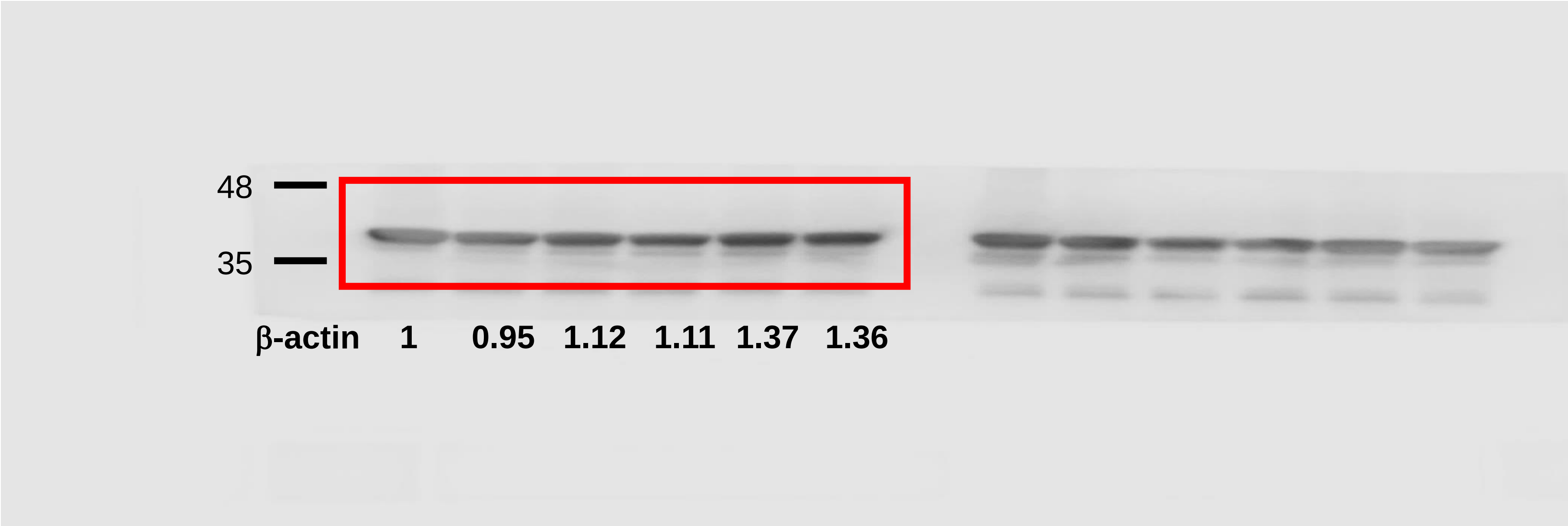

Fig. 5E Cis+Gem+PS_Pan-Ras

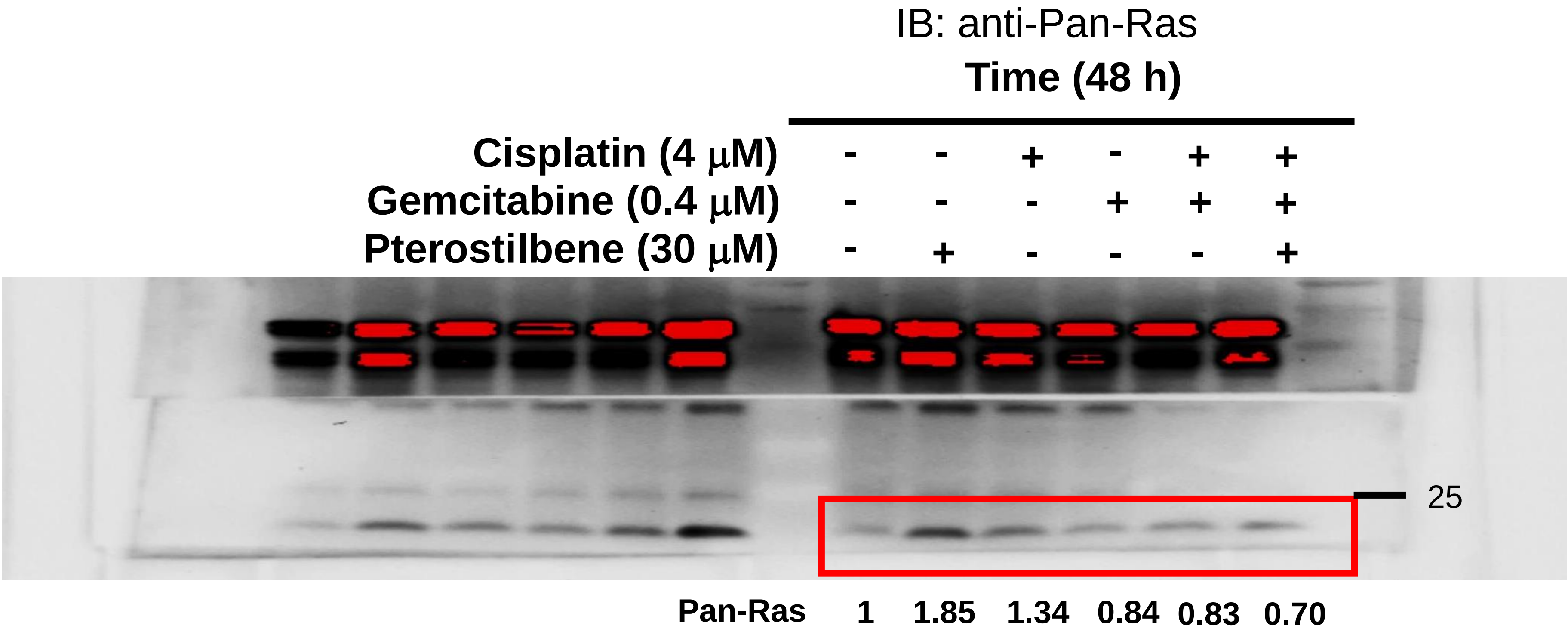

Fig. 5E Cis+Gem+PS_p-RB

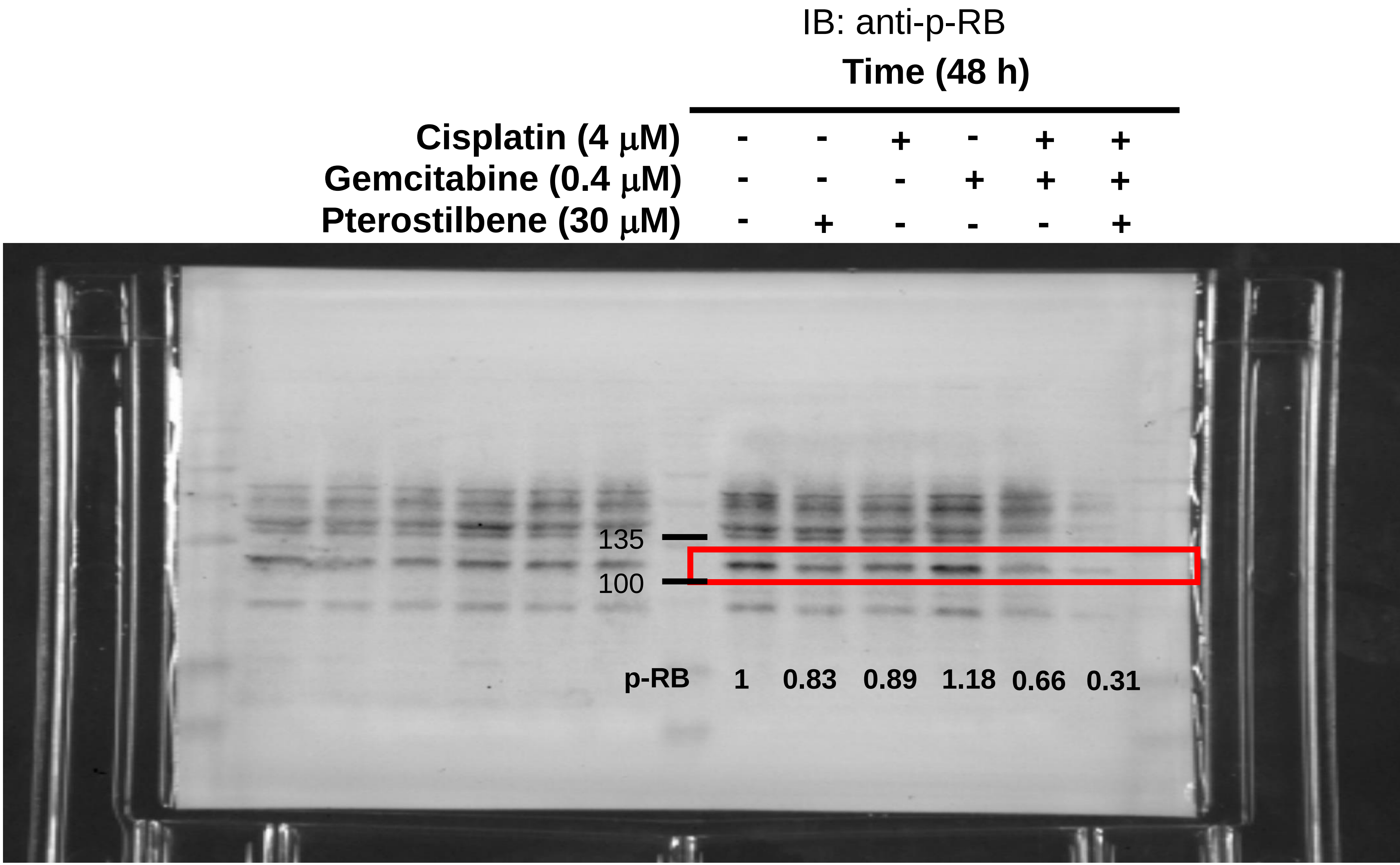

Fig. 5E Cis+Gem+PS_beta actin (Pan-Ras, p-RB)

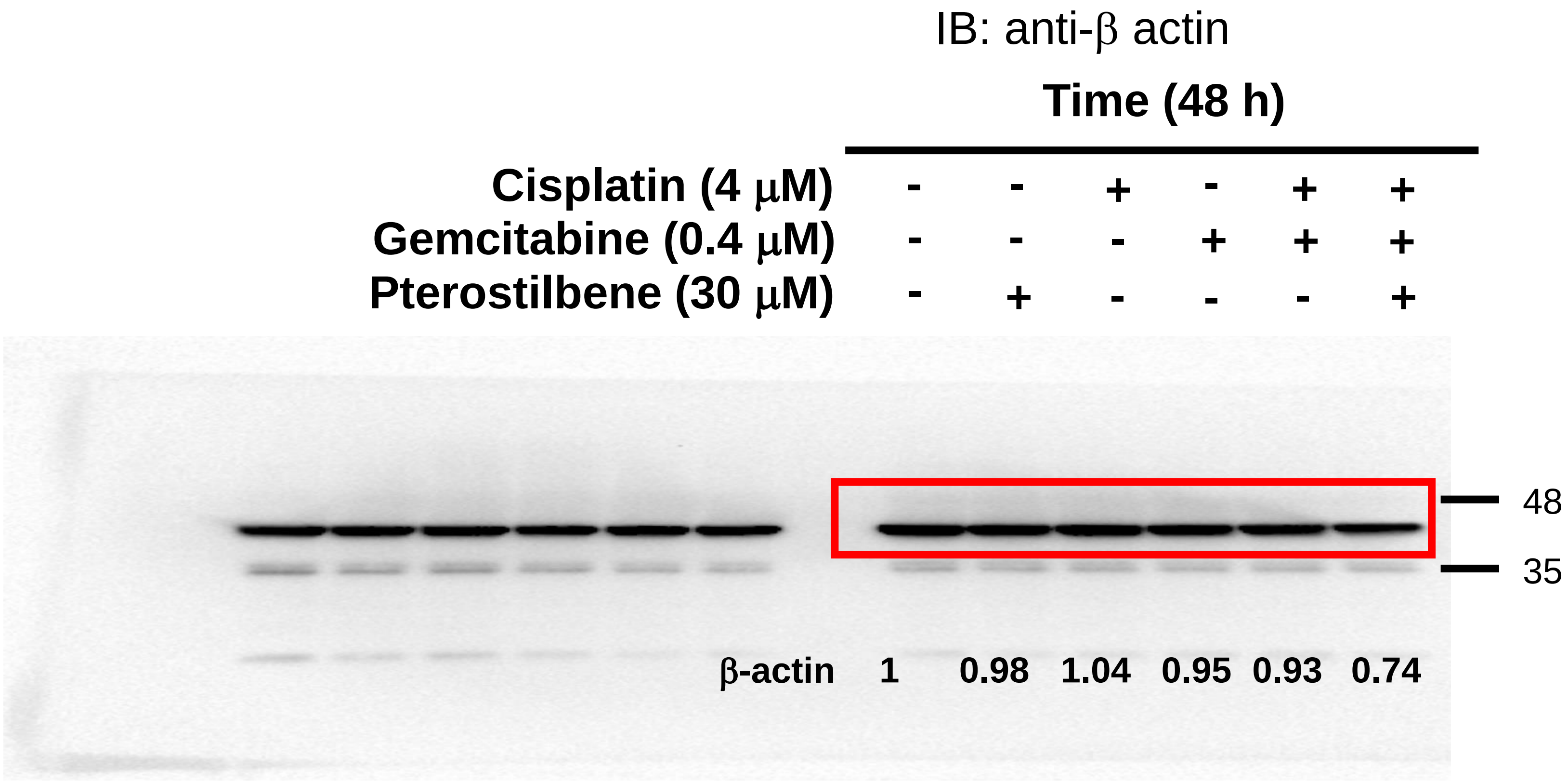

Fig. 5E Cis+Gem+PS_p-Aurora A

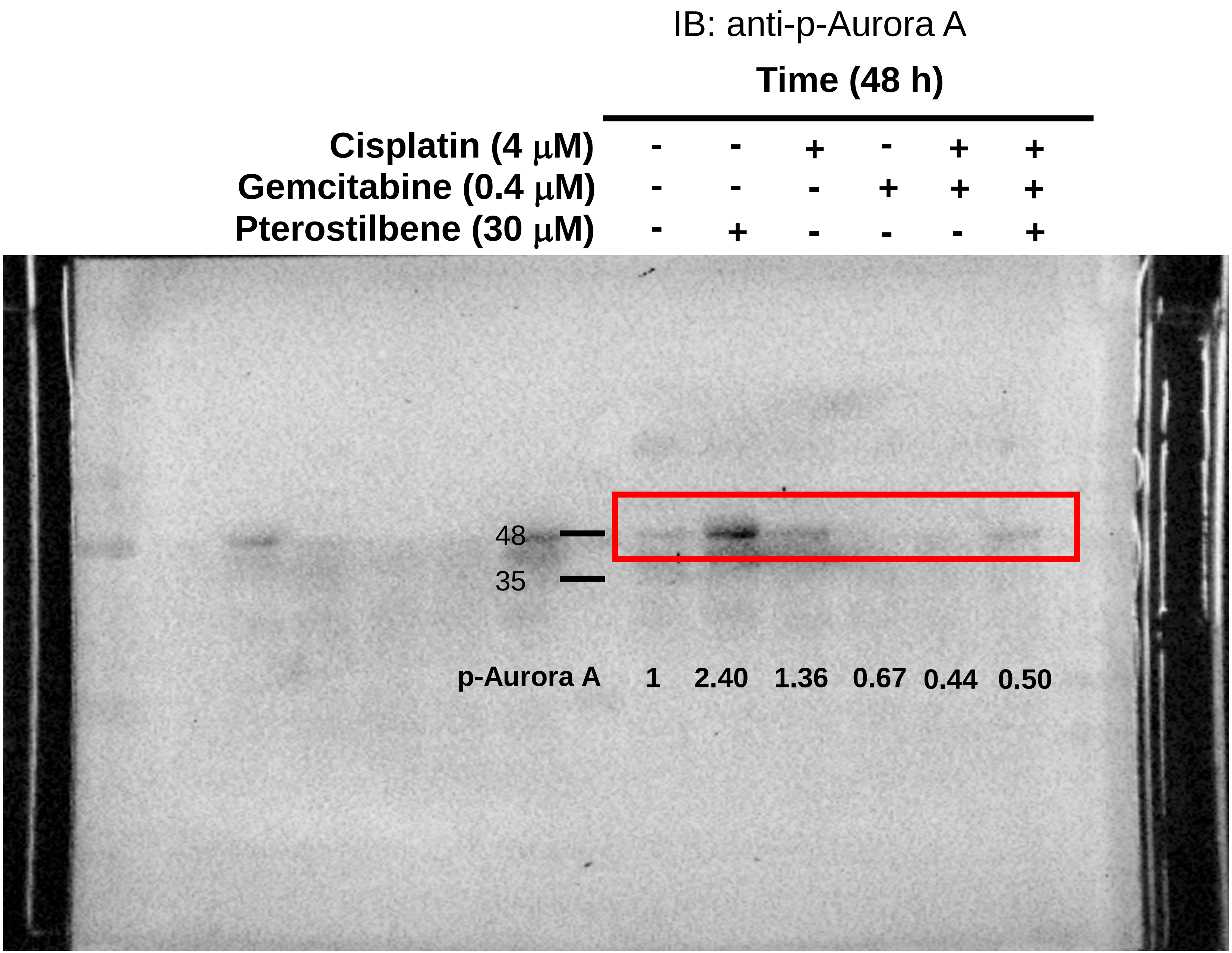

Fig. 5E Cis+Gem+PS_beta actin (p-Aurora A)

IB: anti-β actin

Time (48 h)

**Cisplatin (4 μM)**	-	-	+	-	+	+
**Gemcitabine (0.4 μM)**	-	-	-	+	+	+
**Pterostilbene (30 μM)**	-	+	-	-	-	+

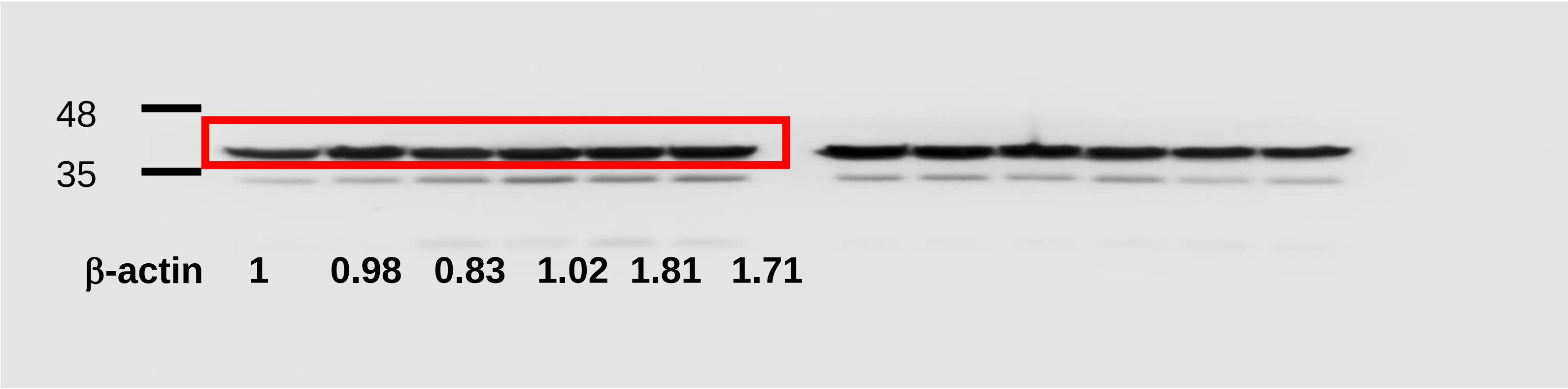

Fig. 5E Car+Gem+PS_Pan-Ras

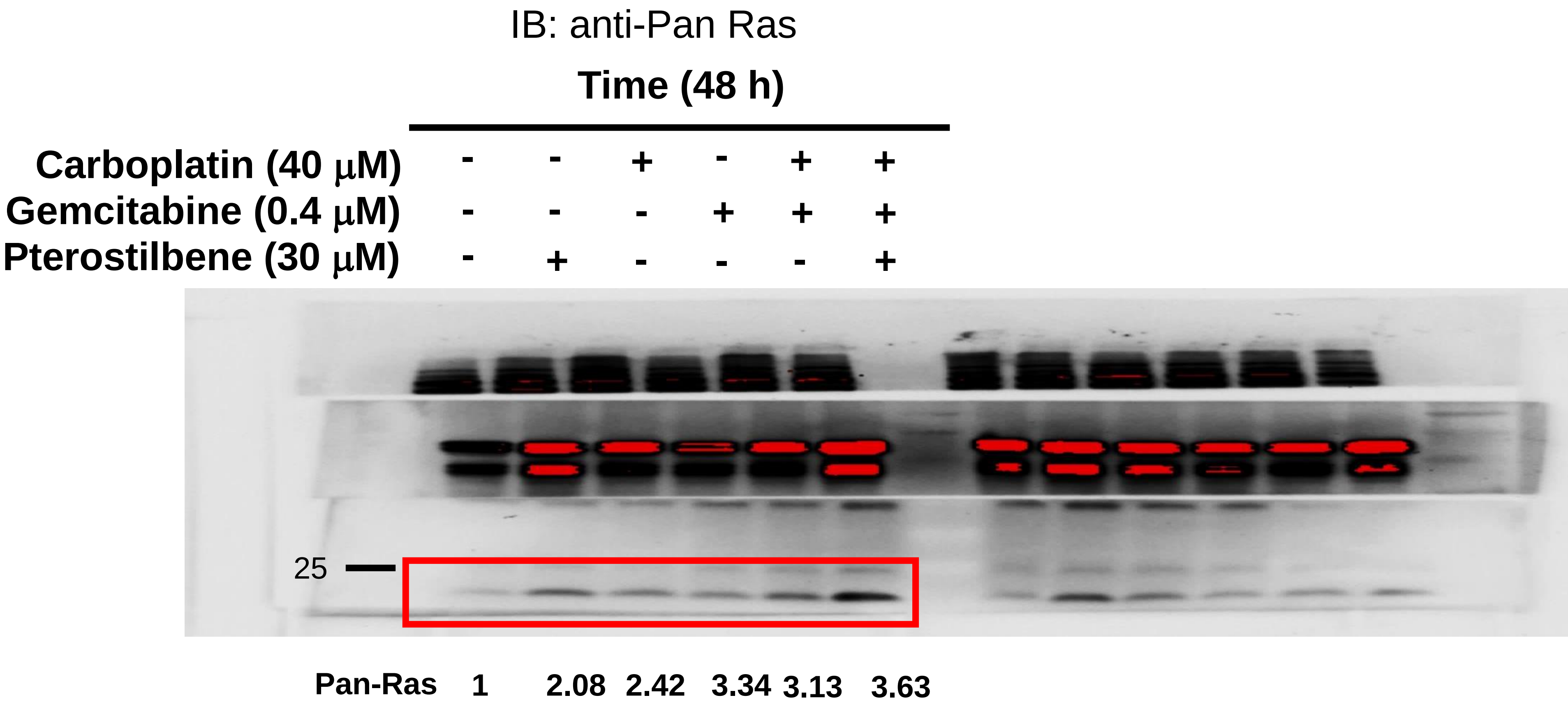

Fig. 5E Car+Gem+PS_p-RB
IB: anti-p-RB

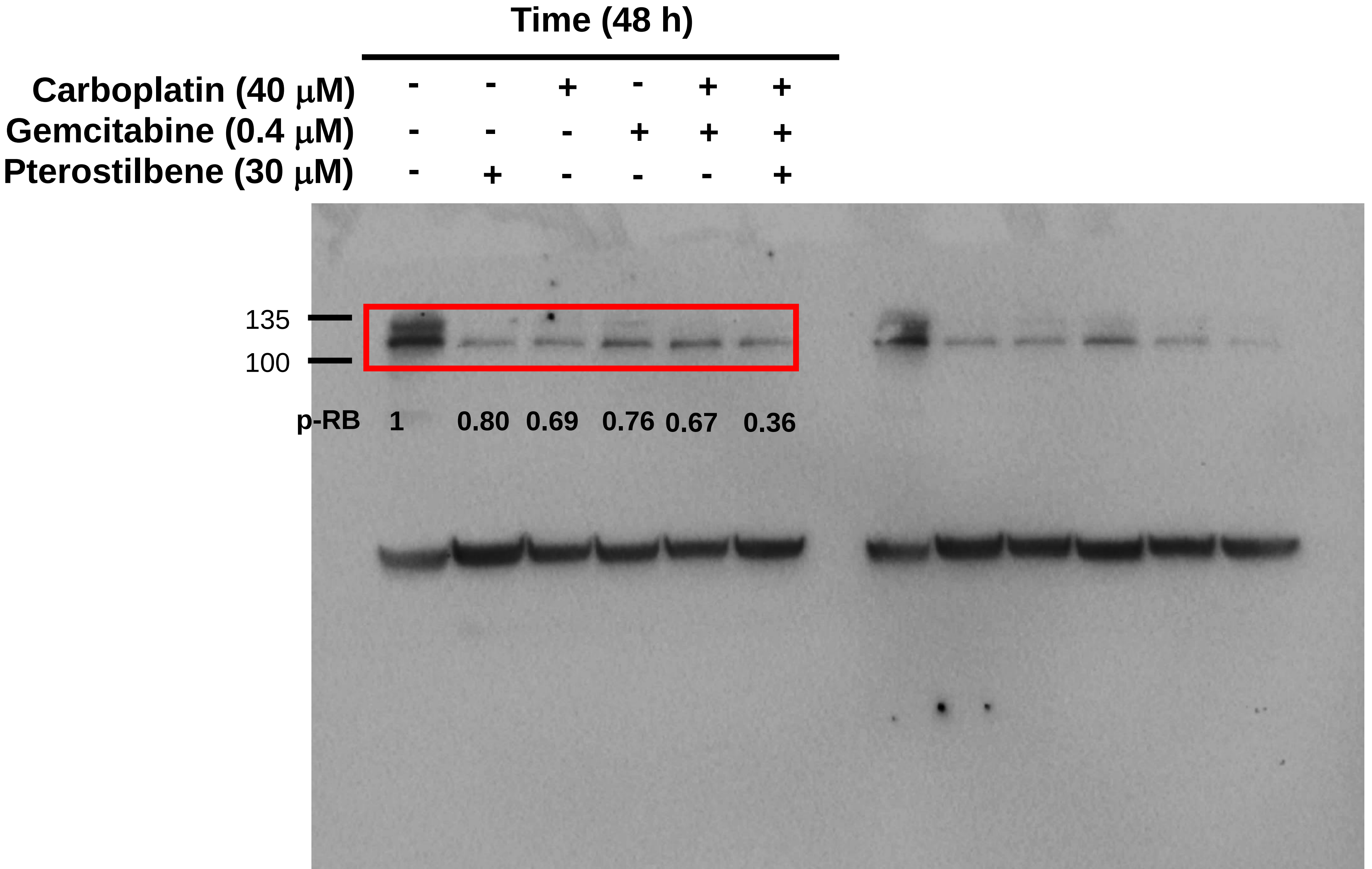

Fig. 5E Car+Gem+PS_beta actin (Pan-Ras, p-RB)

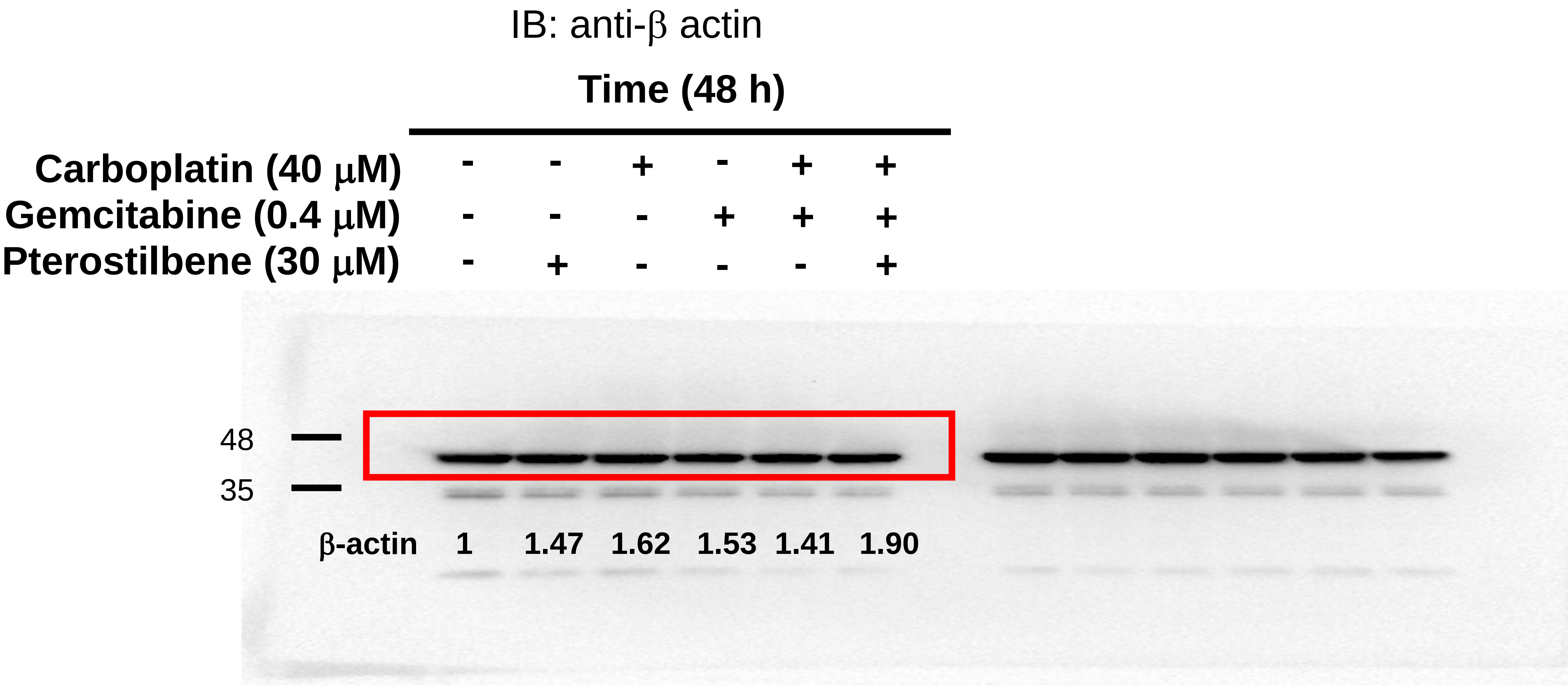

Fig. 5E Car+Gem+PS_p-Aurora A

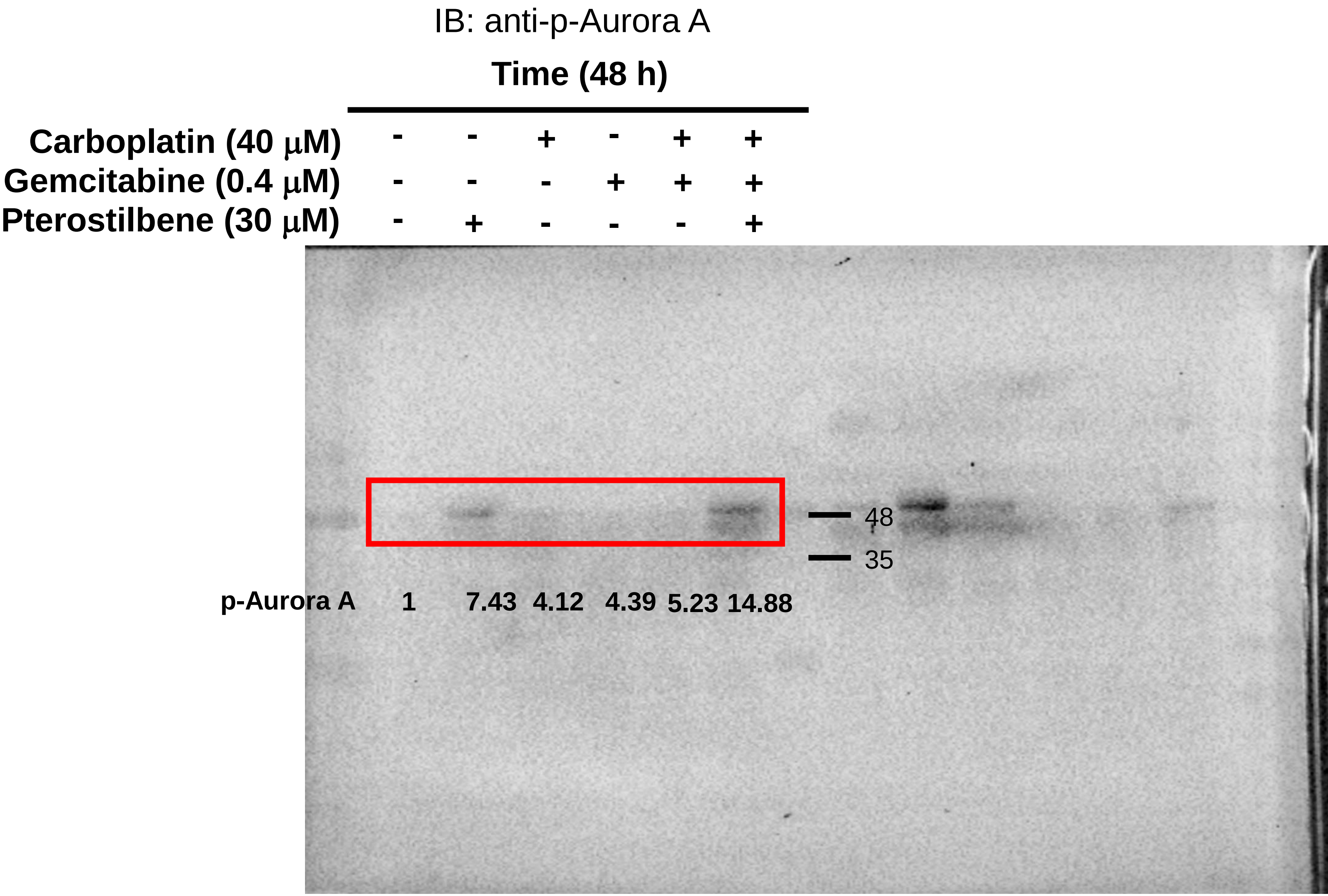

Fig. 5E Car+Gem+PS_beta actin (p-Aurora A)

IB: anti-β actin

Time (48 h)

**Carboplatin (40 μM)**	-	-	+	-	+	+
**Gemcitabine (0.4 μM)**	-	-	-	+	+	+
**Pterostilbene (30 μM)**	-	+	-	-	-	+

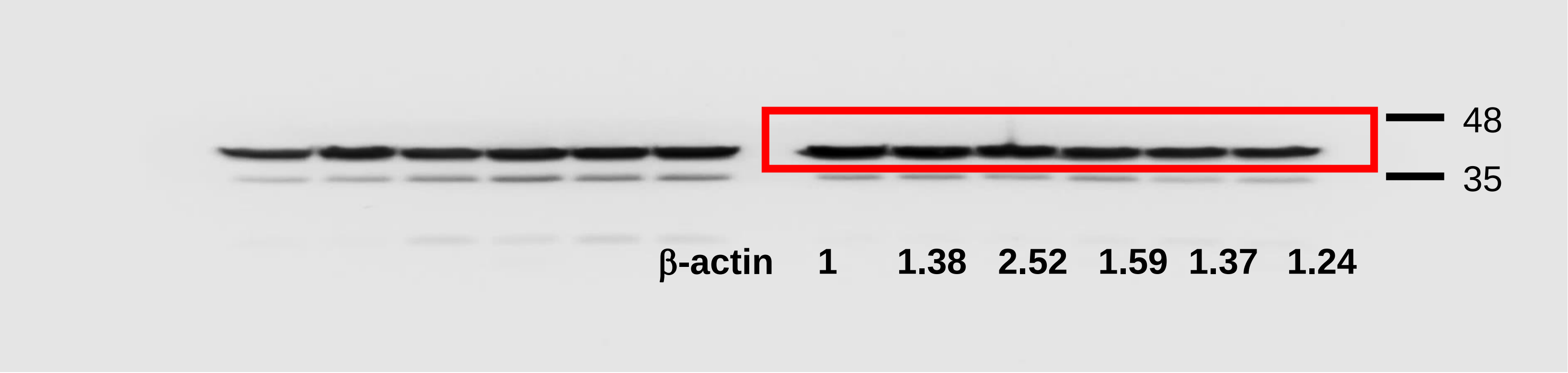

Fig. 6F tumor_p-Class I PI3K

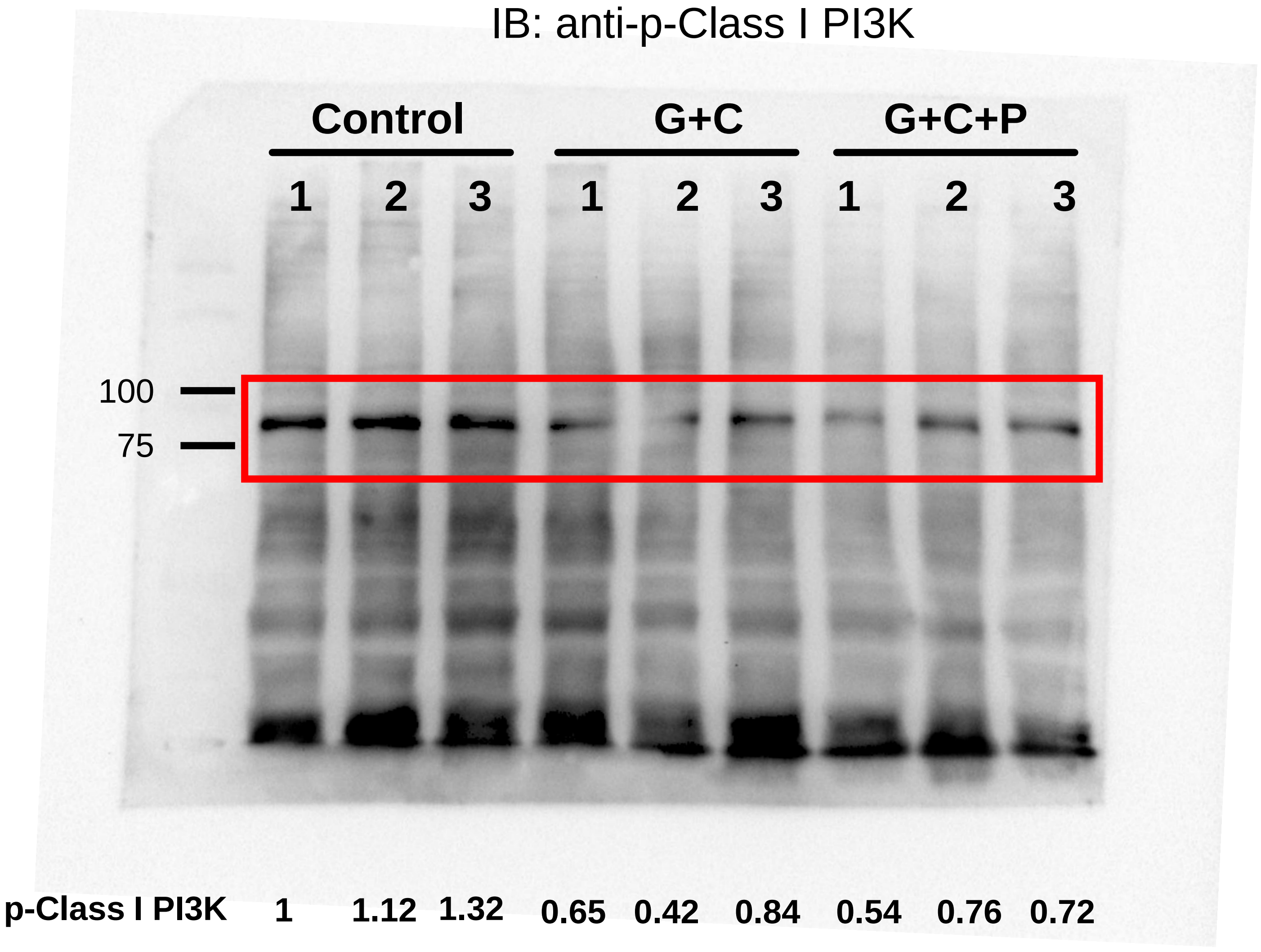

Fig. 6F tumor_p-mTOR

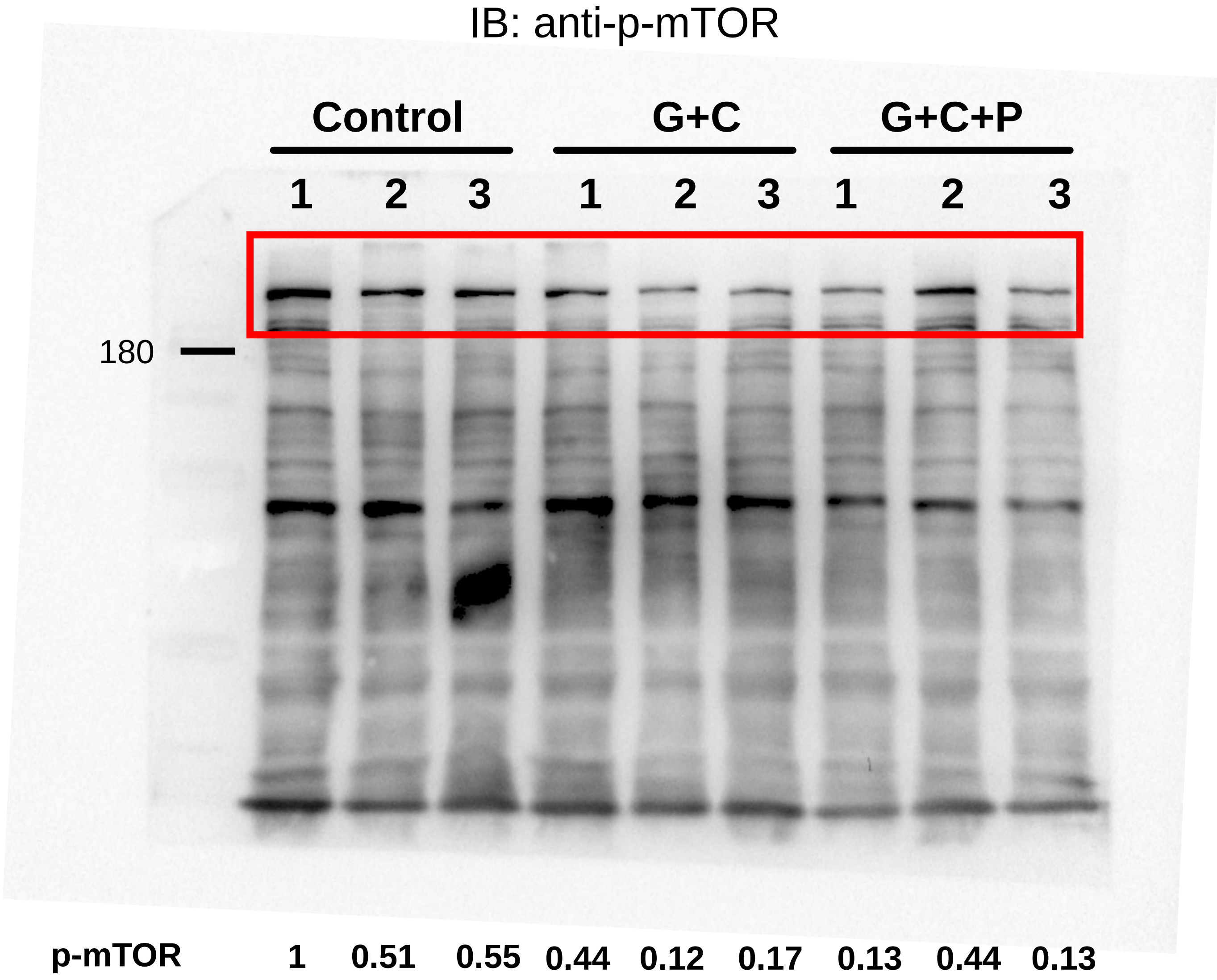

Fig. 6F tumor_p-p70S6K

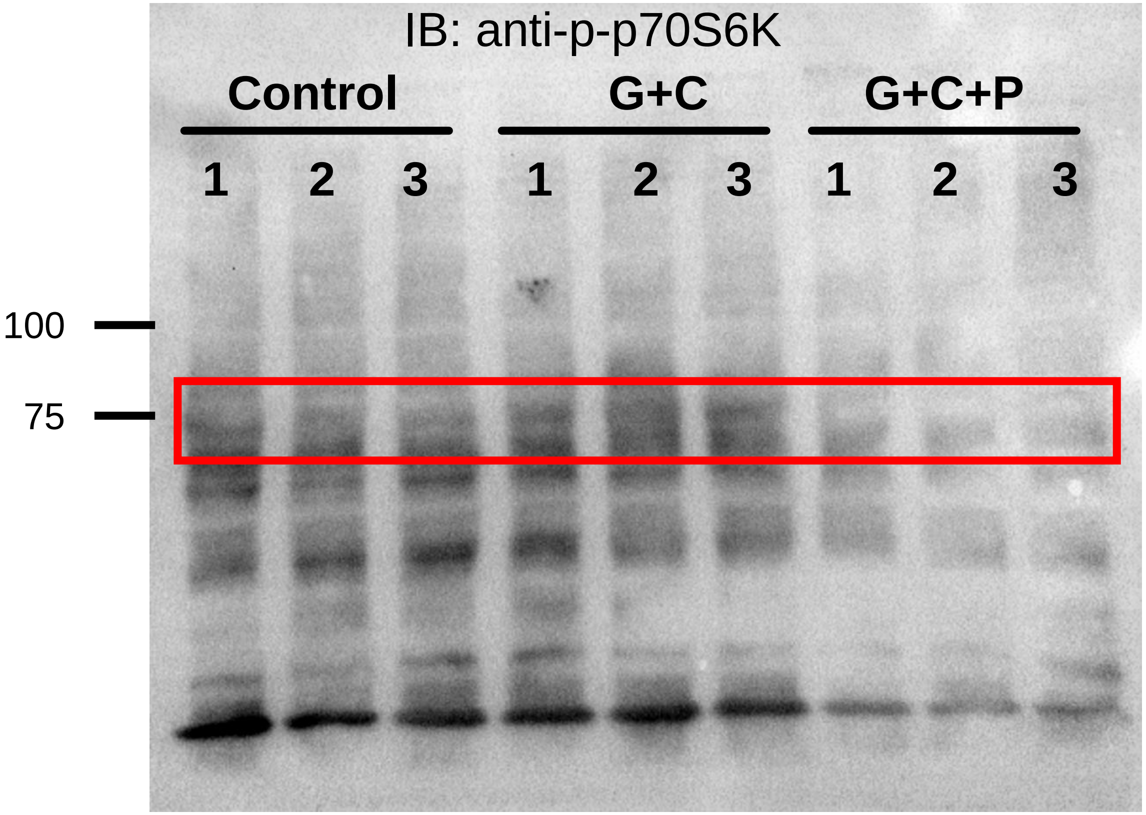

**p-p70S6K**	**1**	**1.59**	**2.01**	**2.01**	**1.79**	**1.44**	**0.73**	**0.43**	**0.44**

Fig. 6F tumor_p-MEK

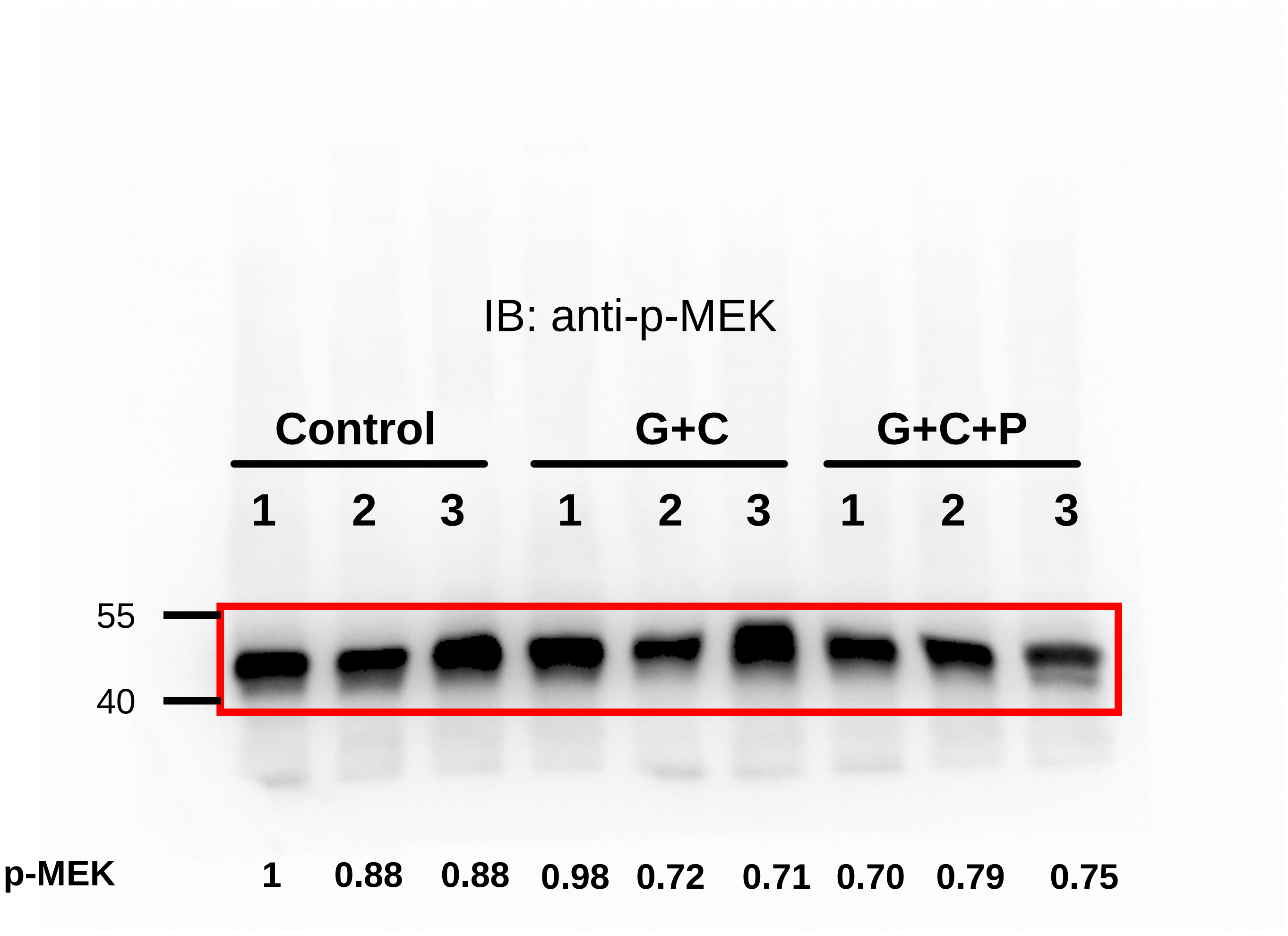

Fig. 6F tumor_p-ERK

IB: anti-p-ERK

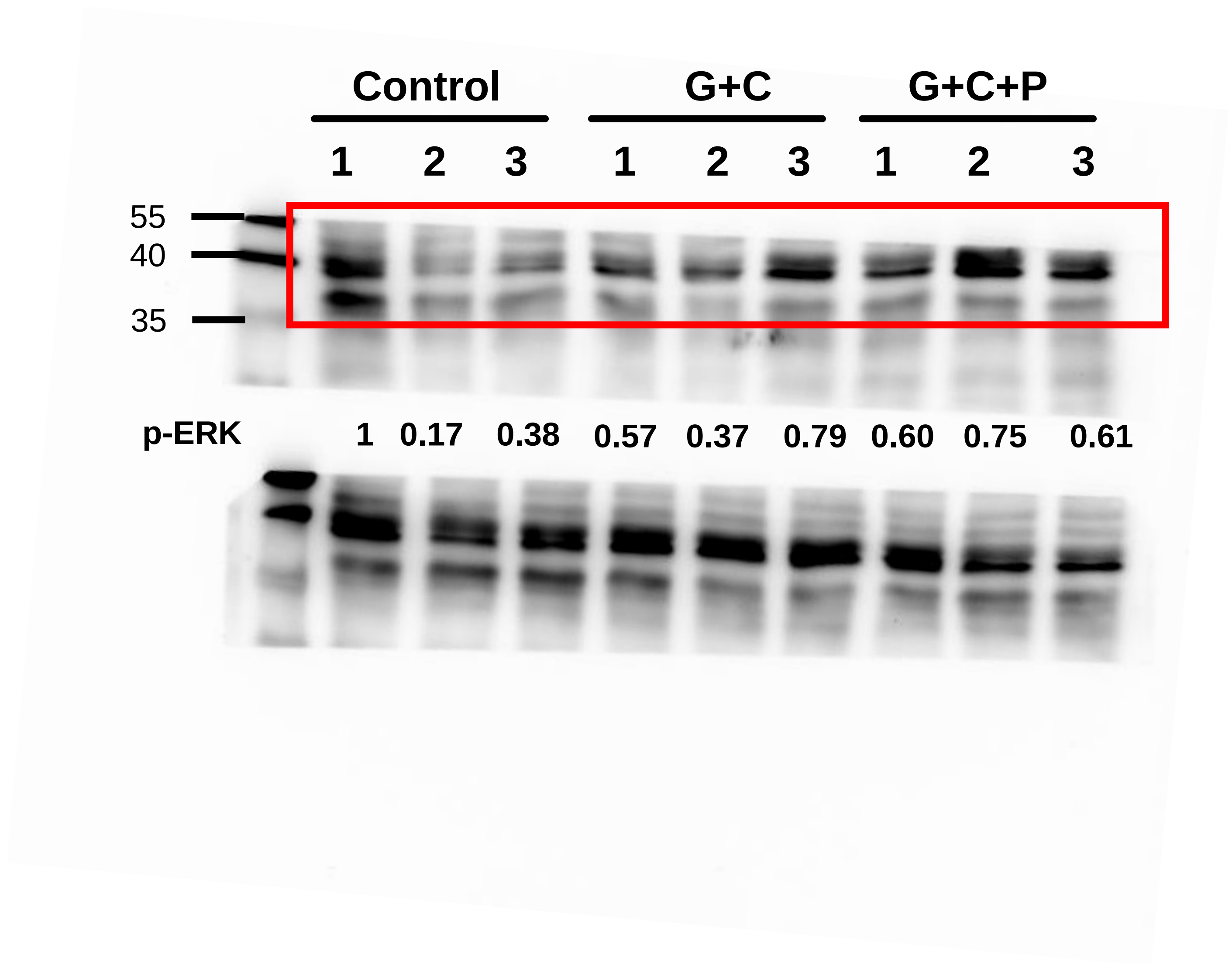

Fig.6F tumor_beta actin

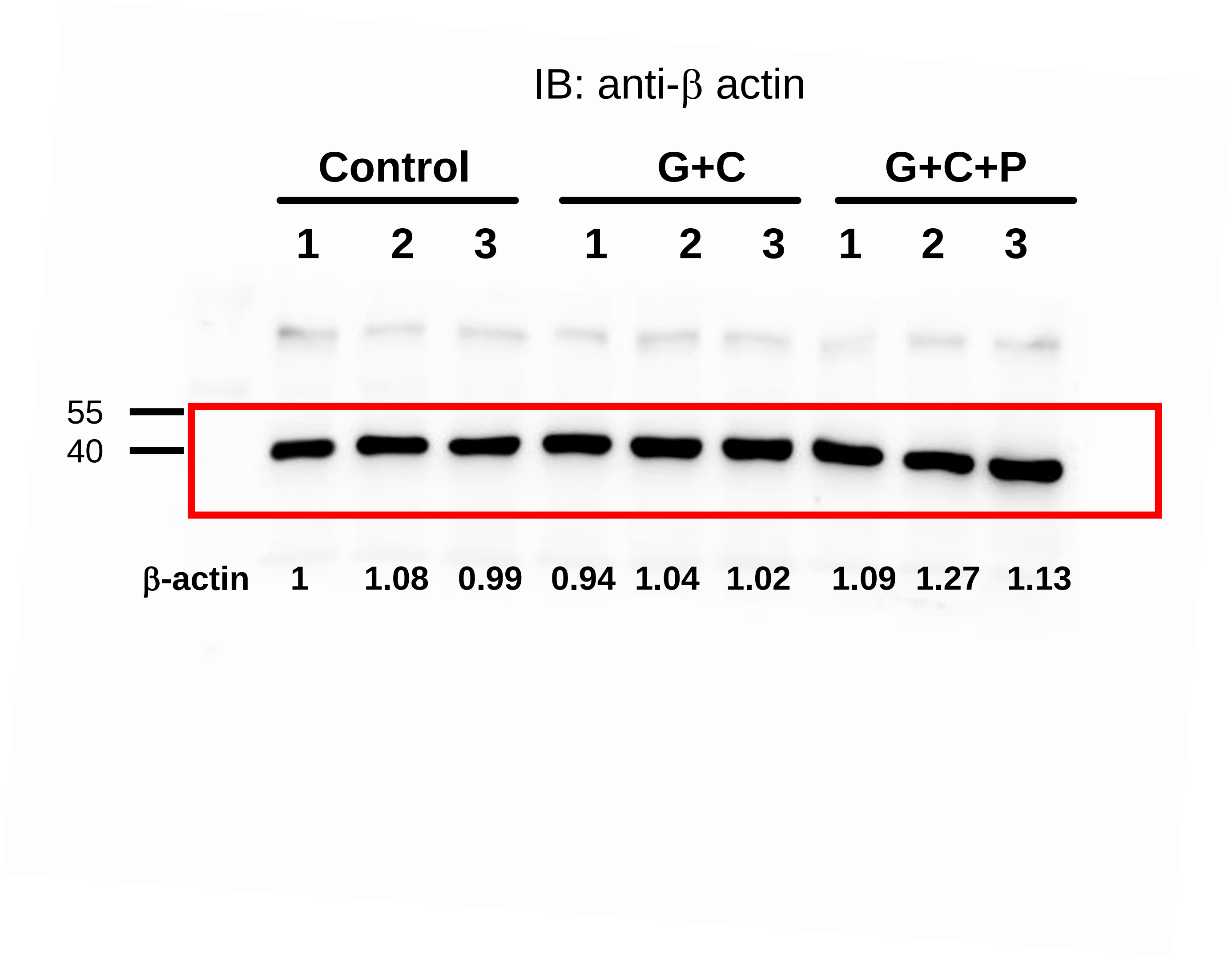

Fig. S8 T24, E7_p-RB

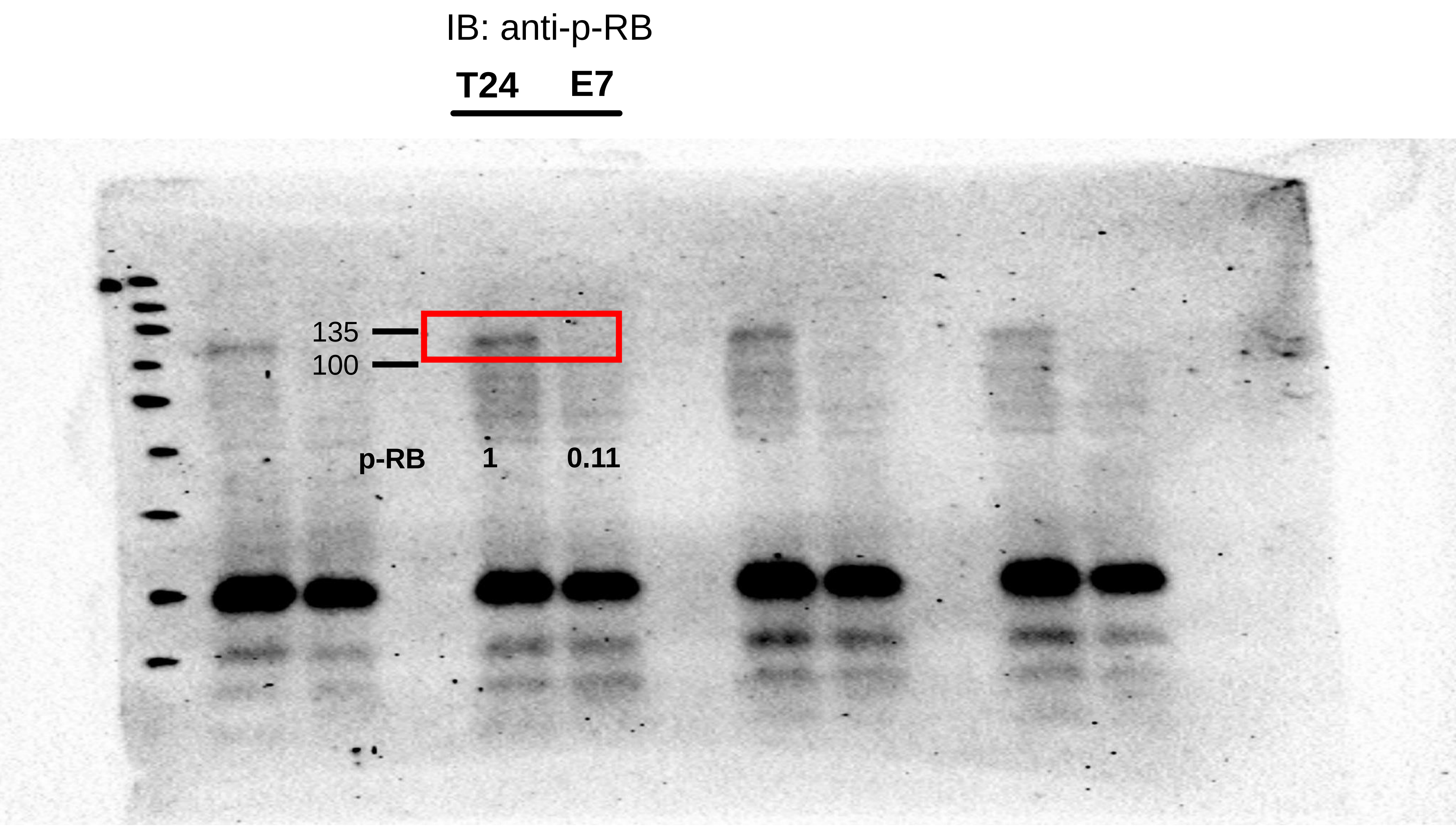

Fig. S8 T24, E7_beta actin (p-RB)

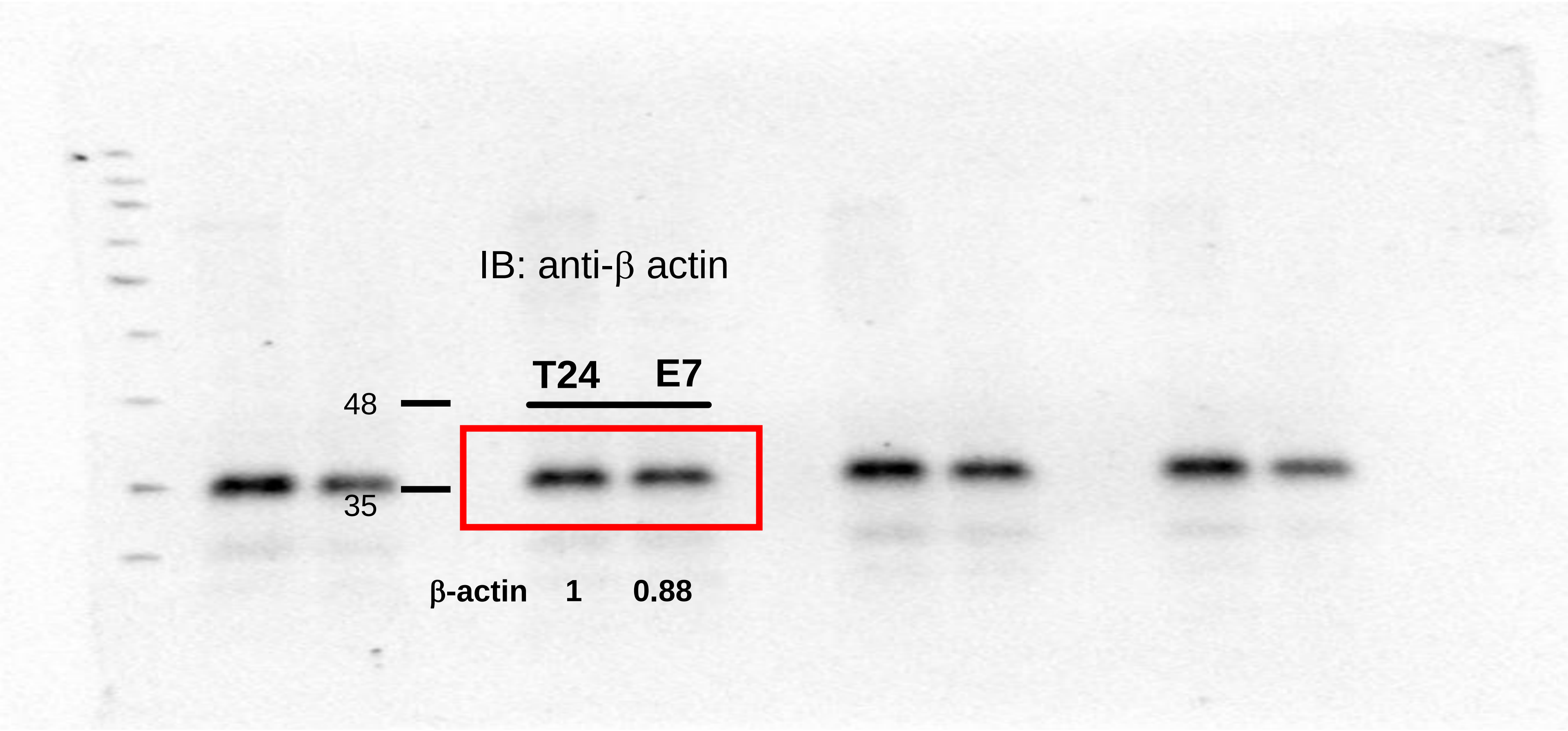

Supplement: Supplementary file 1 [file cancers-12-02869-s001.zip › Figure. S9_Uncropped blots.pdf]
